# Comparative transcriptomic analysis on compatible/incompatible grafts in *Citrus*

**DOI:** 10.1093/hr/uhab072

**Published:** 2022-01-19

**Authors:** Wen He, Rui Xie, Yan Wang, Qing Chen, Hao Wang, Shaofeng Yang, Ya Luo, Yong Zhang, Haoru Tang, Frederick G Gmitter, Xiaorong Wang

**Affiliations:** 1 College of Horticulture, Sichuan Agricultural University, Chengdu 611130, Sichuan, China; 2 Institute of Pomology and Olericulture, Sichuan Agricultural University, Chengdu 611130, Sichuan, China; 3 Citrus Research and Education Center, University of Florida, Lake Alfred 33850, FL, USA

## Abstract

Grafting is a useful cultivation technology to resist abiotic and biotic stresses and is an integral part of citrus production. However, some widely utilized rootstocks may still exhibit graft incompatibility in the orchard. ‘Hongmian miyou’ (*Citrus maxima* (Burm.) Merrill) is mutated from ‘Guanxi miyou’, but these two scions showed different compatibility with available *Poncirus trifoliata* rootstock. Foliage etiolation is an observed symptom of graft incompatibility, but its mechanism remains poorly understood. This study is the first to investigate the morphological, physiological, and anatomical differences between compatible and incompatible grafts, and perform transcriptome profiling at crucial stages of the foliage etiolation process. Based on comprehensive analyses, hormonal balance was disordered, and two rate-limiting genes, *NCED3 (*9-*cis*-epoxycarotenoid dioxygenase 3) and *NCED5*, being responsible for ABA (abscisic acid) accumulation, were highlighted. Further correlation analysis indicated that IAA (indole-3-acetic acid) and ABA were the most likely inducers of the expression of stress-related genes. In addition, excessive starch accumulation was observed in the lamina and midribs of leaves of incompatible grafts. These results provide a new insight into the role of hormonal balance and ABA biosynthesis genes in regulating and contributing to graft incompatibility, and will further define and deploy candidate genes to explore the mechanisms underlying citrus rootstock–scion interactions.

## Introduction

Plant grafting has been used for millennia to reduce juvenility and confer biotic and abiotic stress tolerance [1–3]. Citrus production is largely dependent on the rootstock used for grafting of scion cultivars [4]. Although grafting is widely used in citrus production, some widely utilized rootstocks may still exhibit graft incompatibility in the orchard and this reaction can take years to manifest [2]. For example, Swingle citrumelo (*Citrus paradisi* × *Poncirus trifoliata*) is characterized by its tolerance to biotic stress and the superior fruit quality it confers on the scion, but is incompatible with some sweet orange clones (*Citrus sinensis*) [5]. Trifoliate orange (*P. trifoliata*) is a commonly used rootstock in the citrus industry due to its superior resistance to several abiotic and biotic stresses [6, 7], while it is incompatible with some varieties of lemon (*Citrus limon*) [8] and pummelo (*Citrus maxima*) [9]. With the rapid replacement of citrus varieties in recent years, bud union incompatibility has become a factor that limits the development of the citrus industry [10]. However, the reasons for graft incompatibility are still shrouded in mystery.

During grafting, tissues from the scion cultivar are brought into fusion with the rootstock to form one composite organism [1, 11, 12]. Plant genomes may interact when genetically distinct individuals join together. Grafting systems have been documented for decades and are important platforms for exploring the signals between scion and rootstock [13–15]. *MdWRKY9* acted as a candidate gene for dwarfing control in apple [16], and transfer of small interfering RNAs (siRNAs) enhanced disease resistance of the sweet cherry scion [17]. *PbWoxT1* (WUSHEL-RELATED HOMEOBOX *WOX*) underwent long-distance transport via the phloem and was involved in various physiological processes in pear scion [18]. The rootstock can also cause DNA demethylation and a reduction in 24-nucleotide small RNAs (sRNAs) in the scions [6]. Despite such progress, little is known about the mechanism of graft incompatibility in citrus, and graft incompatibility often occurs several years after grafting [10, 19]. ‘Guanxi miyou’ (*C. maxima* (Burm.) Merrill) is compatible with the widely used trifoliate orange (*P. trifoliata*), whereas its mutation ‘Hongmian miyou’ (*C. maxima* (Burm.) Merrill) is incompatible [9]. Previous studies have highlighted their unique features, such as the closely related genetic background, as well as incompatibility symptoms [9, 20, 21]. Graft incompatibility in Hm/Pt (‘Hongmian miyou’ grafted onto trifoliate orange) universally demonstrates the symptoms of foliage etiolation, which occurs ~5 months after grafting. Therefore, the graft-compatible combination Gx/Pt (‘Guanxi miyou’ grafted onto trifoliate orange) and the incompatible combination Hm/Pt can be considered as potential model graft combinations for citrus research.

In this study, we performed transcriptome profiling and validated the morphological, physiological, biochemical, and anatomical differences between compatible and incompatible grafts during the foliage etiolation process. Our objectives are to exploit the material basis, gene expression patterns, and illustrate the co-expressed networks that play regulatory roles in *Citrus* incompatible grafts during foliage etiolation. The large-scale, multi-strategy datasets that are produced will provide valuable resources for understanding the mechanism of graft incompatibility in *Citrus* and the application of heterografting in horticultural plants.

## Results

### Morphological development and transcriptome profiling

**Figure 1:**
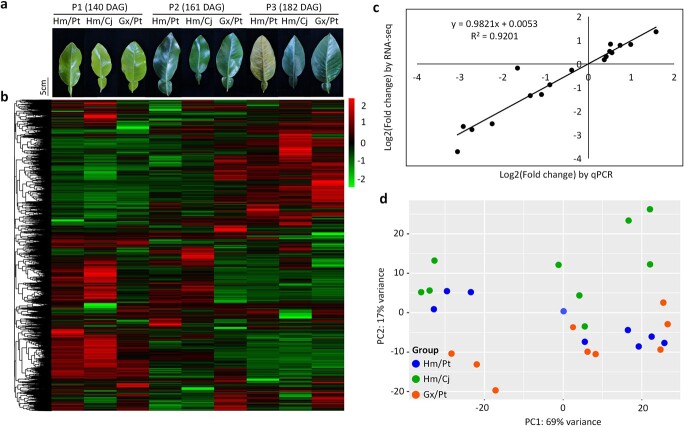
Transcriptome analysis of leaves. **a** Leaf samples at three phases (P1, P2, P3). **b** Hierarchical clustering of unigene expression. **c** Correlation of expression changes observed by RNA-seq (*Y*-axis) and qPCR (*X*-axis). **d** PCA of the samples sequenced by RNA-seq. The *X*-axis and *Y*-axes represent the first and second components. Dots with the same color indicate the same graft combination.

Since graft-incompatible symptom foliage etiolation took ~5 months after grafting [9, 20, 21] (Supplementary Fig. S1a), we investigated plant growth and physiological indices at crucial development stages: 112, 140, 160, 182 and 203 days after grafting (DAG) (Supplementary Fig. S1b). Hm/Pt exhibited the lowest SPAD value (which was correlated with leaf chlorophyll content), water potential, and water content at 182 and 203 DAG (Supplementary Fig. S1). As for mineral elements, copper (Cu) content was significantly higher than in controls at 140 and 161 DAG, but significantly lower than in controls at 182 and 203 DAG. The contents of manganese (Mn), iron (Fe), and zinc (Zn) in Hm/Pt were lower than in controls at 182 DAG (Supplementary Fig. S2). The trends of five phytohormone levels, including abscisic acid (ABA), indole-3-acetic acid (IAA), gibberellin A3 (GA_3_), jasmonic acid (JA), and salicylic acid (SA), were further determined (Supplementary Fig. S3). The content of IAA was significantly lower than in controls at 161 and 182 DAG, and ABA was much higher than in controls at 182 and 203 DAG. In addition, we observed anatomical characteristics using transverse sections of the leaves at 112, 140, and 161 DAG (Supplementary Fig. S4). Considering there was no significant difference between samples at 112 and 140 DAG (Supplementary Fig. S4), and the leaves at 203 DAG had completely etiolated (Supplementary Fig. S1a), we finally sampled leaves at 140, 161, and 182 DAG (P1, P2, and P3, respectively) with three biological replicates for subsequent analyses (Fig. 1a).

The transcriptional profile differences between graft-compatible and -incompatible combinations were compared using RNA-Seq (Supplementary Table S1). A total of 16 482 genes were found to be expressed in at least one sample after reducing transcriptional noise (Fig. 1b). Principal component analysis (PCA) of all samples was performed to understand the transcriptional dynamics of the development of combinations (Fig. 1d). The results revealed a clear distinction between samples at P1, P2, and P3. Differentially expressed gene (DEG) analysis was also conducted with the sequencing data. In addition, real-time quantitative PCR (RT-qPCR) was utilized to validate the results using 10 randomly selected genes. The high correlation coefficient (*R*^2^ = .9) between the qPCR and RNA-seq results indicated a high level of reproducibility and reliability of the data (Fig. 1c; Supplementary Table S2).

### Differentially expressed gene analyses

**Figure 2:**
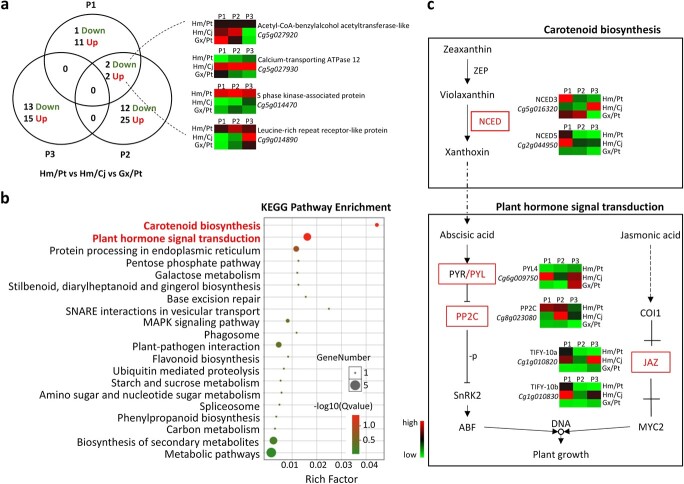
DEG analyses. **a** Venn diagrams of genes differentially expressed between Hm/Pt and controls at the same developmental stage. **b** KEGG enrichment analysis of the DEGs. **c** Expression of DEGs in carotenoid biosynthesis and plant hormone signal transduction pathways. Heatmap color indicates FPKM value.

Pairwise comparison between Hm/Pt and controls (Hongmian miyou' grafted onto C. junos, Hm/Cj and Gx/Pt), at the same development stage, were performed to explore the mechanism of graft incompatibility. A total of 81 DEGs were identified in the three comparisons (Fig. 2a; Supplementary Table S3). This number was fewer than that of DEGs (164 genes) from pairwise comparison between the different development stages within the same combination (Supplementary Fig. S5). Among the DEGs at P1, P2, and P3, most genes were up-regulated in Hm/Pt (13/16, 27/41, and 15/28 at P1, P2, and P3, respectively).

To gain insights into the main metabolic pathways involved in the considered comparisons, we carried out a standard enrichment analysis based on KEGG (Kyoto Encyclopedia of Genes and Genomes) pathways. The DEGs were significantly enriched in many pathways, such as ‘carotenoid biosynthesis’ and ‘plant hormone signal transduction’ (Fig. 2b). Hm is a bud mutant of Gx, which shows different carotenoid content in fruit peel tissues. Hormones have been shown to play important roles in plant growth. Six DEGs (two NCED genes, *Cg5g016320* and *Cg2g044950*; one ABA receptor, PYL, *Cg6g009750*; one protein phosphatases type 2C, PP2C, *Cg8g023080*; and two TIFY family genes, *Cg1g010820* and *Cg1g010830*) were detected in these two pathways. Their transcription levels are presented as a heatmap for both Hm/Pt and controls in Fig. 2c. The transcript levels of *NCED3* and *PP2C* were higher in the Hm/Pt combination than in controls at P1. The former sharply decreased at P2. In addition, *NCED5* showed down-regulation at P2 and P3. The expression of *PYL4* and two *TIFY* family genes was less in Hm/Pt than in controls at P3.

### Weighted gene co-expression network analysis

**Figure 3:**
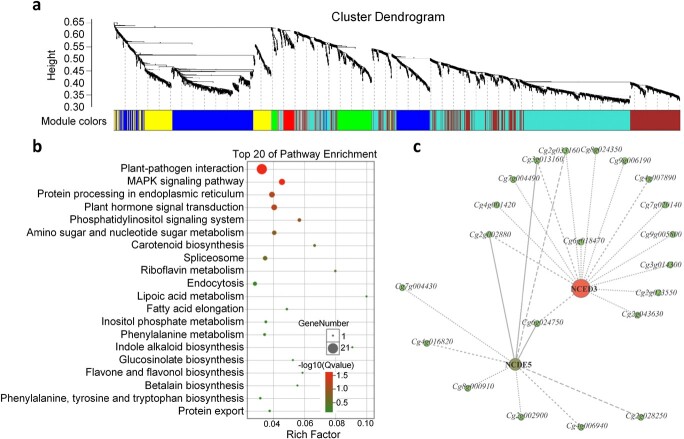
WGCNA of differentially expressed genes. **a** Hierarchical cluster tree showing co-expression modules identified by WGCNA. Each leaf in the tree is one gene. The major tree branches constitute seven modules labeled with different colors. **b** KEGG enrichment analysis of the genes in the blue module. **c** Genes whose expression was highly correlated in the blue module.

An alternative analysis tool, weighted gene co-expression network analysis (WGCNA) [22], was adopted in this study. Modules were defined as highly connected gene clusters; genes in the same cluster had a high correlation coefficient with each other. After screening for differentially expressed genes by FPKM (fragments per kilobase of transcript per million mapped reads), 2705 genes were used for WGCNA, resulting in seven different modules (marked with different colors in Fig. 3) (Fig. 3a, Supplementary Table S4). We focused on KEGG pathways of all modules (Supplementary Fig. S6). A total of 476 genes [including 40 transcription factors (TFs)] were aggregated in the blue module (Supplementary Table S5), including *NCED3*, *NCED5*, *PP2C*, TIFY family genes, and *PYL4*. In addition, the mineral elements, phytohormone contents, and starch and sugar contents were selected as traits for assessing the correlation with WGCNA modules. Modules having a correlation with the phytohormone contents with an absolute coefficient ≥.8 were chosen. The green module (*P* = 3 × 10^−9^, *cor* = .87) was found to be related to the total ABA content (Supplementary Fig. S7). Interestingly, this module was also related to Cu content (*P* = 2 × 10^−6^, *cor* = .78). Gene ontogeny (GO) enrichment analysis of these 143 genes in the module showed enriched GO terms related to cell wall organization (Supplementary Table S6, Supplementary Fig. S8).

### Differential anatomical changes

**Figure 4:**
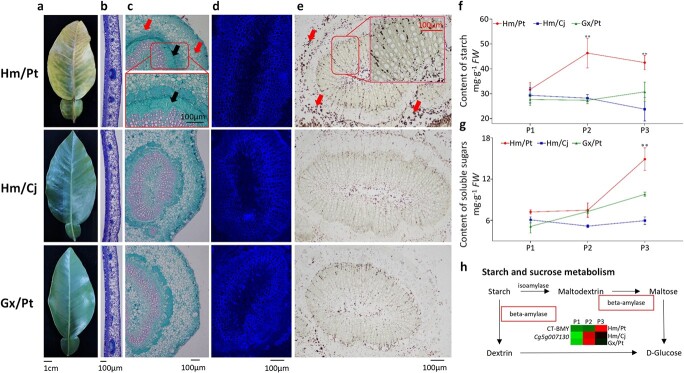
Transverse sections showing changes in leaves of compatible and incompatible grafts. **a** Leaf samples at P3. **b** Cross-section of leaf. **c** A midrib section was observed and photographs were taken under a light microscope. **d** Epifluorescence photomicrographs of phloem. **e** Starch grains were dyed blue. **f**, **g** Contents of starch (**f**) and soluble sugars (**g**). **h** Transcript abundance changes of starch and sucrose metabolism pathways. Asterisks represent significant differences compared with the control (**P < .01), analyzed using Student’s *t*-test. Heatmap shows the log_10_ (FPKM + .01) of selected differentially expressed transcripts. Black arrows indicate parenchyma cells and red arrows indicate starch accumulation in **c** and **e**.

To further determine the anatomical modification in the incompatible graft of Hm/Pt, we inspected the anatomical characteristics using transverse sections of the leaves at P3 (Fig. 4a and b). The results showed that phloem collapse with cell wall distortion and thickening in the prominent midribs were much more frequently observed in Hm/Pt (Fig. 4c). Additionally, phloem plugging is one of the main reasons for leaf etiolation [23], but it was not found in Hm/Pt (Fig. 4d). Intriguingly, very little or no starch accumulation was detected in Hm/Cj and Gx/Pt, but starch accumulated in the internal structure of the lamina in Hm/Pt (Fig. 4e and f). Spatially, these excessive starch grains in the midribs were located analogously to the lamina in the epidermis and mesophyll cells (palisade and spongy parenchyma cells) (Fig. 4c and e).

## Discussion

### Rootstocks can affect scion growth at the molecular level

Although rootstocks are an integral part of commercial citrus production systems, their influence on a grafted scion at the molecular level has not been thoroughly investigated [2]. In this study, a limited number of DEGs were detected, which might be explained by the highly similar genetic background between the scions. Also, the rootstock might affect the scions, though only a few genes. We noticed that the number of DEGs was also limited in other graft combinations [24, 25], which was consistent with our results. Rootstock-induced modifications to shoot architecture seem to be influenced by a few genes, as observed in rootstock-induced dwarfing in apple [16, 26, 27]. Our study supported the impact of the rootstock on metabolite accumulation in scion growth through modulation of gene expression. Three hundred and sixty-one DEGs were observed between Hm/Pt and Hm/Cj, including 11 TFs (Supplementary Table S7, Supplementary Fig. S9). They were *UNE12* (unfertilized embryo sac 12), *SRM1*, three MYBs (*MYB14*, *MYB15*, *MYB108*), *bHLH3*, *Hsf4* (heat stress transcription factor 4), and four ERFs (*ERF71*, *CRF5*, *ERF1* and *RAV1*). *UNE12* as a temperature-responsive SA immunity regulator, and *SRM1*, *MYB*s, *bHLH3*, *ERF*s and *Hsf4* showed a variety of stress-regulated expression patterns [28–30], indicating that the rootstock may regulate scion phenotypes by regulating these TFs.

### Hormone IAA/ABA balance triggers stress response

An important substance involved in the development of compatibility is auxin [31–34]. In this study, etiolation development in Hm/Pt was closely related to the content of IAA (Supplementary Fig. S3). Several genes related to the auxin-induced and -responsive pathways were differentially expressed, including WAT1 [35] (*Cg6g003960*), GLIP2 [36] (*Cg2g006520*) and SUR2 [37] (*Cg7g017070*). Moreover, we found that the expression of GH3 (*Cg6g020710*, *Cg5g036250*), which regulated IAA levels by binding excess IAA to amino acids [38], showed responses to foliage etiolation. Two TIFY 10A-like genes, *Cg1g010820* and *Cg1g010830*, showed significantly different expression dynamics (Fig. 2c). They were reported to be independent of the JA signaling pathway but controlled by the auxin/IAA–auxin response TF signaling pathway [39]. This might explain why JA levels were not significantly regulated (Supplementary Fig. S3). It has been documented that the TIFY family proteins JAZ4 and JAZ8 competitively interact with WRKY57 to mediate leaf senescence [40].

By combining the physiological and biochemical data and the transcriptome profiling analyses results, we found one main pathway cascade, which started from ‘carotenoid biosynthesis’ leading to the accumulation of ABA and to ‘plant hormone signal transduction’. The ABA receptor (*Cg6g009750*) and PP2C (*Cg8g023080*) as signals can induce stress-related pathways, such as ‘MAPK signaling pathway’, ‘plant–pathogen interaction’ and ‘starch and sucrose metabolism’ (Fig. 3). The whole pathway cascade was transcriptionally activated in Hm/Pt during leaf development, with two rate-limiting NCED enzyme genes [41, 42], *NCED3* (*Cg5g016320*) and *NCED5* (*Cg2g044950*). Previous evidence from the commercial pummelo has shown that flesh color formation is due to carotenoid accumulation [43–45]. However, the results showed no obvious expression dynamics in all unigenes that might be involved in carotenoid biosynthesis, except *NCED*s (Supplementary Table S8).

**Figure 5:**
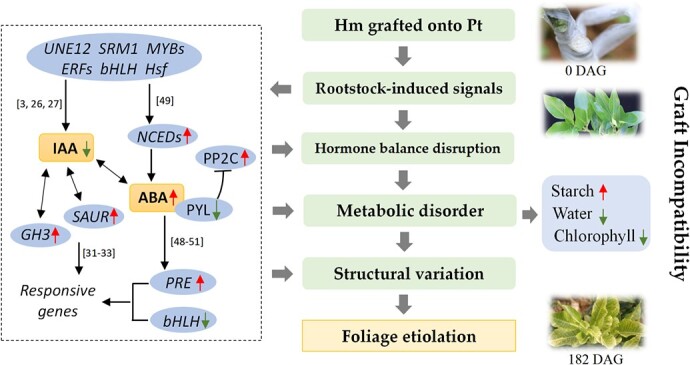
Working model for graft incompatibility in Hm/Pt. The rootstock–scion interaction induced signals to cause TF gene activity. Differential expression of TFs could directly affect auxin and ABA signal transduction. Genes related to auxin and ABA were differentially expressed, causing a decrease in IAA and an increase in ABA level, and further acclimation to grafting-induced stress, such as water deficiency, starch accumulation and chlorophyll decreased. Metabolic disorder caused structural variation, and led to foliage etiolation. The red and green arrows indicate up- and down-regulation respectively. Square brackets contain reference numbers.

*UBX domain-containing protein 8* (*Cg5g042690*) and *pectinesterase-like* (*Cg5g037690*) were identified as hub genes in the green module by using WGCNA (Supplementary Fig. S10), which played an important role in oxidative stress and the osmotic stress response [46], and functions in cell wall metabolism [47], respectively. The modification of amyloplasts, which are the energy stores within the plant cell that store carotenoids, results in starch biosynthesis and carotenoid biosynthesis competing for carbon, which leads to the negative association reported in citrus [48]. Furthermore, we found that one starch biosynthesis-related gene, *Cg5g007130*, encoding β-amylase [48], was significantly down-regulated in Hm/Pt (Fig. 4h). Sugars are derived from the degradation of chloroplastic transitory starch. High levels of sugar content also act as regulatory signals, which can trigger a cessation of growth and the induction of catabolic processes. These may cause the phloem collapse with cell wall distortion and thickening in Hm/Pt (Fig. 4). Overall, our study showed that hormonal signaling played an important role in the foliage etiolation process.

### Underlying mechanisms of the etiolation process

Based on the results, we propose a working model for the graft incompatibility in Hm/Pt by exploring the mechanism of foliage etiolation (Fig. 5). Phytohormones are the important signals regulating plant growth and development [31]. In recent publications, through comparing the transcriptomes of genetically identical roots that had been grafted onto different scion genotypes, it was found that hormone signal transduction and sugar metabolism genes were highly represented in the DEGs [27, 49]. Here, several genes related to the auxin-induced and -responsive pathways were differentially expressed, and the IAA level was decreased in Hm/Pt (Supplementary Fig. S3). ABA is synthesized in response to multiple abiotic stresses that alter tissue water status [50]. ABA has also been suggested to act as a long-distance signal that moves from the roots via the xylem to the shoot [49, 51], where it restricts transpiration water loss by closing the stomata. In addition, the potential regulatory network showed that the ABA content was highly correlated in the green module, in which genes were related to cell wall organization. Among the TFs, *PRE4 (Cg5g003220)* was highly expressed in Hm/Pt at P3, while *bHLH112* (*Cg2g040950*) was lowly expressed (Supplementary Table S6). Previous studies showed that a mutation in *PRE4/BNQ3* induced decreases in chlorophyll content in *Arabidopsis* [39]. These TFs may also regulate cell wall distortion, starch and sugar accumulation, and leaf etiolation. This evidence, combined with our results, suggested that the decrease in IAA levels and the increase in ABA levels due to the signals between the rootstock and scion may lead to foliage etiolation through a series of physiological and molecular events.

### Conclusions

Grafting is a widely used cultural method in citrus production, but graft incompatibility symptoms, such as foliage etiolation, often appear in orchards. The impact and intrinsic mechanism of graft incompatibility were not well elucidated. In our study, leaf tissues from *Citrus* compatible and incompatible grafts were analyzed. In a comparison, we found that IAA and ABA were the major changed components among the phytohormones. In addition, hormone signal regulators were differentially expressed and played key roles in the foliage etiolation process. This study provides a systematic insight into the differences between incompatible and compatible grafts in leaf metabolites, phytohormones, anatomical structure, and gene expression levels. Our findings will deepen our understanding of the mechanism of graft incompatibility in citrus.

## Materials and methods

### Plant materials and morphological trait measurement

Plant materials were planted in the orchard of Sichuan Agricultural University, Chengdu, China. We used *C. maxima* (Burm.) Merrill cv. ‘Hongmian miyou’ grafted onto *P. trifoliata* (Hm/Pt) as an incompatible graft combination and ‘Hongmian miyou’ grafted onto *Citrus junos* (Hm/Cj) and ‘Guanxi miyou’ grafted onto *P. trifoliata* (Gx/Pt) as compatible graft combinations. All materials were grafted by using the splice grafting method. Each scion/rootstock graft combination was triplicated; 90 replicates (30 trees per block) per combination were considered the minimum acceptable for assessment, although some combinations suffered field losses due to failure of the grafting process.

Growth measurements were made on fully expanded leaves of summer shoots using 10 trees per combination. Leaf chlorophyll concentration was determined using a SPAD-502 Plus meter (Minolta Co. Ltd, Tokyo, Japan). SPAD values are indirectly related to chlorophyll concentration. According to the SPAD values, we divided the etiolation process into five time points (Supplementary Fig. S1). Stem circumferences, total lengths, and internode lengths of summer shoots were measured on the same day. Leaf water potential was measured in 30 leaves from 10 plants of each graft combination by using a Scholander pressure chamber (3005 Series, Soil Moisture Equipment Co. Ltd, Goleta, CA, USA). The cut leaves were immediately enclosed in plastic bags and soil water potential (*Ψ*) determination was initiated within a minute of their collection. All data on growth measurements were evaluated by Student’s *t*-test analysis with the software SPSS 21.0 (SPSS Inc., Chicago, USA). Column bar and box and whiskers plots were generated using GraphPad Prism (v. 7.04).

### Measurement of mineral elements, endogenous phytohormones, soluble sugar, and starch contents

Three pools of leaves collected from 30 grafted plants at five phases were used for endogenous phytohormone and mineral element determination. The samples for mineral elements were placed into a forced air oven at 105°C for 30 minutes, and then at 75°C until a constant weight was reached to detect the dry weight. All the dried samples were ground into fine powder. Then, 0.50 g of each sample was dry-ashed in a muffle furnace at 200°C for 1 hour, 300°C for 1 hour, and 500°C for 8 hours, followed by dissolution in 10 ml 0.1 N HCl. The mineral elements were determined using an atomic absorption spectrometer (AA900T; PerkinElmer, USA). The samples for phytohormones were stored at −80°C. The levels of ABA, IAA, and GA_3_ were determined by HPLC, and the extraction method was developed and validated by He *et al*. [9], using the same equipment and conditions. JA and SA were determined using enzyme-linked immunosorbent assay (ELISA, Phytohormone Research Institute, Nanjing Agricultural University, China), following the manufacturer’s recommendations. The contents of soluble sugar and starch were measured using a Plant Soluble Sugar Content Assay Kit (BC0030, Solarbio, China) and a Starch Content Assay Kit (BC0700, Solarbio, China) according to the manufacturer’s protocols.

### Anatomical observation

Samples were fixed in 2.5% glutaraldehyde in 0.03 M phosphate buffer for 24 hours, then dehydrated in an ethanol series (15, 30, 50, 70, and 95%) for 90 minutes each. The method of sample preparation was developed and validated by Deng *et al*. [23]. The basic structure was determined using the paraffin section method and starch grains were dyed by I-KI staining, and observed by using a Zeiss Photomicroscope II (Carl Zeiss Jena, Germany). Phloem plugging was detected using toluidine blue and epifluorescence photomicrographs that were captured using an Olympus BX51 epifluorescence microscope (Olympus Inc., Tokyo, Japan).

### RNA-seq, data processing, and expression analysis

Total RNA was extracted as described previously [9]. Nine samples of the leaves of each compatible and incompatible graft were collected, and three biological replicates were collected for each sample. A total of 27 transcriptomes were obtained by RNA-seq using the Illumina Hiseq™ 4000 platform at Novogene Biotech Co., Ltd (Beijing). Before sequence assembly, the adapter sequences and low-quality reads were removed from the raw data. TopHat2 [52] was used to map clean reads to the reference genome of *C. maxima* [53]. The number of fragments per kilobase of transcript per million mapped reads (FPKM) was calculated with the RSEM tool [54]. The average FPKM values of the three replicates were calculated as the expression level of genes in each sample. To reduce the effects of transcriptional noise, genes with FPKM <.5 were considered not expressed [55, 56]. The sets of DEGs were identified using the eBays function in the limma package with *q* value <.1 and |log2 (fold-change)| >1.0.

The expression modules were generated by the Short Time-series Expression Miner (v. 1.3.12) [57] based on the FPKM values. The WGCNA algorithm [22] was applied to evaluate gene expression. The flashClust toolkit [58] (R language) was used to perform cluster analysis on samples and set appropriate thresholds. Heatmaps were created with the heatmap2 function in the R environment.

## Acknowledgements

This work was financially supported by Sichuan Science and Technology Program (2019YFH0061, 2020ZHCG0027, 2019NZZJ0015).

## Author contributions

X.R.W. conceived the idea; X.R.W. and W.H. designed the experiments; W.H. and R.X. performed the experiments, W.H. and Q.C. performed the data analysis, H.W., S.F.Y., Y.L., and Y.Z. provided the experimental materials; W.H. and Y.W. wrote the original manuscript; H.R.T., F.G.G., and X.R.W. critically revised the manuscript. All authors approved the final manuscript.

## Data availability

The RNA-seq datasets reported in our work have been submitted to the NCBI with the accession number of PRJNA704217.

## Conflict of interest

The authors declare that they have no conflicts of interest.

## Supplementary data

Supplementary data is available at *Horticulture Research* online.

## Supplementary Material

Web_Material_uhab072
