# Supplementary material for: Comparative transcriptomic analysis on compatible/incompatible grafts in Citrus
Source: Hortic Res. 2022 Jan 19;9:uhab072. doi: 10.1093/hr/uhab072 (PMC8931943; doi:10.1093/hr/uhab072)
Supplement: Web_Material_uhab072 [file web_material_uhab072.zip › Figures and Supplementary Figures.pdf]

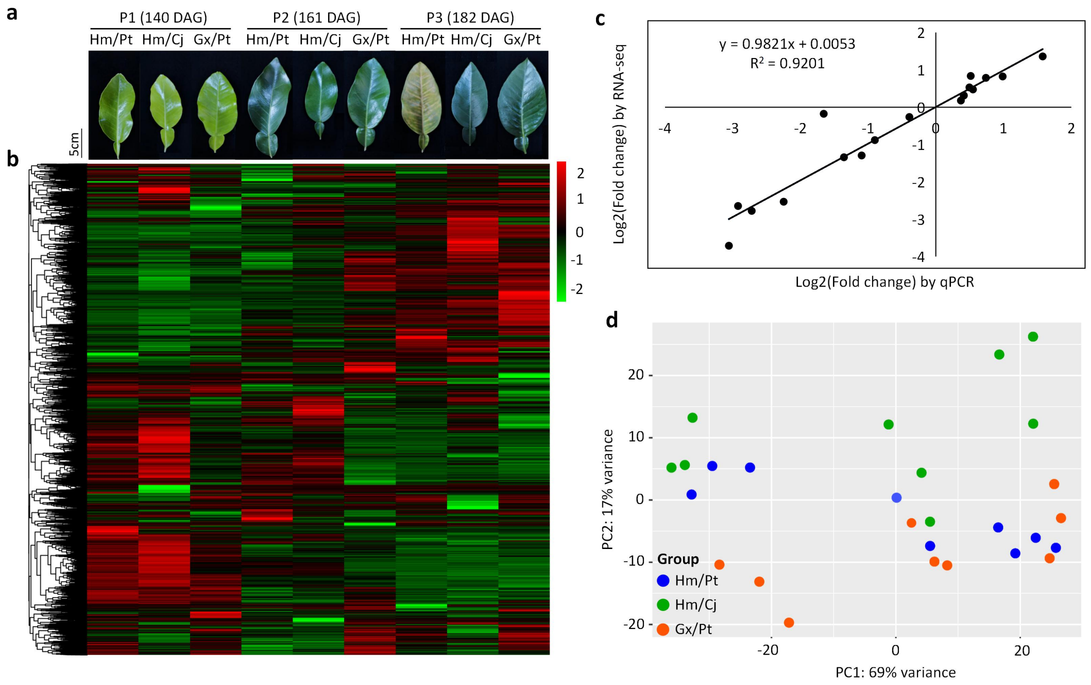

Fig. 1 Transcriptome analysis of leaves. **a** Leaf samples at three phases (P1, P2 and P3). **b** Hierarchical clustering of unigene expression. **c** Correlation of expression changes observed by RNA-seq (Y-axis) and qPCR (X-axis). **d** Principal component analysis (PCA) of the samples sequenced by RNA-seq. X-axis and Y-axis represent the first and second component. Dots with the same color indicate the same graft combination. Hm/Pt means 'Hongmian miyou' grafted onto trifoliate orange; Hm/Cj means 'Hongmian miyou' grafted onto *C. junos* cv. Xiangcheng; Gx/Pt means 'Guanxi miyou' grafted onto trifoliate orange. The same as below.

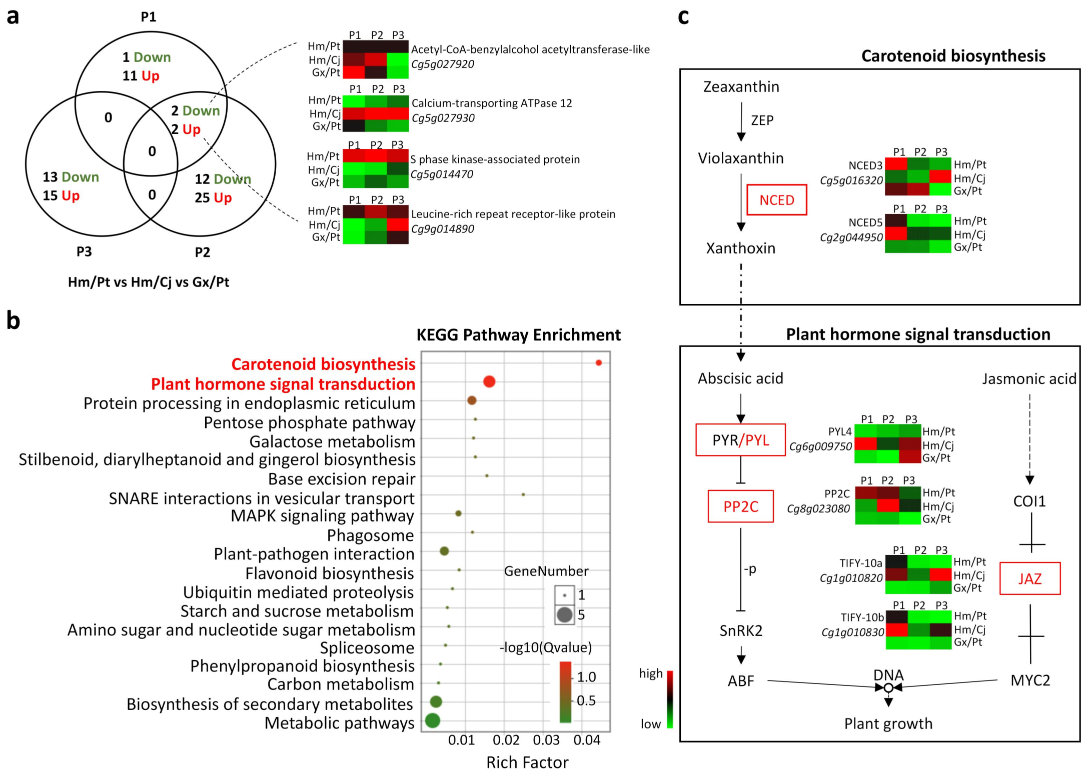

Fig. 2 Differentially expressed gene analyses. **a** Venn diagrams of genes differentially expressed between the Hm/Pt and controls at the same developmental stage. **b** KEGG enrichment analysis of the DEGs. **c** Expression of DEGs in carotenoid biosynthesis and plant hormone signal transduction pathways. Heatmap color indicates FPKM value.

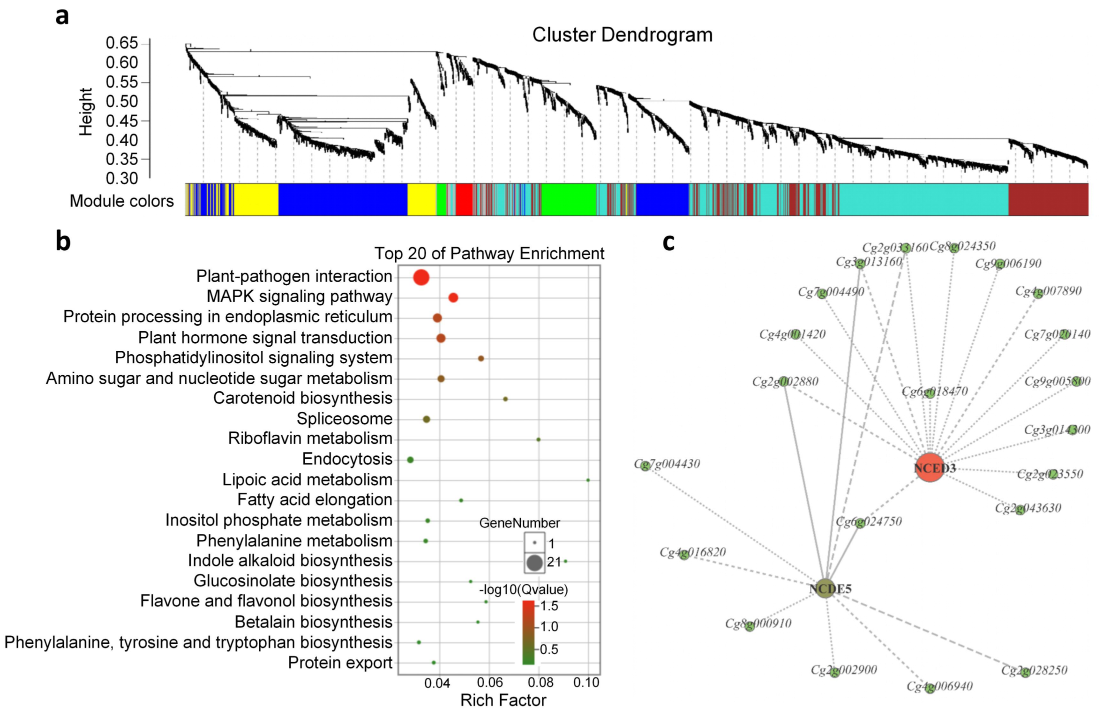

Fig. 3. WGCNA of differentially expressed genes. **a** Hierarchical cluster tree showing co-expression modules identified by WGCNA. Each leaf in the tree is one gene. The major tree branches constitute seven modules labelled by different colors. **b** KEGG enrichment analysis of the genes in the blue module. **c** Genes whose expression was highly correlated in the blue module.

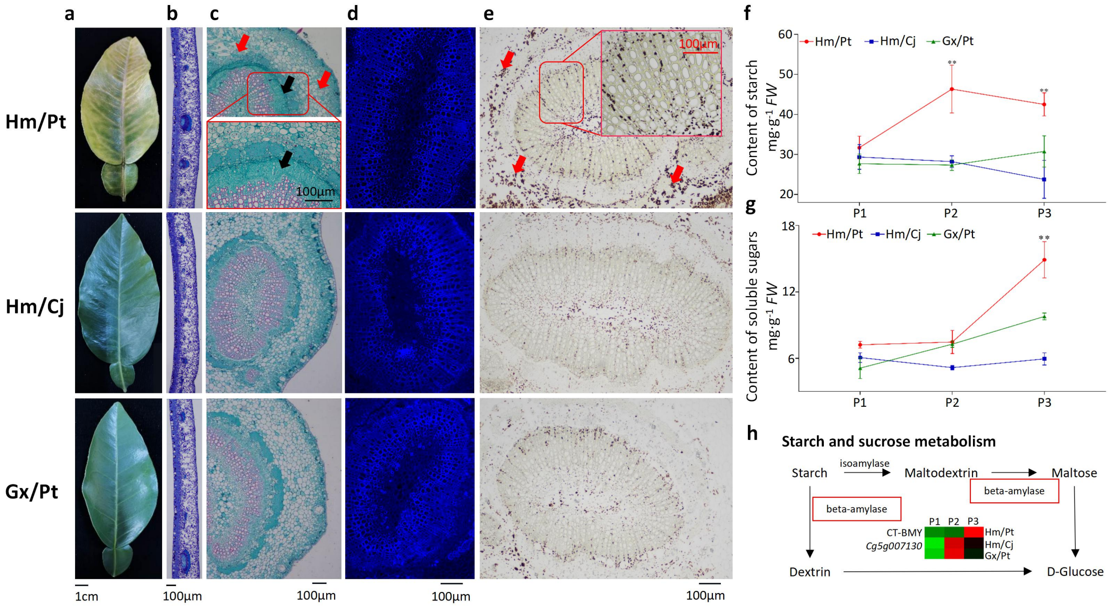

Fig. 4 Transverse section showing changes of leaves of compatible / incompatible grafts. a Leaf samples at P3. **b** Cross section of leaf. **c** Midrib section was observed and photographs taken under light microscope. **d** Epifluorescence photomicrographs of phloem. **e** Starch grains were dyed blue. **f-g** The contents of starch and soluble sugar. **h** Transcript abundance changes of starch and sucrose metabolism pathways. Asterisks represent significant differences compared to the control (**p* < 0.05, ***p* < 0.01), analyzed using Student's t-test. Heatmap shows the $\log_{10}$ (FPKM+0.01) of selected differentially expressed transcripts. The black arrows indicate the parenchyma cells and red arrows indicate starch accumulation, in **c** and **e**.

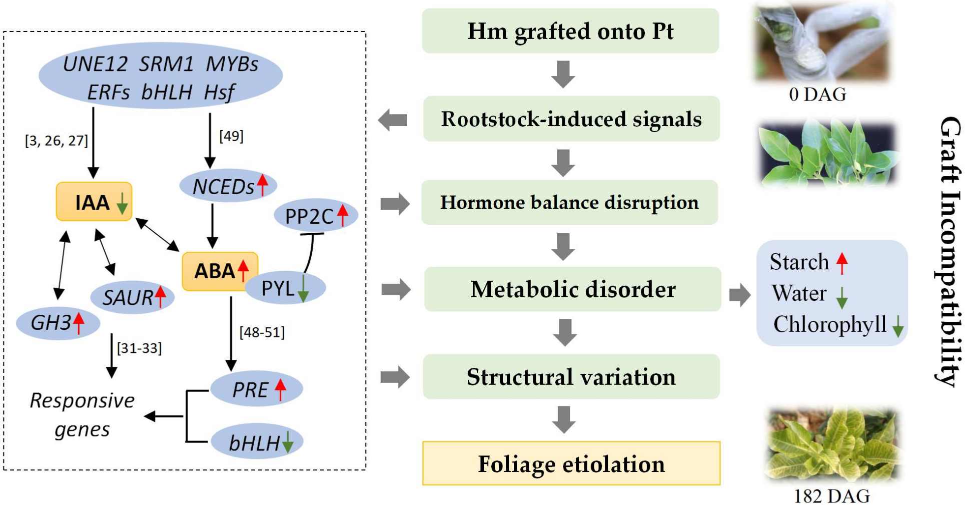

Fig. 5 Working model for the graft incompatibility in Hm/Pt. The rootstock-scion interaction induced signals to cause transport factors (TFs) genes activity. Differential expression of TFs could directly affect auxin and abscisic acid (ABA) signals transduction. Genes related to auxin and ABA were differentially expressed, causing a decrease in IAA and an increase in ABA level, further acclimation to grafting-induced stress, such as water deficiency, starch accumulation and chlorophyll decreased. Metabolic disorder caused structural variation, and lead to the foliage etiolation.

Note: The red and green arrows indicate up and down regulation respectively. Brackets [] contain the references number.

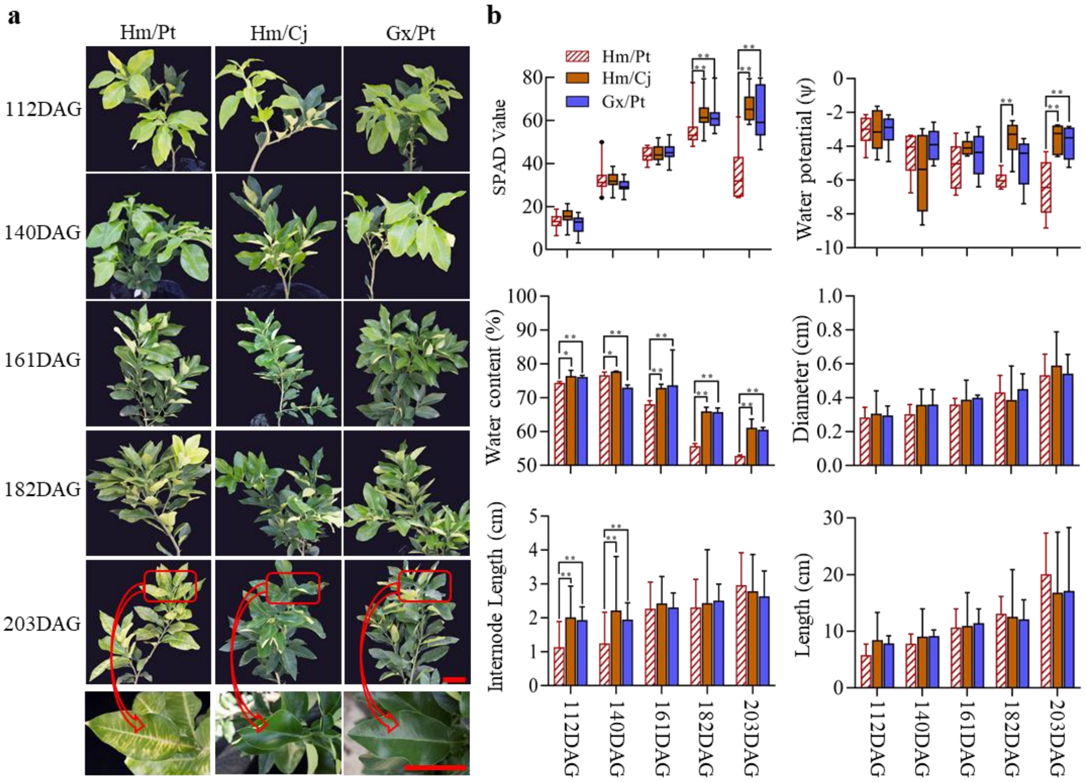

Figure S1. Compatible / incompatible grafts growth and sampling strategy. a Morphological difference between 'Hongmian miyou' (*Citrus maxima* (Burm.) Merrill, Hm) grafted onto trifoliate orange (*Poncirus trifoliate* (L.) Raf, Pt) (abbreviated Hm/Pt), Hm grafted onto Xiangcheng (*C. junos* (Sieb.) Tanaka, Cj) (abbreviated Hm/Cj) and 'Guanxi miyou' (Gx) grafted onto Pt (abbreviated Gx/Pt) at 112 DAG (days after grafting), 140 DAG, 161 DAG, 182 DAG and 203 DAG. Scale bars = 10 cm. **b** SPAD value, water potential and water content of leaves from summer shoots, and diameter, internode length and length of summer shoots. The box indicates the 25th and 75th percentiles. A line across the box represents the median. Whiskers are depicted as the maximum and minimum values and black circles represent outliers. Columns with bars represent means ± SE ($n > 10$). Asterisks represent remarkable differences compared to control (*$p < 0.05$, **$p < 0.01$), analysed using Student's t-test.

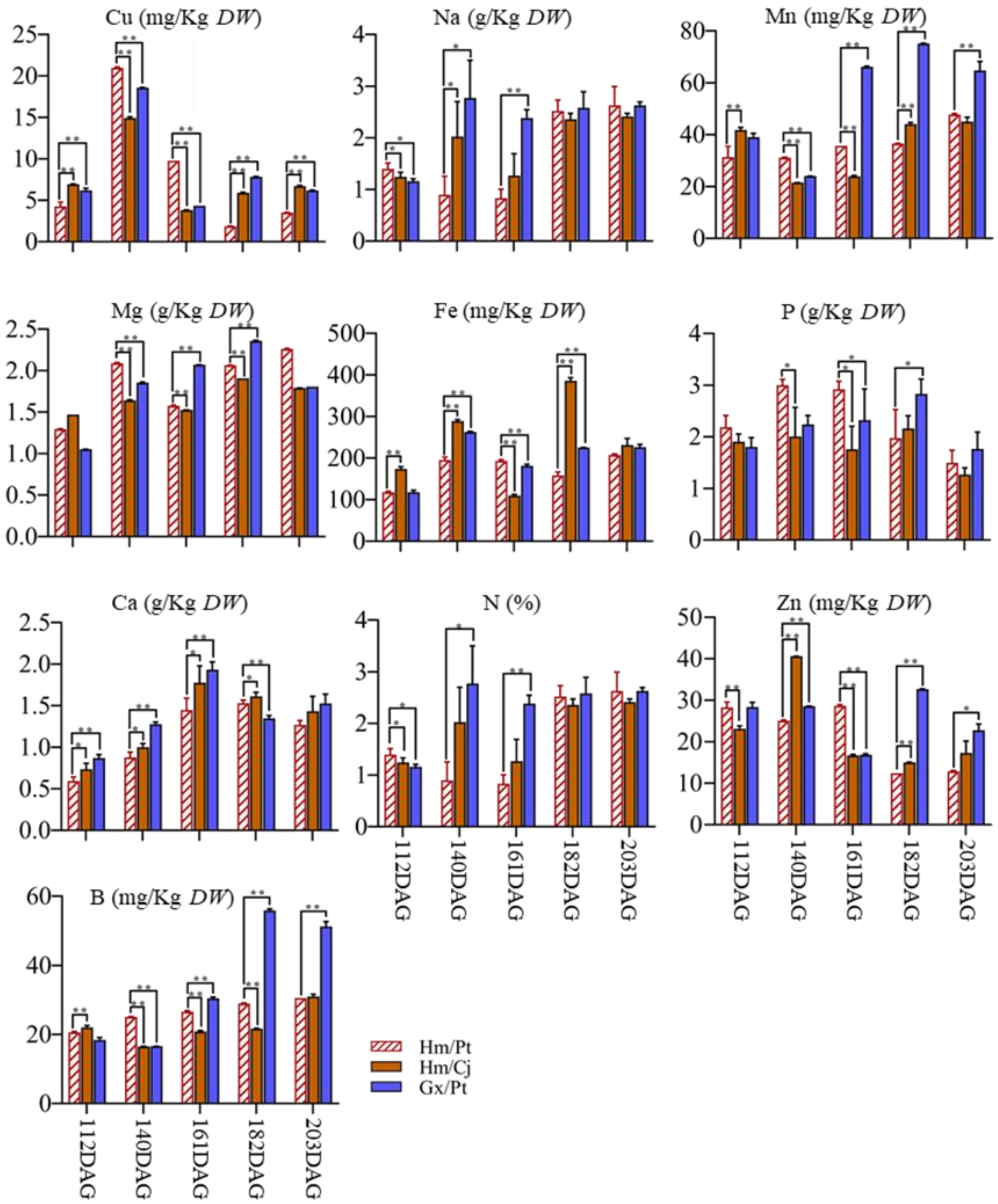

Figure S2. The trends of mineral elements levels in leaves. Columns with bars represent means ± SE ($n > 10$). Asterisks represent remarkable differences compared to the control (*$p < 0.05$, **$p < 0.01$), analysed using Student's t-test.

Note: Cu: copper, Na: natrium, Mn: manganese, Mg: magnesium, Fe: iron, P: phosphorus, Ca: calcium, N: nitrogen, Zn: zinc, B: boron.

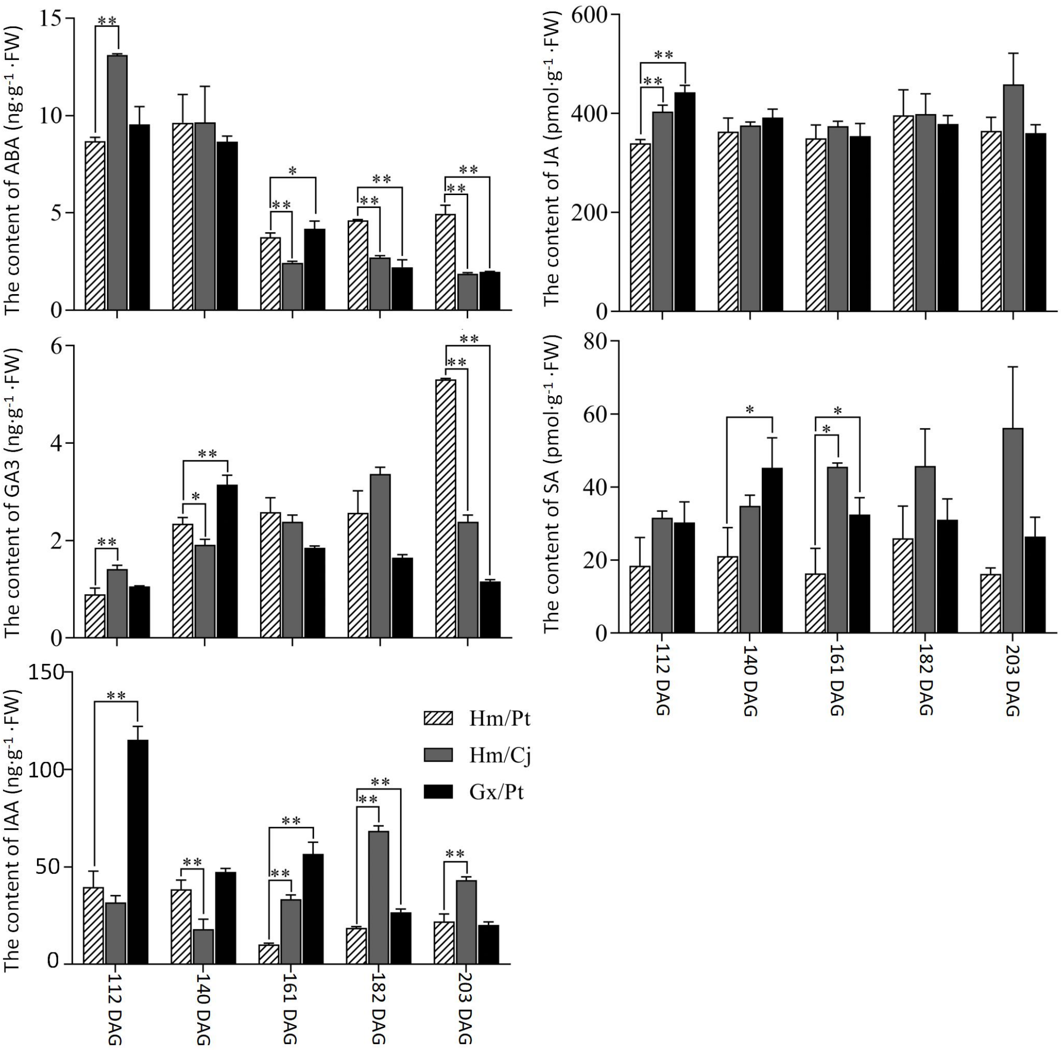

Figure S3. The trends of phytohormone levels in leaves. A single asterisk (*) represents statistically significant differences (p< 0.05), double asterisks (**) represent highly statistically significant differences (p<0.01), analysed using Student's t-test.

Note: ABA: abscisic acid, IAA: indole-3-acetic acid, GA_3: gibberellin A3, JA: jasmonic acid, SA: salicylic acid.

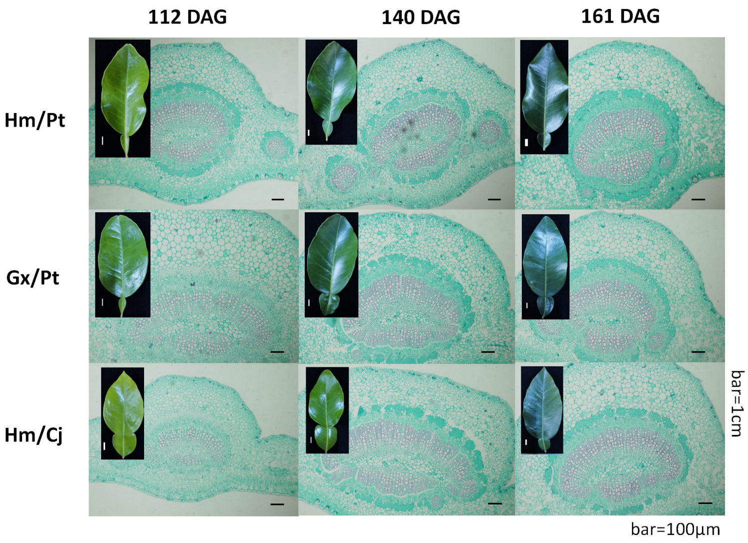

Figure S4. Transverse section of leaves. The bar in photomicrographs is 100 μm and the bar in photo of leaves is 1 cm.

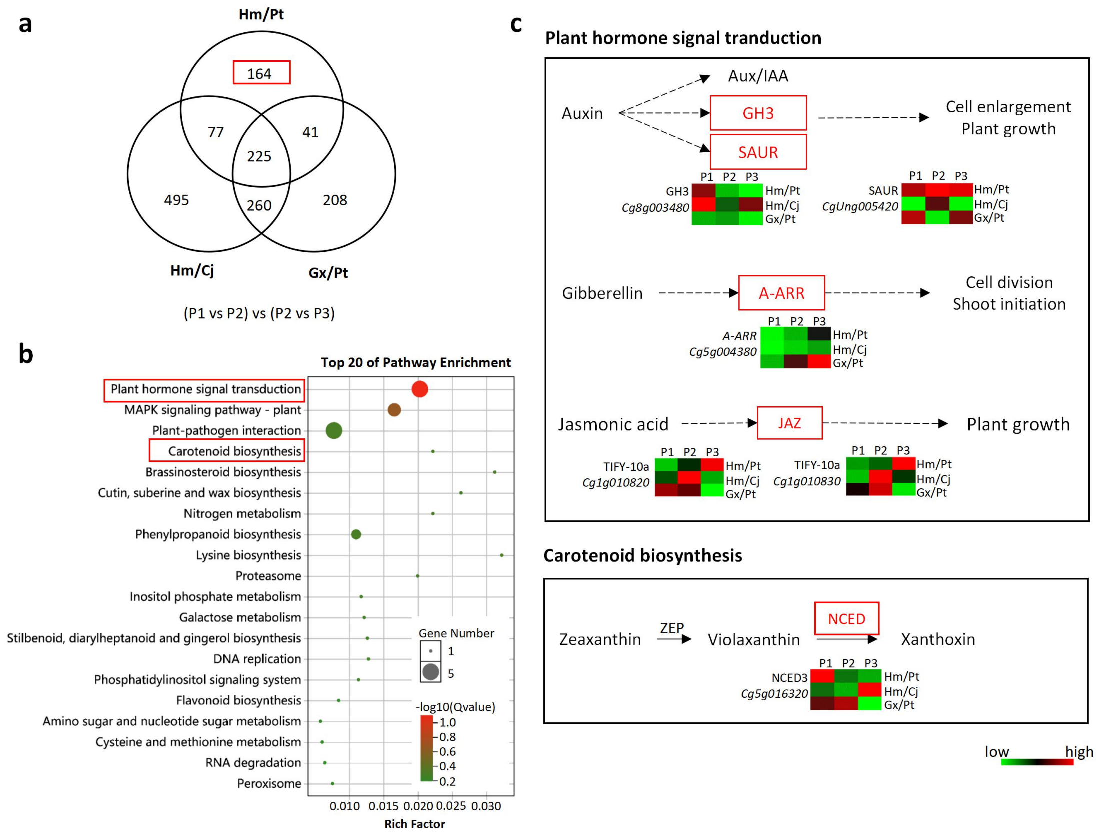

Figure S5. Analysis of DEGs from pairwise comparison between the different development stages at the same graft combination. a Venn diagrams of DEGs. **b** KEGG enrichment analysis of the DEGs. **c** Expression of DEGs in carotenoid biosynthesis and plant hormone signal transduction pathways. Heatmap color indicates FPKM value.

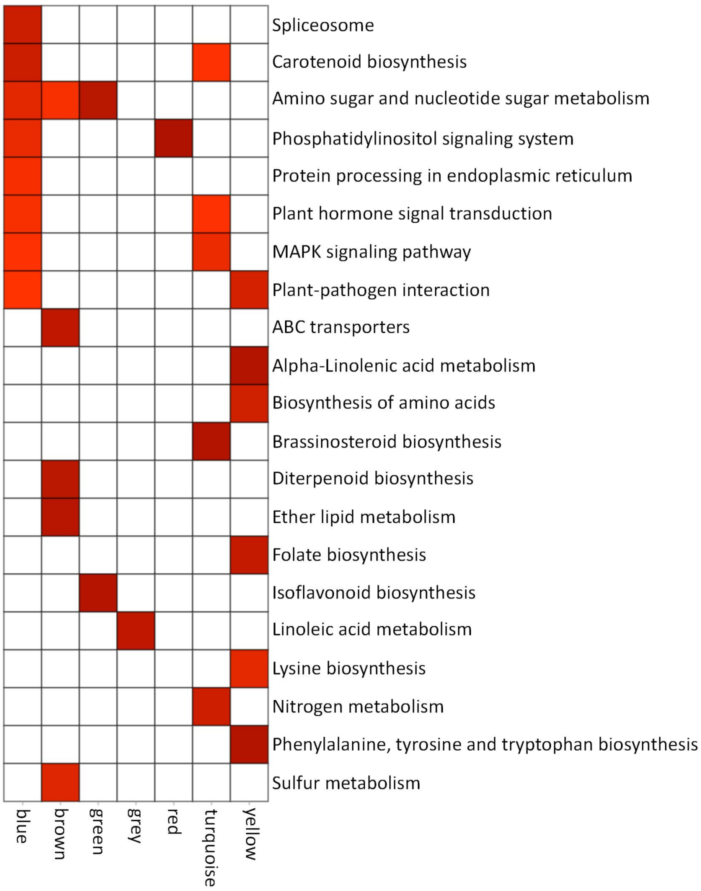

Figure S6. KEGG functional categories enriched by different co-expression modules. Only significant categories ($p < 0.05$) are displayed.

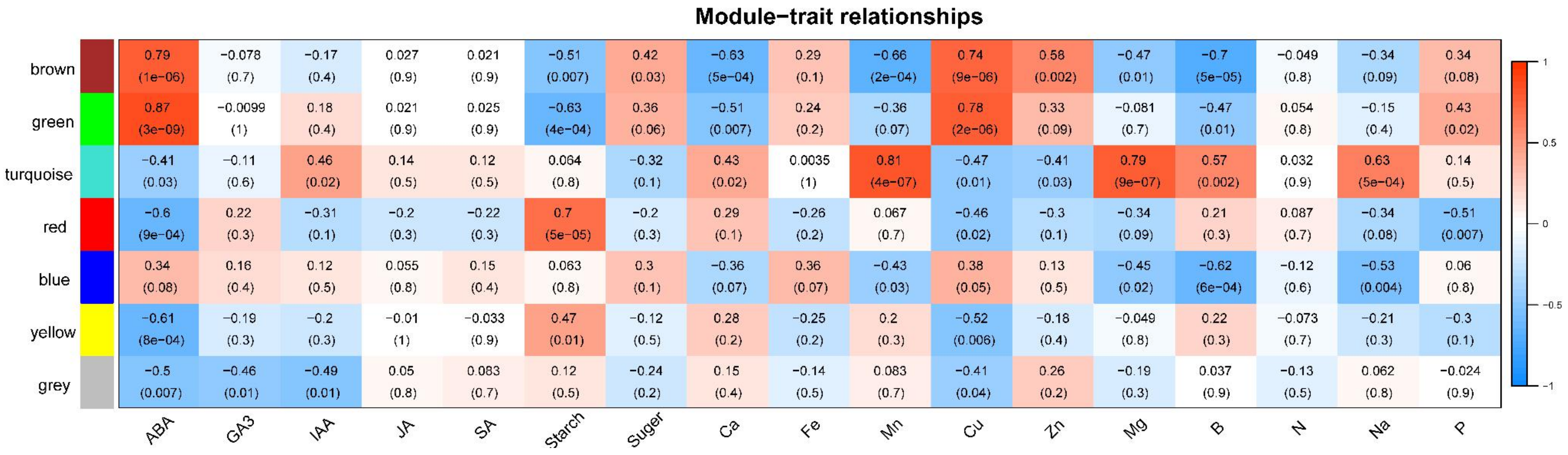

Figure S7. Relationships of modules and different traits. Each row in the table corresponds to a module, and each column corresponds to a trait.

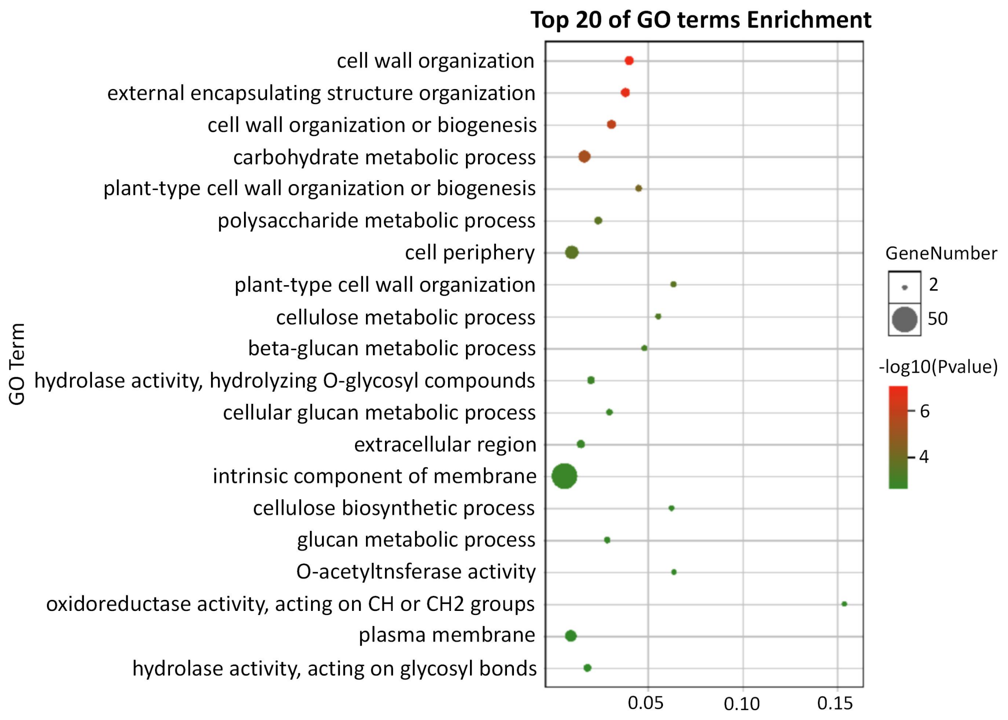

Figure S8. GO functional categories enriched by different co-expression genes in green module.

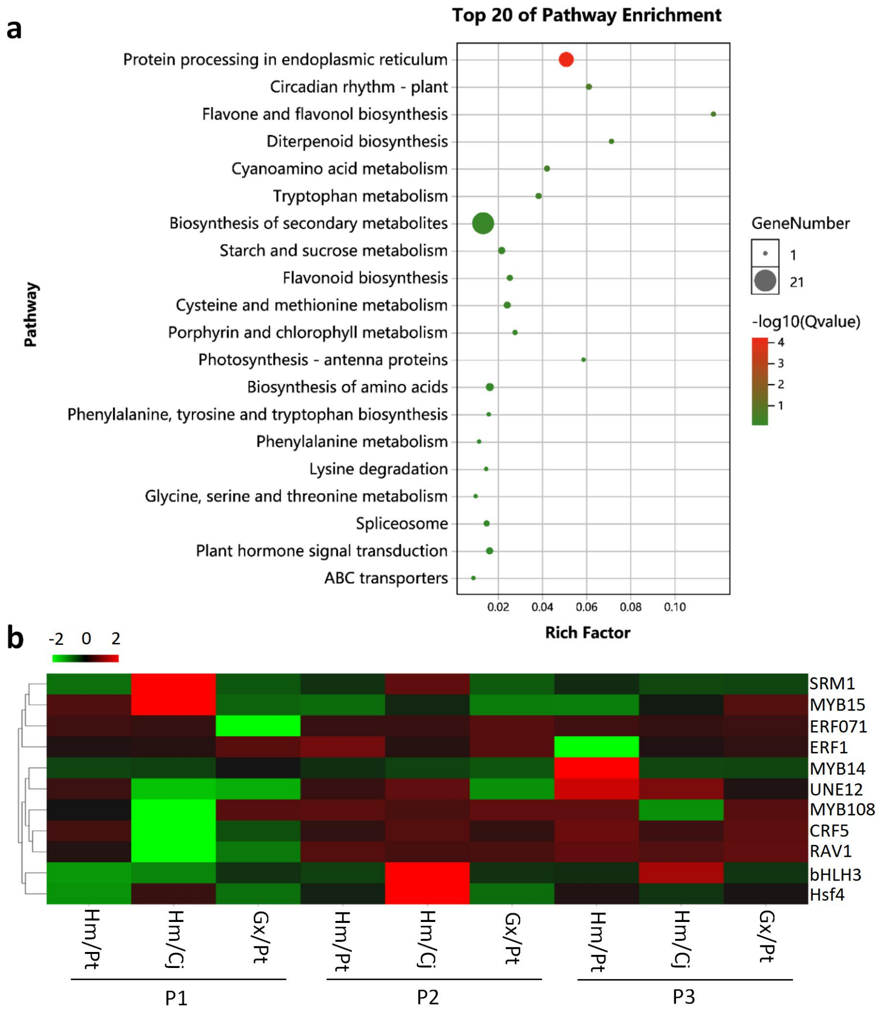

Figure S9. Analysis of DEGs from pairwise comparison between the scions from different rootstocks. a KEGG enrichment analysis of the DEGs. **b** Expression of transcription factors. Heatmap color indicates $log_{10}(FPKM+0.01)$ value.

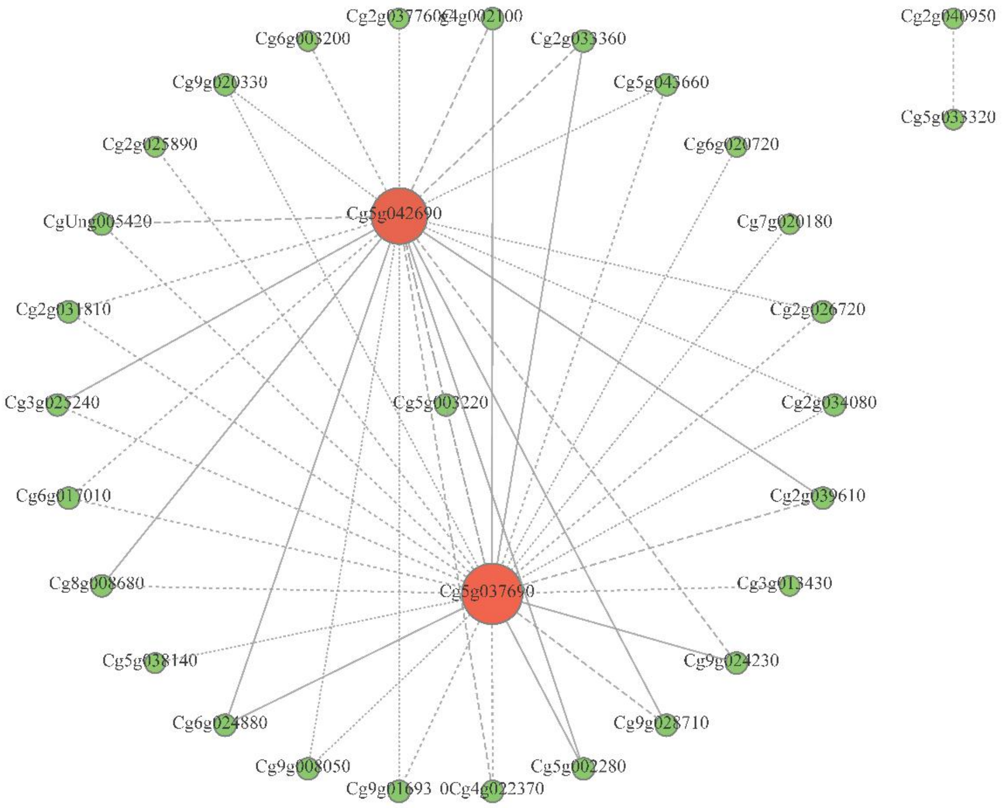

Figure S10. Hub genes selected from green module.
