# Supplementary material for: Comparative transcriptomic analysis on compatible/incompatible grafts in Citrus
Source: Hortic Res. 2022 Jan 19;9:uhab072. doi: 10.1093/hr/uhab072 (PMC8931943; doi:10.1093/hr/uhab072)
Supplement: Web_Material_uhab072 [file web_material_uhab072.zip › Table S1.pdf]

Table S1. Overview of RNA-seq data

Sample ID	Clean reads	clean bases (G)	Error rate(%)	Q20(%)	Q30(%)	GC content(%)	Alignment rate
Hm/Pt_P1_1	63907990	9.59	0.02	96.53	91.88	44.59	0.89
Hm/Pt_P1_2	64176800	9.63	0.02	96.63	92.08	44.37	0.88
Hm/Pt_P1_3	48490290	7.27	0.02	96.50	91.82	44.48	0.90
Hm/Pt_P2_1	66250118	9.94	0.02	96.66	92.15	43.96	0.88
Hm/Pt_P2_2	62949896	9.44	0.02	96.55	91.89	43.06	0.90
Hm/Pt_P2_3	51391904	7.71	0.02	96.85	92.51	44.03	0.90
Hm/Pt_P3_1	59282088	8.89	0.02	96.83	92.47	44.34	0.89
Hm/Pt_P3_2	63432256	9.51	0.02	96.71	92.23	44.42	0.89
Hm/Pt_P3_3	41743958	6.26	0.01	97.90	94.37	45.24	0.89
Hm/Cj_P1_1	49898318	7.48	0.02	96.55	91.96	44.15	0.89
Hm/Cj_P1_2	69835200	10.48	0.02	96.44	91.74	44.13	0.89
Hm/Cj_P1_3	54840660	8.23	0.02	96.60	92.03	44.40	0.89
Hm/Cj_P2_1	60020838	9.00	0.02	96.49	91.83	43.92	0.88
Hm/Cj_P2_2	45759350	6.86	0.01	97.74	94.09	44.61	0.90
Hm/Cj_P2_3	42723630	6.41	0.01	97.92	94.56	44.17	0.90
Hm/Cj_P3_1	51624938	7.74	0.02	96.58	91.98	44.37	0.89
Hm/Cj_P3_2	63158882	9.47	0.02	96.51	91.82	44.49	0.89
Hm/Cj_P3_3	53941876	8.09	0.02	96.87	92.52	44.17	0.89
Gx/Pt_P1_1	57178874	8.58	0.02	96.67	92.14	44.32	0.93
Gx/Pt_P1_2	62041226	9.31	0.02	96.91	92.57	45.12	0.94
Gx/Pt_P1_3	61052654	9.16	0.02	96.53	91.90	44.23	0.93
Gx/Pt_P2_1	53489360	8.02	0.02	96.78	92.36	44.36	0.93
Gx/Pt_P2_2	56173708	8.43	0.02	96.73	92.26	44.40	0.93
Gx/Pt_P2_3	53744886	8.06	0.02	96.70	92.19	44.33	0.93
Gx/Pt_P3_1	52038084	7.81	0.02	96.80	92.37	44.31	0.93
Gx/Pt_P3_2	50346832	7.55	0.02	96.95	92.67	44.27	0.93
Gx/Pt_P3_3	55367916	8.31	0.02	96.87	92.51	44.67	0.94

Note: Hm/Pt: *Citrus maxima (Burm.)* Merrill cv. Hongmian miyou grafted onto *Poncirus trifoliata* ; Hm/Cj: Hongmian miyou grafted onto *C. junos* ; Gx/Pt: Guanxi miyou grafted onto *P. trifoliata* . P1: 140 days after grafting (DAG); P2: 161 DAG; P3: 182 DAG. Named as Sample_DAG_repeat.
