# Supplementary material for: Comparative transcriptomic analysis on compatible/incompatible grafts in Citrus
Source: Hortic Res. 2022 Jan 19;9:uhab072. doi: 10.1093/hr/uhab072 (PMC8931943; doi:10.1093/hr/uhab072)
Supplement: Web_Material_uhab072 [file web_material_uhab072.zip › Table S2.pdf]

Table S2. Primer sequences used in qRT-PCR experiments.

**Unigene**	**Sequence**
Cg1g029290	F: CTCACTCTTCCTCCTCCTT
	R: CAATCATCGCTGTCATCCT
Cg5g023630	F: TCCGATGATTGAAGCATA
	R: AAGTAGTGGTTGAAGTGT
Cg4g015240	F: GCAACTACATAATTCCTAA
	R: CTAACATCAATCTCACTATT
Cg5g021980	F: AAGGAAGGAGAAGAAGGA
	R: TAACTTAGCAGCCGTAAC
Cg5g005560	F: TTGTTAGTCTTCGTTCTTAG
	R: TGATGGTGATGATGATGA
Cg6g009730	F: CCGCTATCTGCTGGTCAA
	R: AATCCTGCTTGTTACTGTCATTG
Cg6g024950	F: CAGTGATGTCGTGATGAG
	R: CCTGTTATTCCTTATGTGATTC
Cg4g001480	F: GTCATAATAATCAGGAAGAG
	R: ATACCTTGAAGTGAACAT
Cg7g014510	F: AATTCTGCCACAACTAAG
	R: CGTATAAGATTGCCAACT
Cg7g014500	F: AATTCTACCACAACTAAGG
	R: GGAGACATATAAGATTGCTA
β-Tubulin	F: ACATCCCGCCTAAGGGTCTG
	R: TTCCTCCGAAACATAGCCGTA
