# Supplementary material for: Comparative transcriptomic analysis on compatible/incompatible grafts in Citrus
Source: Hortic Res. 2022 Jan 19;9:uhab072. doi: 10.1093/hr/uhab072 (PMC8931943; doi:10.1093/hr/uhab072)
Supplement: Web_Material_uhab072 [file web_material_uhab072.zip › Table S3.pdf]

Table S3. Detail of differentially expressed genes in three pairwise comparisons

Pairwise comparison (Hm/Pt vs Hm/Cj vs	GeneID	P1			P2			P3			Description
		Hm/Pt	Hm/Cj	Gx/Pt	Hm/Pt	Hm/Cj	Gx/Pt	Hm/Pt	Hm/Cj	Gx/Pt	
P1	Cg5g027930	0.0830	2.6857	1.5252	0.0157	2.4736	0.2885	0.0250	0.0581	0.0180	calcium-transporting ATPase 12, plasma membrane-type-like
	Cg3g002190	2.4412	0.8880	0.5327	0.6021	2.1639	0.8570	0.9341	5.3200	7.0854	multicystatin-like isoform X1
	Cg9g014890	0.5277	0.1152	0.1247	0.6960	0.2401	0.3062	0.5672	0.8128	0.5123	probable leucine-rich repeat receptor-like protein kinase At1g35710
	Cg8g023080	13.2979	6.3927	5.4468	12.3014	16.0529	5.5778	8.1360	9.3002	4.0026	probable protein phosphatase 2C 24
	Cg9g012450	0.6651	0.3123	0.2220	0.1411	0.5843	0.3389	0.2736	0.5142	0.9151	probable galactinol--sucrose galactosyltransferase 2
	Cg3g015540	0.1368	0.5988	0.5412	0.2943	0.1994	0.3218	0.2202	0.5550	0.2193	aminotransferase ALD1-like
	Cg5g038850	9.8863	2.6431	2.7136	9.3340	6.3454	4.7949	14.7883	8.7922	7.7043	galactinol synthase 1
	CgUng002600	3.7891	0.7716	1.1693	2.8407	4.2132	1.6390	3.0696	8.7899	2.0571	RING-H2 finger protein ATL52-like
	Cg2g044460	1.4694	0.6787	0.6463	2.9694	1.8912	2.6312	4.0336	9.7121	5.9479	glucan endo-1,3-beta-D-glucosidase-like
	Cg7g016570	52.5473	22.6327	25.3540	42.1505	19.6326	20.7216	38.0615	16.4120	21.8056	No match
	Cg5g027920	0.0000	0.4367	0.1537	0.0000	0.5662	0.0828	0.0000	0.0000	0.0000	acetyl-CoA-benzylalcohol acetyltransferase-like
	Cg3g002200	21.8583	9.4378	5.9254	7.7514	16.9441	7.3554	9.9526	40.4451	62.1537	cysteine proteinase inhibitor 5-like
	Cg1g013020	16.6878	7.6716	7.0169	10.7379	7.6504	5.5827	11.2285	7.0871	5.9282	17.4 kDa class III heat shock protein
	Cg5g016320	73.0760	32.4938	23.1011	16.0330	29.3302	27.6383	6.8621	48.0417	4.9647	9-cis-epoxycarotenoid dioxygenase NCED3, chloroplastic
	Cg9g022790	14.4963	4.2664	4.3779	14.0693	21.8912	10.2839	9.7251	23.6412	7.7882	ethylene-responsive transcription factor 2-like
	Cg5g014470	1.1101	0.1315	0.2774	1.1742	0.1811	0.4817	1.0674	0.5223	0.3372	SKP1-like protein 1B
P2	Cg5g027930	0.0830	2.6857	1.5252	0.0157	2.4736	0.2885	0.0250	0.0581	0.0180	calcium-transporting ATPase 12, plasma membrane-type-like
	Cg9g026710	1.2663	2.8586	1.2184	2.0649	0.9889	0.5617	0.7855	0.7042	0.9634	25.3 kDa vesicle transport protein
	Cg9g029170	0.5847	0.9508	0.5707	0.7082	0.1824	0.1556	0.2307	0.6633	0.2395	zinc transporter 1-like
	Cg3g011330	0.1323	0.2415	0.2191	1.1688	0.2822	0.3967	1.0999	1.3565	1.0266	disease resistance protein At4g27190
	Cg3g012700	0.6319	1.0733	0.3887	3.7469	1.4922	1.5861	3.9849	3.0988	5.6335	LOB domain-containing protein 39-like
	Cg7g012330	0.4262	0.3254	0.2973	1.8745	0.7480	0.9459	3.0815	2.6230	2.7354	TLC domain-containing protein 4-like
	Cg5g008050	17.4664	54.6582	23.8610	1.4672	14.6591	5.6108	0.8195	1.1492	1.9845	inositol 2-dehydrogenase
	Cg9g014890	0.5277	0.1152	0.1247	0.6960	0.2401	0.3062	0.5672	0.8128	0.5123	probable leucine-rich repeat receptor-like protein kinase At1g35710
	Cg7g021890	0.4040	0.3710	0.8809	1.6416	0.4674	0.3022	1.0799	1.1202	0.9604	nodulin-related protein 1-like
	Cg3g025250	0.3334	0.4252	0.2812	0.6310	0.2576	0.2243	0.3537	0.7112	0.3879	U-box domain-containing protein 15
	Cg9g026720	0.2722	0.2557	0.1716	0.5824	0.1793	0.1662	0.1235	0.1810	0.2754	transcription factor MYB41-like
	Cg7g010530	0.1108	0.1322	0.1258	0.0382	0.1196	0.2117	0.1744	0.2018	0.2028	recQ-mediated genome instability protein 1
	CgUng006770	0.4583	0.1232	0.3588	0.0000	0.2285	0.1165	0.1531	1.1209	1.0301	methanol O-anthraniloyltransferase-like
	Cg5g001590	1.1289	0.9670	1.0946	0.6070	2.5211	2.0668	1.2815	1.9564	1.4763	probable GABA transporter 2
	Cg1g026580	30.7327	43.7074	37.7575	13.3137	26.3469	32.1322	14.4304	20.2950	16.0112	cellulose synthase A catalytic subunit 2 [UDP-forming]
	Cg2g018000	0.0000	0.0000	0.0000	1.1407	0.0000	0.0000	0.1309	0.0000	0.1478	hypothetical protein CUMW_084260
	Cg7g014410	0.1244	0.0472	0.0229	0.0000	0.0817	0.0252	0.0319	0.5349	0.0175	uncharacterized protein LOC102612994
	Cg1g017330	1.2066	1.2245	0.8464	1.8102	0.5629	0.9236	0.7061	0.6268	0.9923	No match
	Cg2g029220	0.0000	0.0935	0.0198	2.5441	0.0377	0.1716	0.0374	0.0859	0.0349	vacuolar iron transporter 1-like
	Cg9g019790	3.4398	4.2971	3.0308	5.0118	2.1778	1.8974	2.5232	1.2554	1.6395	GDP-L-galactose phosphorylase 2
	Cg2g044950	9.5917	16.2093	4.0559	1.4294	6.8007	3.9827	0.5861	6.2712	1.4079	probable 9-cis-epoxycarotenoid dioxygenase NCED5, chloroplastic
	Cg5g031580	4.6689	20.5214	9.3254	1.6523	8.1855	5.1008	1.5974	1.0952	2.9089	putative ankyrin repeat protein RF_0381
	Cg5g010840	26.4652	14.9918	24.9620	159.9428	63.6072	42.9540	76.1140	42.9349	42.8204	uncharacterized protein LOC102609761
	Cg6g003960	9.4191	8.2407	11.9842	25.3400	9.8829	9.0673	13.6142	19.3034	21.1423	WAT1-related protein At2g37460
	Cg5g027920	0.0000	0.4367	0.1537	0.0000	0.5662	0.0828	0.0000	0.0000	0.0000	acetyl-CoA-benzylalcohol acetyltransferase-like
	Cg4g003020	1.9333	0.9288	1.3588	8.6642	3.5026	2.7711	2.3444	2.6446	3.3772	Glucose-signaling factor like
	Cg6g016070	2.4485	2.4488	3.6173	1.8653	4.1801	6.9041	6.3961	14.6422	8.3389	ABC transporter G family member 34
	Cg1g024710	0.5492	0.2755	0.1837	3.6844	0.7387	1.0325	4.8184	4.5187	5.5093	probable LRR receptor-like serine/threonine-protein kinase At3g47570 isoform X2
	Cg7g016650	34.1806	37.1488	35.8619	39.6719	18.9892	18.6402	16.2934	15.2192	15.9107	myb-like protein X
	Cg1g001340	0.1253	0.0270	0.1236	9.4381	4.0672	3.8648	14.3739	7.8230	11.9176	GDSL esterase/lipase EXL3-like
	Cg3g003890	1.1911	1.9784	1.5461	4.8414	0.9611	2.1651	2.9654	2.0826	3.1491	uracil-DNA glycosylase
	Cg7g020820	0.4189	4.1093	1.2092	0.0950	1.4366	0.3790	0.0500	0.0803	0.0261	DEAD-box ATP-dependent RNA helicase 42
	Cg7g012890	0.2997	0.8905	0.4172	0.1274	0.4575	0.6276	0.4076	0.5043	0.5025	disease resistance protein SUMM2
	Cg8g004150	0.5387	0.6339	0.6014	0.7290	0.2079	0.3016	0.4208	0.1770	0.2054	scopoletin glucosyltransferase-like
	Cg9g009120	0.2237	0.2256	0.1415	0.5140	0.1865	0.2165	0.3587	0.8532	0.5139	protein NRT1/ PTR FAMILY 5.2
	Cg6g019240	374.3542	158.0826	318.9373	3533.9396	1543.3097	1252.9927	3209.4225	2406.8949	2273.9076	non-specific lipid-transfer protein 2-like
	Cg5g014470	1.1101	0.1315	0.2774	1.1742	0.1811	0.4817	1.0674	0.5223	0.3372	SKP1-like protein 1B
	CgUng015370	0.6096	0.0468	1.0320	7.6862	1.5050	1.8478	5.5065	2.9219	2.7188	probable disease resistance protein At1g15890
	Cg5g008450	0.1359	1.0002	0.2037	0.0255	0.3573	0.2676	0.2671	0.3548	0.7073	14 kDa proline-rich protein DC2.15-like
	Cg7g002360	0.0636	0.0996	0.0616	0.6069	0.1859	0.0956	0.8208	0.8248	0.4998	sister chromatid cohesion 1 protein 1
	Cg5g007130	0.5527	0.4887	0.5058	0.4910	1.4807	1.1929	1.4485	3.3674	2.1522	beta-amylase 3, chloroplastic
P3	Cg5g005560	4.7874	3.4115	4.7846	10.3541	5.1833	5.4138	7.2620	3.1723	3.5058	U-box domain-containing protein 33-like
	Cg2g011510	1.0360	0.5626	0.6193	4.7938	2.4375	2.0119	11.4849	3.3579	2.0366	Cytochrome C1 family isoform 1
	Cg1g010820	61.3611	88.3192	7.0827	7.4775	35.7269	7.8920	2.8397	122.4030	25.4419	protein TIFY 10A-like
	Cg1g005440	27.3877	18.1651	3.0225	3.4530	23.2966	9.5155	3.9150	95.0408	44.4836	mitogen-activated protein kinase kinase kinase 18-like
	Cg5g013330	2.8208	3.0187	2.5166	3.9844	3.4069	1.8326	1.2989	2.7065	3.1248	hydrophobic protein RCI2B
	Cg2g006520	35.2911	24.3185	38.2115	28.3830	34.1036	31.9634	9.2756	3.8185	3.1452	GDSL esterase/lipase At3g27950
	Cg6g009730	3.0298	7.3039	2.3035	10.1404	10.9051	7.0087	4.9870	28.4116	15.1459	mitoferrin-like
	Cg6g009720	23.2798	43.0498	27.9002	457.6763	24.4969	130.1192	15.0002	108.9789	60.1508	ferritin-3, chloroplastic
	Cg5g002310	3.7614	5.2959	3.1450	4.1611	2.2738	2.2753	2.5633	0.9930	0.9269	hypothetical protein CUMW_042270
	Cg4g020410	0.3073	0.0834	0.4251	0.4086	0.4548	1.3245	0.2753	1.0365	0.7656	cycloartenol synthase-like
	Cg5g006140	31.7359	36.0585	13.3102	12.0413	26.6083	20.9620	10.9289	67.2905	78.6970	BTB/POZ and TAZ domain-containing protein 1-like
	Cg1g010830	59.1834	110.8269	5.7100	4.3236	27.0123	4.1698	1.3002	63.9833	12.4715	protein TIFY 10A-like
	Cg2g004750	0.0727	0.0254	0.0098	0.0197	0.1568	0.0876	0.0000	0.7917	0.6613	methanol O-anthraniloyltransferase-like
	Cg6g004050	1.3501	1.7799	0.8390	0.4685	0.8354	0.4111	0.3954	0.0225	0.0521	RING-H2 finger protein ATL51-like
	Cg7g017070	0.0167	0.1008	0.0643	0.5853	0.3400	0.3016	0.4937	1.3765	1.1299	cytochrome P450 78A5-like
	Cg9g002330	3.7480	3.8601	4.1081	0.5789	1.1664	0.4422	0.6665	2.1212	2.2801	hypothetical protein CICLE_v10006300mg
	Cg5g003220	27.1023	21.4606	34.3081	14.0112	8.5882	10.7490	3.3244	0.6047	0.6501	transcription factor PRE4-like
	Cg2g044900	11.1548	15.6124	9.2623	2.8786	3.9190	2.7614	0.8016	0.2467	0.2534	glucan endo-1,3-beta-glucosidase 14
	Cg7g021900	10.7087	17.6196	8.3284	6.7623	3.9826	2.6116	2.1488	1.0099	0.7208	hepatoma-derived growth factor-related protein 2 isoform X1
	Cg8g022740	1.3046	0.7471	0.7369	1.2374	2.2059	1.3210	0.4699	0.0786	0.0679	laccase-2 isoform X2
	Cg7g005100	2.4905	3.1089	2.6702	2.6869	1.5155	0.8252	1.1131	0.4664	0.3847	hypothetical protein CISIN_1g025471mg
	Cg6g020720	846.3833	698.8921	1028.9769	200.2936	212.9017	426.8724	68.9057	26.1479	25.0120	vegetative cell wall protein gp1-like isoform X2
	Cg5g033320	3.2140	2.7353	3.9209	2.6631	2.6261	1.5564	0.9506	0.3048	0.3210	hypothetical protein CISIN_1g047327mg
	Cg5g002320	3.5007	5.6127	2.5111	2.8183	1.2946	1.2849	0.8691	0.3805	0.3218	Nuclear transport factor 2 family protein, putative
	Cg2g040090	14.9061	22.2081	12.8683	13.0378	17.4077	11.0218	11.4883	28.3666	22.9364	15.4 kDa class V heat shock protein
	Cg4g001480	34.2452	13.4617	45.0897	41.1297	36.0312	58.5505	39.6595	17.0669	18.6178	CBL-interacting serine/threonine-protein kinase 25-like
	Cg7g000360	9.1557	5.8804	13.3645	9.3123	6.2507	7.8784	4.9800	1.9407	2.2030	UDP-glycosyltransferase 76F1-like
	Cg6g009750	35.3367	112.1939	35.3859	40.1314	62.1684	30.4317	46.1831	91.4248	99.1054	abscisic acid receptor PYL4
