# Supplementary material for: Comparative transcriptomic analysis on compatible/incompatible grafts in Citrus
Source: Hortic Res. 2022 Jan 19;9:uhab072. doi: 10.1093/hr/uhab072 (PMC8931943; doi:10.1093/hr/uhab072)
Supplement: Web_Material_uhab072 [file web_material_uhab072.zip › Table S4.pdf]

Table S4. The number of genes in seven constructed modules

Module Cloros	Gene Number
blue	476
brown	399
green	143
grey	50
red	38
turquoise	720
yellow	202
