# Supplementary material for: Comparative transcriptomic analysis on compatible/incompatible grafts in Citrus
Source: Hortic Res. 2022 Jan 19;9:uhab072. doi: 10.1093/hr/uhab072 (PMC8931943; doi:10.1093/hr/uhab072)
Supplement: Web_Material_uhab072 [file web_material_uhab072.zip › Table S5.pdf]

Table S5. Detail of genes in blue module

GeneID	P1			P2			P3			Description
	Hm/Pt	Hm/Cj	Gx/Pt	Hm/Pt	Hm/Cj	Gx/Pt	Hm/Pt	Hm/Cj	Gx/Pt	
CgUng006760	6.7927	10.3029	2.3988	0.5255	4.8147	0.4463	0.2714	6.3865	3.9929	methanol O-anthraniloyltransferase-like
CgUng004370	2.2160	2.2338	2.1505	2.1570	1.6815	2.2449	3.7724	1.5884	3.3463	pentatricopeptide repeat-containing protein At5g66520-like
CgUng002190	27.5866	26.1111	39.3006	31.9791	25.5105	43.2264	26.2298	11.6482	44.2620	transcription factor MYBS1
CgUng001520	179.7968	284.7487	38.6323	73.8026	135.8649	62.6353	32.7154	519.4522	51.8600	protein ALP1-like
CgUng001200	7.9346	7.3763	6.7327	13.4785	7.4113	6.5890	9.7960	4.3091	6.7135	No match
CgUng000890	30.0719	46.6504	13.6293	15.9400	21.1806	11.3787	9.8374	34.1548	12.5545	PLATZ transcription factor family protein
CgUng000770	74.3505	89.1424	25.0024	36.4353	73.9603	38.4962	37.9123	195.2329	52.5551	transcription factor MYC2-like
Cg9g027680	0.7520	0.2056	4.4010	2.3743	0.6778	5.1713	5.6030	0.8573	11.2564	probable WRKY transcription factor 27
Cg9g027580	3.0899	5.8295	1.1733	1.6295	3.1058	0.8096	0.9468	7.0336	0.9045	heavy metal-associated isoprenylated plant protein 39-like
Cg9g027560	114.6422	193.3059	38.4323	72.7791	108.8663	33.6758	25.3557	199.8866	24.0830	heavy metal-associated isoprenylated plant protein 39
Cg9g026260	64.8604	56.3811	2.0682	11.5884	16.5843	5.2779	0.9480	89.9788	4.3456	CRT/DRE binding factor
Cg9g026010	14.4175	7.5544	20.1951	20.8528	14.5304	40.7322	20.3416	12.1474	46.7864	gibberellin 20 oxidase 1
Cg9g025700	32.3771	37.4078	23.0517	24.8401	35.9668	21.7623	18.0647	42.8118	14.0844	xyloglucan galactosyltransferase XLT2
Cg9g025090	1.2984	0.8791	1.5436	1.2607	0.7145	2.4305	1.6983	0.9506	2.3787	pentatricopeptide repeat-containing protein At5g62370
Cg9g024530	3.0636	3.2113	2.5343	1.9642	4.5589	1.5915	1.4458	10.7638	0.7769	L-type lectin-domain containing receptor kinase IX.1-like
Cg9g023970	236.2048	108.6616	159.3942	234.9481	67.8467	140.3099	214.5774	61.9345	195.8128	heat shock 22 kDa protein, mitochondrial isoform X2
Cg9g023940	23.0632	28.3507	13.7778	17.5205	14.2628	14.5413	11.2487	22.8821	11.6137	acidic endochitinase-like
Cg9g023040	2.1415	1.4761	2.2306	1.9675	3.2394	3.9949	1.0767	0.9508	2.2452	berberine bridge enzyme-like 13
Cg9g022830	141.8817	204.8309	36.9696	110.4809	141.0091	59.2638	43.0534	286.7917	80.1589	ethylene-responsive transcription factor ERF105-like
Cg9g022820	99.7811	106.2529	23.4555	119.7790	138.5700	49.2221	43.9288	268.9400	57.3849	ethylene-responsive transcription factor ERF105-like
Cg9g022160	5.0868	13.1119	1.1452	1.4249	4.7838	0.7742	1.0231	4.3901	1.2456	Arabinogalactan protein 40 precursor
Cg9g021680	13.9985	14.8374	10.4695	11.0555	13.7329	11.3025	8.5124	18.1511	7.5892	calcium-dependent protein kinase 32-like
Cg9g021420	7.8820	19.4023	2.4868	5.7114	9.7375	2.7250	3.0259	24.3422	4.0474	hypothetical protein CICLE_v10006321mg
Cg9g019420	7.5228	7.5426	7.2289	3.8044	5.3283	4.2261	4.9524	12.2138	4.4989	proline-rich receptor-like protein kinase PERK1
Cg9g019350	9.4243	12.2056	8.1180	5.0781	7.5833	4.9420	4.4811	9.0328	5.5326	protein CfxQ homolog
Cg9g019170	81.0806	135.3571	12.9918	38.9459	72.0175	15.9760	9.0080	177.9146	13.7119	Arabinogalactan peptide 20
Cg9g018300	39.1442	18.6403	27.1656	88.8169	35.7028	56.3910	49.8294	22.1924	36.5577	glucan endo-1,3-beta-glucosidase, basic isoform-like
Cg9g017540	56.1825	0.1948	80.1239	29.6907	0.9675	54.5280	28.1860	0.2186	10.2556	probable F-box protein At5g04010
Cg9g017450	118.4676	79.8036	61.7231	65.0990	57.7184	44.3875	59.9466	115.1536	57.0019	hypothetical protein CICLE_v10006298mg
Cg9g014580	15.0125	7.9832	19.8112	14.2781	9.1365	16.0719	18.0393	7.2035	13.1783	anthocyanidin 3-O-glucosyltransferase
Cg9g014240	4.1455	3.3769	4.4273	4.8425	3.2276	4.5261	5.5639	2.7445	4.8569	70 kDa peptidyl-prolyl isomerase
Cg9g011430	2.2598	7.1044	1.7366	2.5976	5.8860	1.0366	1.8441	4.1754	1.0030	UPF0481 protein At3g47200-like
Cg9g011290	0.9257	1.6242	0.3857	1.3369	1.7610	0.5655	1.0185	2.7385	1.1205	UPF0481 protein At3g47200-like
Cg9g010260	34.8104	42.9718	25.0734	43.6750	85.4759	30.0626	31.5839	78.5121	26.9077	ammonium transporter 1 member 1
Cg9g007890	4.3665	4.5839	2.6524	2.5333	3.8019	2.6000	1.4596	4.3996	2.0118	RING-H2 finger protein ATL16-like
Cg9g007150	8.6958	14.8641	0.1871	0.6934	4.2841	0.6542	0.0945	14.1132	0.0925	transcription factor bHLH92
Cg9g006190	38.1050	58.2297	18.8388	25.5603	33.6376	15.1650	17.8628	31.4233	14.6483	E3 ubiquitin-protein ligase RNF128
Cg9g005800	15.8358	29.8948	9.7542	12.1376	16.3872	5.7911	7.3940	17.0489	5.2736	uncharacterized N-acetyltransferase p20-like
Cg9g005770	1.1745	3.3109	0.4785	0.7396	2.4857	0.3982	0.6777	6.9755	0.7314	uncharacterized N-acetyltransferase p20-like
Cg9g005340	1.8619	2.3834	1.3669	0.8220	1.3011	0.9358	0.6668	1.4906	0.8973	G-type lectin S-receptor-like serine/threonine-protein kinase LECRK3
Cg9g005300	18.0376	24.5556	5.1201	6.9810	12.9222	4.5787	3.6197	36.8231	4.5428	suppressor of disruption of TFIIS
Cg9g004560	8.4548	14.6219	8.3326	3.1009	6.2802	3.4945	2.9064	10.3596	1.5684	basic 7S globulin
Cg9g004190	3.5907	8.0776	0.5814	1.6287	3.9161	1.3810	0.4623	6.9942	0.7338	hypothetical protein CUMW_128320
Cg9g003900	631.4367	679.9163	410.2430	586.4852	630.4902	476.7737	439.4821	1104.0364	353.5229	hypothetical protein CUMW_020240
Cg9g003100	242.3766	417.1562	56.6816	178.4921	256.1118	70.7955	60.5672	539.8227	63.1019	protein SRC2-like
Cg9g003090	29.8405	41.0168	16.0072	26.9207	28.0111	13.6882	12.5421	70.3296	14.9686	60S ribosome subunit biogenesis protein NIP7 homolog
Cg9g002490	56.1762	98.5295	20.6694	23.7957	44.8080	18.0840	11.9275	96.5058	11.7345	RING-H2 finger protein ATL2
Cg9g002250	2.6187	1.5834	2.2517	1.4228	1.7170	0.2715	0.5604	3.8003	0.8016	hypothetical protein CICLE_v10006300mg
Cg9g001720	10.7282	13.9994	8.4091	4.5755	7.0413	5.6036	3.5547	9.0897	3.2615	COBRA-like protein 7
Cg9g001580	4.6909	2.1836	5.8159	9.3932	8.8808	13.0598	6.3697	5.2779	8.3979	beta-D-xylosidase 1-like
Cg9g001080	267.6067	299.4469	221.0779	205.3402	232.7902	191.6056	121.1083	514.7066	135.8261	calcium-binding protein CP1
Cg9g000750	14.7626	14.4510	7.7320	5.2250	10.2511	6.2414	5.6573	25.0664	6.8471	alkaline/neutral invertase A, mitochondrial
Cg8g024810	31.4809	36.8553	16.0218	16.8018	24.5731	14.8118	11.3406	49.3320	11.4385	exocyst complex component EXO70B1
Cg8g024350	64.9931	107.9505	20.0190	17.9010	54.2439	11.6973	6.1651	78.8193	7.3337	mitogen-activated protein kinase kinase kinase 18-like
Cg8g024340	29.6434	31.6409	3.2034	11.3049	19.7728	8.5511	4.1483	109.8201	8.3950	probable protein phosphatase 2C 25
Cg8g023160	2.8317	3.7030	1.1714	0.9051	1.9452	1.0730	0.6361	5.1539	0.4802	metal tolerance protein B isoform X1
Cg8g022770	114.0871	179.8432	40.8706	84.9972	131.0121	61.2093	45.8574	345.5769	51.4760	E3 ubiquitin-protein ligase RDUF2-like
Cg8g022180	12.6050	23.4393	8.0428	13.0155	16.3038	8.3158	8.6689	27.2190	10.7304	Transmembrane protein
Cg8g022020	0.2000	0.1161	0.9917	1.2333	0.5624	1.1955	1.1365	0.5562	1.3710	P-hydroxybenzoic acid efflux pump subunit aaeB
Cg8g021770	10.8071	16.3262	1.2732	3.3865	8.1024	2.3801	1.6521	41.4191	2.0017	zinc finger protein ZAT12
Cg8g021580	40.4504	101.2439	7.6951	8.0279	21.0398	3.9416	0.8482	45.9217	1.1175	exocyst complex component EXO70H1-like
Cg8g021550	39.6169	42.0115	15.8814	8.8847	19.1604	6.5281	4.8281	36.3629	9.9632	ankyrin repeat-containing protein At5g02620
Cg8g021210	5.8491	5.7637	4.3066	4.2273	3.4084	3.0981	3.0075	5.9025	2.5244	probable serine/threonine-protein kinase PBL5
Cg8g021130	3.0395	5.4209	1.6293	4.6550	3.5885	2.4419	2.9544	9.1739	2.8842	hypothetical protein CUMW_035120
Cg8g021010	38.6073	63.2183	9.6686	19.7246	34.7720	15.1166	10.8447	152.2640	13.9000	mitogen-activated protein kinase 3
Cg8g020870	2.1779	2.4911	1.4165	2.8420	2.6872	1.3338	1.8267	4.6425	1.3739	tRNA-splicing endonuclease subunit Sen2-1-like isoform X2
Cg8g020860	3.3912	3.0799	3.2499	1.9850	3.1210	2.6453	2.4854	4.9756	2.2293	phosphatidylinositol transfer protein 1-like isoform X1
Cg8g020330	1.2887	0.4268	3.8851	5.0532	2.1743	5.4313	4.3391	0.8387	3.8886	DBP1-interacting protein 2
Cg8g019570	98.4662	162.3808	46.8020	85.1829	107.2405	37.3119	50.8963	187.1129	40.6417	heavy metal-associated isoprenylated plant protein 3
Cg8g019360	8.0603	11.5145	5.3662	4.5983	5.8810	5.9743	4.3368	11.0366	6.8495	protein NUCLEAR FUSION DEFECTIVE 4
Cg8g018160	304.0781	344.5519	148.0883	159.0783	330.7874	128.7244	103.7265	576.1987	140.1298	histone deacetylase HDT1-like
Cg8g018110	67.8921	78.9500	36.2569	33.3595	51.0768	37.1336	25.4724	84.4729	27.5514	arogenate dehydratase/prephenate dehydratase 6, chloroplastic-like
Cg8g017790	116.2854	124.8412	4.8965	4.3155	49.2880	7.5565	0.6130	82.3009	3.4993	cytochrome P450 94C1
Cg8g017590	221.7492	336.8177	66.6845	160.2884	257.6611	83.6149	79.1609	512.5167	116.4195	zinc finger A20 and AN1 domain-containing stress-associated protein 3-like
Cg8g013730	8.5081	13.8263	3.3028	2.6705	7.4500	2.2620	2.0292	31.2914	3.0888	NAC domain-containing protein 90
Cg8g013270	1.6407	2.9409	0.8997	1.1585	1.6396	0.6765	0.6173	2.7948	0.4593	probable F-box protein At2g36090
Cg8g013130	464.1120	817.2427	110.4091	287.4400	457.9276	126.9238	104.8523	912.8422	124.3904	probable CCR4-associated factor 1 homolog 11
Cg8g012210	8.5031	15.2556	4.2987	11.4265	13.6406	5.3314	9.9864	21.1239	9.0973	pre-mRNA-processing protein 40A-like
Cg8g008930	5.8666	2.6649	7.3715	5.3069	2.2400	9.7036	6.5389	2.7989	10.8069	tRNA-dihydrouridine(47) synthase [NAD(P)(+)]-like
Cg8g008840	261.4944	208.3127	197.3934	178.1581	181.6355	199.1063	141.7195	361.3531	144.7129	arginine decarboxylase
Cg8g006420	0.9055	0.3429	8.5736	3.8550	0.6601	10.0250	8.1454	0.5037	25.5351	Organ specific protein
Cg8g006070	30.5793	38.4176	17.4124	34.1207	46.6039	27.7817	25.4049	60.0530	21.1946	protein JINGUBANG-like
Cg8g005950	63.9457	78.5888	34.4133	53.0452	51.5030	34.6954	30.1321	72.2503	21.5335	uncharacterized protein LOC112498178
Cg8g005410	3.5920	2.8938	3.0999	2.3697	1.3224	3.5308	2.3999	0.9214	4.0789	uncharacterized protein LOC102614739 isoform X2
Cg8g005230	3.5216	2.2795	3.3062	3.1190	1.7733	3.4766	3.3956	1.6489	4.5998	peptidyl-prolyl cis-trans isomerase CYP40
Cg8g004610	18.7814	17.9403	11.5730	12.4581	13.4699	13.9073	10.8247	27.1354	11.2557	protein CYPRO4
Cg8g004600	120.3034	140.4271	5.3010	22.1857	41.8128	8.9016	1.9660	168.0981	4.9630	ethylene-responsive transcription factor ERF109-like
Cg8g004230	5.1706	6.0652	4.4326	3.8489	4.6480	3.7389	3.4778	7.5383	3.7945	CBS domain-containing protein CBSX1, chloroplastic-like
Cg8g004130	1.6209	1.2690	4.2225	3.5052	2.3783	5.7666	5.9745	2.5903	8.9046	LRR domain containing protein
Cg8g003880	10.0737	18.6682	3.3598	6.5775	12.2104	5.2924	2.5444	14.9113	3.7296	transcription factor MYB44-like
Cg8g003310	31.4326	30.7271	24.2665	36.9274	29.9117	22.4959	26.1897	51.1921	23.3313	leucine-rich repeat protein 2-like
Cg8g002750	0.7971	2.3000	0.3515	1.1696	0.6728	0.2489	0.3511	2.9655	0.6290	uncharacterized protein LOC102624813 isoform X1
Cg8g002730	3.4646	3.9071	2.2674	4.8971	2.1077	1.5397	2.9750	5.4290	2.6984	serine carboxypeptidase-like 18
Cg8g002460	14.7867	23.6625	7.1914	7.6558	13.7223	5.0972	5.2605	13.8214	5.5298	Formin-like protein 18
Cg8g001940	14.5740	9.5332	20.9599	24.6408	14.5091	26.8822	22.6943	9.9928	21.8776	PI-PLC X-box domain-containing protein DDB_G0293730
Cg8g000910	3.3167	3.9615	1.5375	0.5423	1.0812	0.4855	0.1657	1.4727	0.1482	L-ascorbate oxidase homolog
Cg8g000430	4.2482	4.3258	2.8595	3.5945	4.2660	2.7868	2.8215	7.7651	2.4541	probable serine/threonine-protein kinase PIX13
Cg8g000160	22.7851	32.9391	8.9545	11.7926	16.9631	8.9581	7.5345	37.3736	6.9353	3-hydroxy-3-methylglutaryl-coenzyme A reductase 1-like
Cg7g023910	2.2536	4.7870	0.3562	0.8547	2.1851	0.6810	0.3929	11.3905	0.9451	Transcription termination factor like
Cg7g023640	1.1092	1.1765	0.4464	1.2694	1.4334	0.5623	0.9822	3.3964	0.4168	hypothetical protein CUMW_026230
Cg7g023300	1.4073	0.4789	2.1213	1.7193	1.5084	3.4846	1.2276	0.8247	0.4247	hypothetical protein CISIN_1g022378mg
Cg7g022240	9.5079	12.2072	1.7251	2.0366	5.0491	1.1299	1.1326	18.4323	2.9350	protein TIFY 9
Cg7g022180	8.1934	11.6820	5.2362	7.6157	8.2965	5.5746	4.2211	9.9990	5.2126	Humj1 family protein
Cg7g021880	297.7502	122.4208	405.2311	293.8376	170.8223	400.8124	302.2416	172.5650	336.7568	nodulin-related protein 1-like
Cg7g020140	34.3226	53.9005	16.5706	12.0537	25.7343	10.9573	8.6305	39.2960	4.9945	probable galacturonosyltransferase-like 9
Cg7g020060	13.2941	20.4722	4.2850	3.9059	9.3339	4.1280	2.1754	21.2786	1.9943	metalloendoproteinase 2-MMP

Cg7g019990	17.5134	18.4249	11.1356	8.8760	13.7566	7.4323	7.3713	25.9345	13.0812	IAA-amino acid hydrolase ILR1-like 1
Cg7g019520	3.9847	2.4905	1.4760	1.6128	3.4653	2.1036	1.8319	5.8722	0.9612	U-box domain-containing protein 19-like
Cg7g019280	12.6265	20.5124	4.2662	11.5765	20.5065	8.0225	6.6688	52.2418	9.0007	death domain-associated protein 6-like
Cg7g018740	8.5461	8.6977	6.2152	5.7736	7.5878	7.4616	4.7036	14.0335	5.4071	ARM REPEAT PROTEIN INTERACTING WITH ABF2
Cg7g018530	37.3551	27.6501	21.4777	20.2127	18.8553	15.8948	12.7773	27.2061	9.6819	probable E3 ubiquitin-protein ligase XERICO
Cg7g017800	32.8624	23.7087	24.1224	31.0814	18.3187	22.1128	28.5184	12.5662	32.3006	small heat shock protein, chloroplastic-like
Cg7g015180	8.0643	9.1668	4.9314	6.1491	8.1732	4.2370	4.1781	10.3714	2.4848	probable calcium-binding protein CML16
Cg7g014840	105.6401	195.8911	57.6120	41.5527	79.7491	31.7230	21.4076	94.4100	18.9761	probable galacturonosyltransferase-like 3
Cg7g014820	70.0947	136.1342	40.8014	32.8356	94.8438	31.5316	23.6210	186.7477	54.2034	AP2/ERF and B3 domain-containing transcription factor RAV1
Cg7g014590	3.0162	4.2387	1.9799	3.1563	3.2417	1.7128	1.9513	5.7949	1.6909	Ist1 domain-containing protein
Cg7g014510	186.3601	61.3668	245.6500	216.8044	86.5611	220.5215	186.1514	69.0048	95.8884	protein NRT1/ PTR FAMILY 3.1
Cg7g014490	221.6031	353.9198	142.8263	226.7411	261.2570	139.2238	165.3510	398.5550	191.3996	uncharacterized protein LOC18044404
Cg7g012530	5.8929	4.8242	2.2846	4.6800	6.0863	3.4007	2.6770	12.7282	3.0457	aspartyl protease family protein 2-like
Cg7g012490	115.9828	52.5015	126.6740	136.3458	81.4381	144.0551	144.5210	90.5639	130.9278	GDSL esterase/lipase At2g42990-like
Cg7g006290	26.8807	35.5253	21.8184	28.3163	29.4697	21.4346	20.1906	40.2937	24.0834	Serine/threonine-protein kinase bur1
Cg7g004490	6.8734	9.3745	2.5386	2.5002	4.3113	2.3805	0.9512	8.1015	0.8189	heat shock 70 kDa protein 18
Cg7g004480	9.3997	9.4758	4.9105	8.6269	9.5346	5.2659	5.1166	16.9485	2.7591	heat shock 70 kDa protein 18
Cg7g004470	11.8886	14.3823	5.2223	1.8356	4.8903	1.8993	1.7504	6.5082	0.5918	heat shock 70 kDa protein 18
Cg7g004440	11.4917	11.0844	4.3925	4.1663	7.3248	3.1919	2.7683	15.0855	2.2015	heat shock 70 kDa protein 18
Cg7g004430	2.4042	3.9644	0.7189	1.9664	2.4929	0.3559	0.5048	2.6588	0.2748	heat shock 70 kDa protein 18
Cg7g003920	132.8352	144.6277	82.1204	122.7599	149.2493	62.9851	81.6888	176.6886	64.5860	Voltage-dependent T-type calcium channel subunit alpha-1I, putative
Cg7g003890	91.7366	89.4170	21.4317	36.6840	86.5088	34.8129	16.3742	155.4733	13.7334	probable WRKY transcription factor 70
Cg7g003730	1.5858	2.5676	0.2768	0.9959	1.9165	0.3238	0.4961	4.6870	0.2929	phenylalanine N-monooxygenase isoform X2
Cg7g003500	14.0247	15.3919	1.6641	6.6494	13.2265	3.1599	1.8782	37.3284	2.9308	hypothetical protein CISIN_1g033545mg
Cg7g003310	4.8118	10.9516	3.7195	8.4364	10.7215	5.1026	6.7296	10.2435	5.7827	peroxidase P7-like
Cg7g002780	59.0857	69.3091	37.3232	38.9275	51.9198	42.3161	34.7669	111.2289	28.5110	senescence associated gene 20
Cg7g002540	1.6825	2.5074	1.2839	1.2144	1.3034	0.8370	0.9740	2.3394	0.9257	dehydration-responsive element-binding protein 2A-like
Cg7g002150	33.9301	24.8777	35.7802	22.4873	22.6617	32.5425	21.5556	42.4675	18.9034	probable protein phosphatase 2C 25
Cg7g002050	216.1730	299.4032	41.0352	91.6758	166.4370	72.2775	36.0428	505.2239	57.4163	zinc finger CCCH domain-containing protein 29-like
Cg7g002040	9.0664	5.3185	0.5760	0.6944	4.7996	0.8729	0.2538	9.7966	3.1511	probable 2-oxoglutarate-dependent dioxygenase At5g05600
Cg7g001990	2.4424	3.1664	0.6290	0.4524	1.7179	0.4179	0.2505	5.4490	0.2290	putative serine/threonine-protein kinase-like protein CCR3
Cg7g001180	60.3171	86.8293	21.9766	19.6260	53.0669	23.1673	15.1330	197.7993	26.6555	nematode resistance protein-like HSPRO2
Cg7g000850	41.6902	56.8991	31.0549	33.9889	45.4677	32.3525	26.4589	79.6690	32.2464	probable E3 ubiquitin-protein ligase RHC2A
Cg7g000300	8.5317	1.7643	11.7859	14.9253	6.4008	10.4996	11.5695	6.1895	7.0167	18.1 kDa class I heat shock protein-like
Cg7g000070	10.0301	7.4428	9.1625	10.7053	3.4245	9.1358	8.0333	5.8996	15.0185	No match
Cg7g000020	16.2527	31.8112	11.7602	9.3133	23.4887	11.8085	7.7142	33.6866	14.6170	hypothetical protein CISIN_1g021888mg
Cg6g025030	308.4510	328.6351	60.9055	280.7090	377.7238	122.5613	97.4628	806.8344	127.9039	probable calcium-binding protein CML45
Cg6g024930	51.0875	75.6374	5.1571	10.2825	36.1442	8.8135	4.8791	161.6571	11.1193	amino acid transporter AVT1I-like
Cg6g024750	5.6688	7.9009	3.8579	3.3686	4.5021	2.4489	1.5993	4.6265	1.5454	Glutaredoxin domain-containing protein
Cg6g024360	78.7642	66.8846	39.7948	73.2677	84.3216	65.3995	45.6229	143.8982	43.1424	NAC domain-containing protein 71-like
Cg6g023620	4.6960	5.3883	3.2371	2.9999	2.8905	2.0907	1.8125	6.3148	1.9521	DUF1336 domain-containing protein
Cg6g020290	5.4538	8.1581	0.2819	1.5476	3.0714	1.2057	0.1397	11.0455	0.3808	abscisic acid 8'-hydroxylase 1
Cg6g020250	112.7945	123.8587	48.8551	70.6348	109.6013	41.9142	42.1573	234.1862	34.7367	No match
Cg6g020190	4.7101	1.9238	16.5231	27.7571	25.8573	29.1804	33.8216	13.0077	27.3341	protein GLUTAMINE DUMPER 6-like
Cg6g019330	2.8462	2.8947	1.4523	1.9477	1.6279	1.2043	1.4656	5.1367	1.9247	LEAF RUST 10 DISEASE-RESISTANCE LOCUS RECEPTOR-LIKE PROTEIN KINASE-like 2.1
Cg6g019200	5.3600	5.8691	5.2474	3.6653	4.2410	4.3564	3.5215	6.9599	3.1087	Octicosapeptide/Phox/Bem1p family protein, putative
Cg6g019030	14.1245	28.4980	2.2898	4.4837	12.7975	2.3931	1.9038	27.9740	4.2331	GDSL esterase/lipase 6
Cg6g018900	4.4456	7.2871	2.1994	3.9216	3.5578	2.5296	2.7238	6.2728	2.0510	uncharacterized protein LOC18040053
Cg6g018470	71.6130	83.7120	16.6503	25.4337	40.7124	17.1330	10.7480	78.5351	11.2890	probable calcium-binding protein CML23
Cg6g018280	110.3429	151.7811	57.3679	106.8676	112.2557	59.7766	59.4009	177.1810	63.8646	nudix hydrolase 18, mitochondrial-like
Cg6g017210	131.9941	217.1634	48.5816	96.3293	137.2880	56.3279	56.6411	353.3781	54.8797	calmodulin-binding protein 25-like
Cg6g017180	1.6722	1.5663	1.1575	1.2064	1.0030	0.3101	1.1955	1.4840	0.5708	vacuolar iron transporter homolog 2-like
Cg6g016810	30.7623	43.5048	20.7418	35.9592	55.6888	19.4488	34.6700	68.8061	22.1092	protein REVEILLE 1
Cg6g016530	8.6918	16.6402	2.1543	4.1964	7.2569	2.5184	1.9036	12.7266	2.5646	protein CHUP1, chloroplastic
Cg6g016150	26.3520	16.4291	0.3166	1.0434	11.3881	0.9735	0.0480	29.7367	0.5128	dehydration-responsive element-binding protein 1B-like
Cg6g015420	147.4229	222.6356	42.5657	101.5034	145.4363	52.7314	51.4491	308.8877	57.1720	uncharacterized protein LOC18038506
Cg6g014660	3.2905	5.0788	2.7877	4.5210	4.8823	2.6731	2.9780	7.1878	2.3355	NDR1/HIN1-like protein 26
Cg6g014500	17.2102	42.5585	1.3510	3.5759	9.8958	1.9197	0.5780	51.1884	1.2527	E3 ubiquitin-protein ligase PUB23
Cg6g014010	12.0874	17.5733	5.5168	9.5447	13.2702	6.2306	5.8363	35.5485	5.3361	syntaxin-121-like
Cg6g012530	7.7683	11.3735	6.0562	10.7641	13.0270	6.3207	6.6582	15.9940	5.7366	KS1 protein-like
Cg6g011970	24.1997	15.2137	6.8791	13.3435	17.2892	14.3934	5.1782	24.4342	2.2057	cysteine-rich/transmembrane domain A-like protein
Cg6g011490	2.8163	2.0910	3.6629	4.3823	2.2202	6.1329	4.2993	2.0353	7.9772	putative TTHA0068-like domain-containing protein
Cg6g011130	9.1443	12.9608	4.4861	10.4516	10.8013	7.7389	6.3051	21.7709	7.8482	Late embryogenesis abundant protein
Cg6g011110	11.2090	15.5898	7.7056	6.6460	8.6183	7.2675	6.5047	19.4020	5.6622	cyclin-dependent protein kinase inhibitor SMR11-like
Cg6g011100	28.1586	54.7806	9.3184	15.2266	30.8345	9.7731	7.8959	88.6204	10.7241	probable calcium-binding protein CML43
Cg6g010690	95.8879	79.2038	27.8325	31.9349	71.2343	29.1949	15.4303	94.3718	10.1700	probable WRKY transcription factor 70
Cg6g010380	3.2704	3.1661	5.1569	1.9733	1.9289	7.1013	2.1312	0.7219	4.6535	transcription repressor OFP1
Cg6g010360	9.2280	24.3117	3.3859	5.3630	11.0219	2.8447	2.1554	14.9509	2.3112	U-box domain-containing protein 16
Cg6g010160	2.3672	5.7005	1.1778	3.3221	5.9060	3.4162	2.2962	9.5882	3.6335	protein LURP-one-related 10-like
Cg6g010100	125.3898	156.4488	46.5607	58.9925	99.6439	45.3859	32.3564	245.1551	38.5202	protein LURP-one-related 10-like
Cg6g010050	3.2578	5.6677	2.1814	3.8795	5.2888	2.1305	2.9505	9.9134	2.4557	PLAC8 domain-containing protein/DUF2985 domain-containing protein
Cg6g009930	69.5317	142.4179	24.0601	55.7135	84.8329	31.5475	29.2511	305.4956	41.5353	Methyltransferase FkbM
Cg6g009750	35.3367	112.1939	35.3859	40.1314	62.1684	30.4317	46.1831	91.4248	99.1054	abscisic acid receptor PYL4
Cg6g009560	38.1295	50.1427	21.5834	30.0614	39.5784	28.5825	18.2349	72.6515	17.1825	lectin-domain containing receptor kinase VI.3-like
Cg6g008370	45.8932	62.7193	10.7014	33.8979	38.3628	12.8659	8.3880	88.7051	8.5260	protein PHLOEM PROTEIN 2-LIKE A1-like
Cg6g006680	2.9402	3.0341	1.5017	1.0352	1.4911	1.8276	0.6333	1.4512	1.0511	probable LRR receptor-like serine/threonine-protein kinase At3g47570 isoform X1
Cg6g005870	16.0563	14.4509	10.9085	12.3408	11.7877	10.3651	11.1691	22.2097	9.7946	phosphatidylinositol:ceramide inositolphosphotransferase 1
Cg6g005570	2.4369	2.9719	1.7981	2.1750	2.3647	1.7432	1.5089	4.9845	1.5453	F-box protein At5g49610-like
Cg6g005410	4.4823	4.7258	1.7363	3.0737	5.1504	1.4656	2.3420	8.2850	3.4561	cysteine proteinase inhibitor
Cg6g004920	23.8154	36.1047	12.3024	12.8832	19.7505	13.1281	14.0077	48.0437	14.5077	E3 ubiquitin-protein ligase RDUF2-like
Cg6g004830	3.4727	4.1878	1.5251	0.5708	1.6794	1.8133	0.1982	4.1759	0.2944	cyclin-dependent protein kinase inhibitor SMR4
Cg6g003560	7.4688	8.5260	2.4500	2.5448	4.7590	1.6278	1.9698	9.4649	3.2650	uncharacterized acetyltransferase At3g50280-like
Cg6g003540	5.6449	5.6524	1.3259	1.0749	2.7615	0.7259	0.6220	4.6648	1.5898	uncharacterized acetyltransferase At3g50280-like
Cg6g003530	5.9748	4.5767	0.8972	0.7029	2.9591	0.4209	0.7116	7.5703	1.3030	uncharacterized acetyltransferase At3g50280-like
Cg6g003110	332.6284	509.3895	190.3918	280.3749	414.5088	170.0352	211.6609	687.9184	189.3836	related to Polyubiquitin
Cg5g044340	1.6459	1.9249	0.7032	2.6683	2.6247	1.1384	0.7243	6.8515	1.1303	ethylene-responsive transcription factor 13
Cg5g043370	0.8034	0.8479	1.9809	2.8330	1.3520	1.6101	2.9655	0.5886	4.4351	S-adenosyl-L-methionine-dependent methyltransferase
Cg5g043350	4.9613	3.2178	4.2645	6.6790	3.9491	4.4509	8.5469	3.3239	4.9747	Flower, cultured cell, putative
Cg5g042620	4.3029	8.1636	0.7658	2.6322	6.4280	0.8280	0.7514	27.3717	0.5633	BON1-associated protein 2
Cg5g042230	56.5999	70.1318	15.3297	27.1428	44.0208	25.8099	18.4748	182.5342	20.3902	FT-interacting protein 1
Cg5g041630	19.0603	49.5084	18.5762	22.1340	37.5294	19.2149	21.5960	35.3535	19.3565	probable serine/threonine-protein kinase At1g01540
Cg5g041320	7.7957	14.7127	5.6494	8.1260	7.5380	6.7716	5.6832	14.1874	6.4234	delta(8)-fatty-acid desaturase 2
Cg5g040920	44.4058	39.4472	6.0968	18.1060	41.1219	15.5883	9.4611	161.8237	11.1259	probable WRKY transcription factor 46
Cg5g040870	6.2558	4.7658	6.5752	6.5574	3.4463	10.2775	8.9896	4.2955	13.2046	protein ROOT PRIMORDIUM DEFECTIVE 1
Cg5g040610	49.0114	48.6471	31.5771	27.3153	39.5624	32.3115	29.7028	79.1160	38.3145	transcription factor bHLH13
Cg5g040330	21.7196	21.6278	9.0055	17.6175	19.5381	17.0953	11.8008	40.7846	11.5681	AAA-ATPase At2g46620
Cg5g040130	32.9675	31.9842	16.1616	17.1646	11.2081	11.1378	19.4485	25.4651	16.4136	3-ketoacyl-CoA synthase 1
Cg5g039430	137.7634	130.9952	76.1156	116.5747	134.2379	57.8927	62.0431	159.4559	46.6641	uncharacterized protein LOC18047012
Cg5g039380	3.1869	2.6358	4.3921	2.1287	3.6225	5.1654	2.8771	2.1559	2.2249	meiosis-specific protein ASY3 isoform X1
Cg5g039260	27.9543	7.6342	28.8671	18.9494	15.5873	28.5114	32.8888	2.1060	22.5109	diacylglycerol O-acyltransferase 3, cytosolic
Cg5g039060	19.9435	18.2639	21.6503	16.9686	20.7973	15.7073	15.3294	30.7770	12.2874	TolB protein-related isoform 1
Cg5g038980	12.2023	13.8951	7.4323	5.6833	9.8526	7.0137	9.3397	26.4890	5.7209	probable protein phosphatase 2C 49
Cg5g038330	76.3575	131.9180	63.1863	65.9439	88.1483	50.3039	41.9454	119.5270	39.4792	bZIP transcription factor 53
Cg5g038230	35.1612	14.8381	44.1387	60.7586	32.7362	59.5828	39.7103	18.9328	51.4832	universal stress protein A-like protein
Cg5g038200	7.8416	8.6313	0.8771	3.8269	6.3103	1.8849	1.6096	32.2298	2.2869	DUF4228 domain-containing protein
Cg5g037860	15.2135	20.8967	4.5508	8.6792	12.6080	6.1178	5.1120	39.9253	6.0032	protein DEHYDRATION-INDUCED 19 homolog 4-like isoform X1
Cg5g037790	1.6695	2.7945	0.9621	2.6804	2.5826	1.2681	1.6744	3.6328	1.8977	ethylene-responsive transcription factor ERF071
Cg5g035890	19.8387	32.2819	0.9412	2.4586	10.9956	0.9111	0.4012	32.6611	0.5252	Tetrapyrrole biosynthesis
Cg5g034990	43.3832	45.2560	18.0881	22.9729	38.9374	16.1154	20.2471	88.1179	17.7428	BI1-like protein

Cg5g034980	75.6714	80.5336	54.4792	46.1601	87.2525	61.0090	45.9417	100.5161	62.6675	chlorophyllase-1, chloroplastic
Cg5g034360	0.8607	0.8362	0.2602	0.5384	1.0518	0.3167	0.1565	1.0974	0.3597	transcription factor bHLH3-like
Cg5g034050	17.1902	7.5481	1.7533	1.5017	7.3333	1.8014	0.8737	16.5753	3.9041	ethylene-responsive transcription factor 1B-like
Cg5g033290	108.6731	123.7900	39.2039	51.5733	89.3749	46.1751	50.6088	158.4915	63.0181	Tetratricopeptide repeat-like superfamily protein, putative isoform 2
Cg5g032570	127.1682	139.4213	55.0754	62.2774	84.4245	38.6114	34.6454	99.0607	35.7474	desumoylating isopeptidase 1
Cg5g032320	5.2848	3.7993	5.9626	8.1486	5.3260	5.8108	8.3882	3.6126	7.4469	uncharacterized protein LOC18049535 isoform X1
Cg5g031920	3.3597	6.7779	2.4393	3.0756	4.7488	1.6228	2.3612	7.9288	2.0284	cyclic dof factor 3-like
Cg5g031660	1.3513	2.7688	1.5040	1.6199	1.8216	1.0016	1.9650	2.6430	1.6861	plant cysteine oxidase 2-like
Cg5g031070	19.8450	25.7837	3.5752	22.7022	26.6381	5.4964	4.3435	36.9193	5.4337	probable calcium-binding protein CML45
Cg5g028120	8.2465	11.6034	6.1347	8.7245	8.8580	3.4390	4.9976	9.2948	4.1773	zinc finger BED domain-containing protein RICESLEEPER 2-like
Cg5g026660	2.7369	3.5666	1.4777	1.1207	1.4729	0.8042	0.7979	2.1733	0.9382	endochitinase A-like
Cg5g026630	5.2032	4.2043	1.9067	1.3246	2.2597	1.3458	1.3341	5.9445	0.6341	endochitinase EP3
Cg5g026620	1.8064	1.7532	0.2324	0.5927	2.0306	0.4561	0.2114	6.6994	0.2409	transmembrane protein
Cg5g026610	1.9132	1.9841	0.4757	1.8089	2.5321	0.8291	0.4010	8.8012	0.3520	transmembrane protein
Cg5g026350	10.3624	15.4837	1.9551	7.2419	9.7966	4.3544	2.1324	29.6596	3.0369	leucine-rich repeat receptor-like serine/threonine/tyrosine-protein kinase SOBIR1
Cg5g026310	4.4635	8.8522	1.2079	3.2927	4.4846	0.8664	1.5647	17.3409	1.4603	leucine-rich repeat receptor-like serine/threonine/tyrosine-protein kinase SOBIR1
Cg5g026270	1.2679	3.0662	0.7382	1.0708	1.8957	0.7405	0.6669	5.7263	1.0874	leucine-rich repeat receptor-like serine/threonine/tyrosine-protein kinase SOBIR1
Cg5g026260	7.0535	10.1689	5.2862	7.7760	7.8730	5.6944	6.1169	12.3785	6.3461	putative UPF0481 protein At3g02645
Cg5g025070	7.9969	14.5888	6.5528	4.6671	9.0341	4.3614	4.4064	12.6807	5.2040	putative 12-oxophytodienoate reductase 11
Cg5g023190	2.9983	4.0438	2.7784	4.0871	3.5374	2.0676	2.8500	5.9244	2.5015	F-box/kelch-repeat protein SKIP20
Cg5g022750	15.7121	31.2388	4.6176	4.7015	12.2346	3.4653	2.8357	34.7396	2.5653	LOB domain-containing protein 41-like
Cg5g021300	3.1927	4.9725	1.5885	4.3139	4.1697	1.8997	2.6154	6.2285	2.0287	disease resistance-like protein DSC1
Cg5g019820	49.4295	69.3969	18.9320	27.7074	35.8537	21.7526	18.7774	107.5268	19.0850	NAC domain-containing protein 62-like isoform X2
Cg5g019600	126.8620	216.4479	23.4262	91.1056	135.9177	40.6430	28.6654	375.5397	45.4939	Avr9/Cf-9 rapidly elicited protein 65
Cg5g019370	24.1491	29.9451	19.0820	40.7228	42.5708	19.2060	24.5802	47.9835	24.1230	auxin-responsive protein SAUR32
Cg5g019000	47.7708	41.5850	10.9722	8.3741	36.3325	10.4590	3.8679	70.9797	15.7606	mitogen-activated protein kinase kinase 9-like
Cg5g018690	1.6390	1.5947	0.4340	1.3462	1.6449	0.5459	0.7588	5.5968	0.8315	AAA-ATPase At2g46620-like
Cg5g017520	7.0190	6.3801	4.4662	4.3042	5.8939	3.9675	3.9776	13.2878	3.8442	cold-responsive protein kinase 1
Cg5g016820	11.7188	10.5383	7.0429	10.1634	16.8578	8.3047	9.2178	24.4648	5.7701	Oxidative stress 3
Cg5g016800	165.6780	221.7609	155.5298	292.5661	268.2355	128.9255	212.6251	306.6814	182.5107	programmed cell death protein 4-like
Cg5g016320	73.0760	32.4938	23.1011	16.0330	29.3302	27.6383	6.8621	48.0417	4.9647	9-cis-epoxycarotenoid dioxygenase NCED3, chloroplastic
Cg5g016280	3.0582	5.0154	0.9398	1.2827	1.6223	1.1674	0.7968	3.2377	0.3870	VQ motif-containing protein 1-like
Cg5g015780	1.7161	2.6265	1.1271	1.4532	2.4636	0.3834	1.1653	2.7211	0.4057	agamous-like MADS-box protein AGL62
Cg5g013920	142.9545	185.9098	109.6188	88.1102	114.2802	87.5893	69.9013	140.4391	75.2884	S-adenosylmethionine synthase 1
Cg5g012080	15.9011	22.9781	13.2409	12.6216	16.4166	13.8805	11.7278	26.0578	12.8736	protein PFC0760c-like
Cg5g012040	18.7496	19.2880	17.6801	11.4211	12.4943	12.6845	7.6850	33.8448	5.6383	UDP-glycosyltransferase 89B2-like
Cg5g012010	7.2403	8.1940	3.9362	5.1351	5.8132	3.7994	4.8965	10.5634	4.7061	phosphatidylinositol 4-kinase gamma 7-like
Cg5g011790	15.7865	19.4982	1.0423	1.8413	3.9137	1.1019	0.0857	5.3668	0.2361	dehydration-responsive element-binding protein 1B-like
Cg5g011060	1.4001	3.0383	1.2020	1.2506	1.9666	0.6398	0.9980	2.6511	0.9282	UDP-glycosyltransferase 83A1-like
Cg5g010520	1.9381	1.6982	2.7554	2.5949	1.9387	4.7013	6.4220	1.7439	4.2466	pentatricopeptide repeat-containing protein At5g66520-like
Cg5g010410	3.1485	5.2261	1.2890	2.2603	2.9552	1.6468	2.4622	7.5044	2.7073	NAC domain-containing protein 100-like
Cg5g010280	12.6534	6.4752	19.2916	22.3724	12.3296	22.2679	29.9970	9.1807	35.2965	Disulfide bond formation protein B 2, putative
Cg5g010020	4.1179	5.8282	1.3287	1.3031	2.0738	0.9516	0.7877	5.0521	0.6129	thaumatin-like protein
Cg5g009930	7.1169	14.8989	1.9260	6.5315	16.1048	3.0225	1.5377	25.9077	2.6259	BON1-associated protein 2-like
Cg5g009710	6.9953	6.9872	0.0979	1.3635	2.0213	0.3875	0.0450	7.7056	0.1154	dehydration-responsive element-binding protein 1A-like
Cg5g007900	9.4740	10.7213	8.8543	7.6659	7.7937	7.8877	6.2061	12.2449	6.4227	O-fucosyltransferase 30
Cg5g007890	27.6033	36.4621	17.4225	17.1257	26.2343	16.7672	16.4201	55.9374	18.3307	transcription factor KUA1
Cg5g007760	207.8571	343.0231	45.8214	109.0046	212.4256	57.3826	33.9314	359.7491	40.6802	ethylene-responsive transcription factor 5-like
Cg5g007750	72.5716	143.5010	8.4518	15.2640	79.8488	9.3530	4.5930	168.0335	18.8314	ethylene-responsive transcription factor 2-like
Cg5g007670	4.4488	3.5754	2.0634	1.8033	2.7030	1.8383	2.5790	4.6327	2.8376	G-type lectin S-receptor-like serine/threonine-protein kinase LECRK3
Cg5g006750	8.9168	11.7590	1.9509	3.5937	7.1763	1.9180	0.8693	29.3474	0.9140	putative calcium-binding protein CML19
Cg5g005590	100.0341	108.9685	53.8052	69.5519	102.2611	46.1405	83.3654	215.9216	38.2489	Octicosapeptide/Phox/Bem1p family protein
Cg5g005560	4.7874	3.4115	4.7846	10.3541	5.1833	5.4138	7.2620	3.1723	3.5058	U-box domain-containing protein 33-like
Cg5g004810	2.2969	2.2985	0.6357	1.5071	3.0157	1.1967	0.6285	5.3467	0.6281	transcription factor bHLH162-like
Cg5g004720	20.8187	27.3422	11.6484	19.6888	23.4681	13.2138	13.2114	38.6342	14.1141	hypothetical protein CUMW_282940
Cg5g004460	64.4037	65.3784	16.0971	55.1736	70.5906	19.8624	25.8547	140.8563	23.7552	CDP-diacylglycerol-glycerol-3-phosphate 3-phosphatidyltransferase
Cg5g004120	7.3349	5.9536	3.7440	2.7164	2.5424	2.7406	2.6423	6.4652	3.7556	glucan endo-1,3-beta-glucosidase
Cg5g003460	2.2870	1.0900	1.7173	3.1473	1.8100	2.9248	1.0162	1.1923	2.2653	mitochondrial outer membrane protein porin of 36 kDa-like
Cg5g002540	40.0192	68.6298	7.1409	12.7822	35.9395	9.2297	5.9440	116.8254	7.9149	BTB/POZ domain-containing protein At5g41330
Cg5g002190	10.5140	9.5249	3.4149	3.0557	5.4365	3.0842	2.4731	24.5748	2.9682	1-aminocyclopropane-1-carboxylate synthase
Cg5g001320	8.5392	6.3945	5.7101	7.9937	7.1639	5.3306	4.2643	9.4392	3.2049	RING-H2 finger protein ATL20-like
Cg5g001080	185.5996	214.3655	98.4141	194.4218	213.6696	124.3332	119.0779	408.0068	119.0818	B2 protein-like
Cg5g000800	16.9892	19.2268	13.2490	14.8816	17.3864	15.6074	13.9516	31.4802	14.5622	WD repeat-containing protein YMR102C-like
Cg5g000600	1.2235	2.1849	1.0716	1.8060	3.1271	1.2149	1.2747	4.5449	0.6447	uncharacterized protein LOC102611678
Cg4g024380	3.1568	4.5404	1.3908	1.4132	2.4084	1.0687	1.5973	8.0260	1.7925	calmodulin-binding protein 60 E
Cg4g023490	131.1777	218.2038	69.2224	75.4399	120.0675	59.3882	33.4783	184.5430	39.1209	UDP-glucuronate 4-epimerase 1
Cg4g023410	94.6834	106.5522	37.1756	54.1297	86.0770	36.4763	28.6045	175.2428	32.2234	tetraspanin-8-like
Cg4g022580	2.4444	2.6755	1.0394	1.0016	1.5849	0.5608	0.3965	3.2559	0.2286	hypothetical protein CUMW_111780
Cg4g022420	5.6485	9.3293	9.9962	24.1010	16.1548	10.6088	19.0091	9.1067	16.7221	protein GLUTAMINE DUMPER 3
Cg4g022210	5.3789	13.2481	1.2265	1.6512	5.7036	1.3126	0.8357	14.3172	0.7685	probable xyloglucan endotransglucosylase/hydrolase protein 23
Cg4g022200	1.4321	3.8734	0.5358	0.9033	1.0790	0.5213	0.0806	3.1123	0.2999	probable xyloglucan endotransglucosylase/hydrolase protein 23
Cg4g022140	29.8522	61.3293	16.4954	26.9386	34.0102	40.5061	37.5297	76.2832	32.1533	xyloglucan endotransglucosylase/hydrolase protein 22-like
Cg4g021000	8.9022	7.2009	2.8357	1.9022	5.4124	1.6291	1.5062	4.8959	0.8583	transcription factor bHLH35
Cg4g019710	87.7863	142.6958	89.2537	91.3548	133.5832	87.5244	144.9209	161.9512	55.9055	monogalactosyldiacylglycerol synthase 2, chloroplastic
Cg4g019450	77.3755	112.1643	47.4376	88.7338	99.4367	57.2465	50.1198	108.9292	50.8804	calmodulin-like protein 3
Cg4g019190	9.6228	9.9590	6.1297	8.0683	10.5242	4.9055	5.6052	25.1219	5.3588	probable WRKY transcription factor 50 isoform X1
Cg4g018880	10.9306	11.7968	17.4824	11.5896	5.4960	11.8968	14.3686	3.6276	28.2955	thermospermine synthase ACAULIS5
Cg4g018670	16.5335	13.5767	9.1847	10.5103	18.8021	9.1233	10.8151	25.3516	12.1379	serine/arginine repetitive matrix protein 2
Cg4g018600	2.2608	2.3210	0.3653	0.6538	1.1413	0.5301	0.2044	2.7868	0.5421	Arginine--tRNA ligase
Cg4g018510	7.5993	10.4766	1.2444	1.3369	5.6546	0.9911	0.2798	20.9223	2.0095	ethylene-responsive transcription factor ERF017-like
Cg4g018300	28.5724	20.1517	41.3131	28.7605	26.3447	67.2145	19.3885	14.7754	16.4068	pectinesterase/pectinesterase inhibitor PPE8B-like
Cg4g018280	47.6000	31.8640	59.2838	43.9836	64.0394	107.0228	26.9231	16.8137	34.2952	probable pectinesterase/pectinesterase inhibitor 12
Cg4g017780	284.0103	274.9813	91.8158	62.1159	151.3058	56.7639	41.6621	356.2121	157.4081	protein TIFY 10A
Cg4g017520	5.6540	6.0200	1.8218	2.1198	3.0450	2.3033	1.5833	11.7173	2.1110	serine/threonine-protein kinase RIPK
Cg4g016820	2.5297	4.3259	1.0669	1.4124	1.8671	0.9456	0.4230	2.2553	0.1868	disease resistance protein RPM1
Cg4g016810	16.3549	18.6217	6.2096	10.3096	12.2539	6.8574	4.2850	19.3722	3.0857	disease resistance protein RPM1
Cg4g016210	3.7117	4.1358	2.7064	3.6877	3.7037	3.0600	2.6820	5.5882	3.1557	calmodulin-binding transcription activator 4-like
Cg4g015900	1.5071	1.9086	0.2894	1.0307	1.2225	0.8811	0.4908	3.0505	0.5173	disease resistance-like protein DSC1
Cg4g015400	0.9563	2.5346	0.0968	0.4921	1.5436	0.1884	0.6579	4.3781	0.8335	ethylene-responsive transcription factor TINY-like
Cg4g014430	3.9966	1.8218	5.4264	3.8044	3.3101	5.2761	5.8720	3.4940	3.9092	transcription repressor OFP13
Cg4g013040	5.2787	11.1930	3.9793	3.3654	5.5345	2.2888	1.4195	6.8180	1.9669	pirin-like protein
Cg4g012880	13.0724	6.4902	17.8481	17.7557	10.3319	18.4953	20.9117	13.1474	12.5759	putative BPI/LBP family protein At1g04970 isoform X1
Cg4g011600	578.4302	827.3455	263.3227	303.5904	365.0443	148.4901	161.9285	614.2491	151.0643	probable calcium-binding protein CML27
Cg4g010680	1.9747	1.4530	4.2631	8.7868	5.1671	4.0019	4.8225	2.1122	4.8168	hypothetical protein CUMW_120960
Cg4g010160	472.2446	642.0990	168.3120	252.6945	489.8705	153.2595	170.7214	842.9983	130.6570	ethylene-responsive transcription factor 4
Cg4g008450	21.7160	27.9988	3.1232	6.9466	17.0451	7.3274	4.9782	96.9277	5.9662	probable pre-mRNA-splicing factor ATP-dependent RNA helicase DEAH5
Cg4g008310	30.3342	8.8171	49.6547	61.1388	32.6005	83.9571	66.6449	37.8925	75.7073	strigolactone esterase D14
Cg4g007900	41.5263	12.3444	49.0608	40.0711	22.5200	40.8787	42.4100	24.7230	36.1405	SOUL heme-binding family protein isoform 1
Cg4g007890	33.9469	42.6022	17.0271	16.2427	19.5560	11.1519	9.0293	36.9430	7.2154	3-ketoacyl-CoA synthase 11
Cg4g007050	17.8855	24.1478	7.2659	6.2080	10.9462	4.6526	4.5055	20.1523	3.0044	blue copper protein-like
Cg4g006940	4.2070	5.1884	2.9251	3.1110	4.1309	2.1664	1.8224	4.0039	1.6872	auxin-responsive protein IAA29-like
Cg4g006890	16.4417	17.6047	5.2542	7.7880	12.7125	7.5584	6.1879	35.8146	7.4061	IQ domain-containing protein IQM1
Cg4g006770	9.3336	19.5717	3.1836	6.0722	11.3646	3.9671	4.1398	36.1547	4.0439	probable leucine-rich repeat receptor-like protein kinase At1g68400
Cg4g006410	11.1116	9.0696	6.5767	6.3947	8.2426	8.1999	7.1880	17.7225	7.5257	ferrochelatase-2, chloroplastic
Cg4g006370	0.7442	1.3179	0.4057	0.7071	0.6455	0.1723	0.2314	1.2366	0.5648	serine hydroxymethyltransferase 3, chloroplastic-like
Cg4g006150	2.1769	2.6487	1.6502	0.7671	1.1088	0.6316	0.8038	2.4632	0.5403	ectonucleotide pyrophosphatase/phosphodiesterase family member 3-like
Cg4g004020	4.5576	7.0672	3.4317	2.8217	4.3133	2.9807	2.1001	10.0547	2.2025	ankyrin repeat-containing protein At5g02620
Cg4g001810	35.2068	50.0358	10.6994	9.8645	13.2047	7.0034	2.3420	19.4684	3.3731	uncharacterized protein LOC18053981
Cg4g001480	34.2452	13.4617	45.0897	41.1297	36.0312	58.5505	39.6595	17.0669	18.6178	CBL-interacting serine/threonine-protein kinase 25-like

Cg4g001420	7.9794	10.9558	3.6749	5.5640	5.9588	3.2550	2.8451	9.8048	2.5511	chorismate mutase 2 isoform X2
Cg4g001000	57.8695	33.6842	62.7425	91.8406	74.5795	68.6126	85.3689	38.8656	56.6749	aspartyl protease family protein At5g10770-like
Cg4g000690	62.4717	76.4327	34.2609	62.5533	63.1465	37.4980	53.8024	116.1343	56.7585	probable WRKY transcription factor 17
Cg4g000190	88.0031	317.0126	49.1170	41.5027	127.3877	33.5500	20.9959	137.3016	47.0795	gamma-glutamylcyclotransferase 2-1
Cg3g026040	7.2978	7.7282	2.5362	3.5538	5.0546	3.6810	2.7217	15.0727	2.9732	protein LYK5
Cg3g024300	67.9679	22.2192	164.5847	88.7930	52.0172	138.6127	128.1565	52.8685	120.0691	splicing factor, proline- and glutamine-rich
Cg3g024160	53.9913	50.9690	31.3551	12.7508	30.8144	14.4195	4.5199	32.0490	9.5932	chaperone protein dnaJ 11, chloroplastic
Cg3g022060	56.6568	69.8690	39.9921	33.3611	38.2841	27.3654	25.4719	68.5035	38.1748	allene oxide synthase 1, chloroplastic
Cg3g021930	31.6707	32.6878	24.5296	20.4450	28.4221	22.0544	21.7154	63.3756	22.1110	Thylakoidal processing peptidase 1
Cg3g021780	34.7820	78.0564	14.9978	19.2822	50.3297	18.5593	17.9665	177.4471	24.4434	hepatoma-derived growth factor-related protein 2
Cg3g021630	3.5052	4.2499	2.9674	4.0762	4.2842	3.0216	2.9979	6.0787	3.6245	probable carboxylesterase 2
Cg3g021510	40.1337	24.4620	65.7942	50.5955	42.9187	78.4823	51.1601	22.2010	54.6609	BRI1 kinase inhibitor 1-like
Cg3g020860	106.3063	236.0470	20.2717	53.6401	102.5473	28.7465	22.8762	256.7827	35.7830	transcription factor MYB44-like
Cg3g020670	1.8492	2.6760	0.8968	1.2194	1.9687	1.1867	1.1061	1.6968	1.1763	tyrosine decarboxylase
Cg3g020630	14.0791	34.1539	15.2805	13.1393	20.3061	15.6323	14.0590	25.3024	15.2468	patellin-3-like isoform X1
Cg3g020260	14.0522	15.5538	5.2601	9.8536	12.2546	6.1784	6.6231	35.8882	6.2173	leucine-rich repeat receptor-like tyrosine-protein kinase PXC3
Cg3g018400	109.9544	161.1765	41.5468	63.7782	88.4626	43.1917	31.7870	226.0913	40.3557	UPF0496 protein 4-like
Cg3g018200	8.0037	17.2392	1.7394	5.9623	10.8860	2.7734	2.1179	37.7398	3.2470	U-box domain-containing protein 28-like
Cg3g017190	41.2404	40.1745	21.7311	44.7611	50.6818	25.4526	27.8075	70.0620	28.2592	probable calcium-binding protein CML44
Cg3g016920	4.8583	9.6171	4.7735	2.9070	1.8552	1.7303	3.1214	15.8560	6.2622	amino acid permease 3-like
Cg3g016770	131.6980	93.6382	91.8611	72.1974	90.1739	68.8501	64.1894	199.0405	70.2575	NAC domain-containing protein 2
Cg3g015210	264.5447	310.2368	177.0958	214.7431	240.9821	170.1626	161.6953	405.7760	150.4144	ethylene-responsive transcription factor RAP2-4
Cg3g014770	26.4371	60.3550	4.6724	4.4445	11.2625	3.3212	1.7735	47.7912	1.8482	protein EXORDIUM-like 2
Cg3g014760	18.6572	43.7288	11.0924	9.7609	14.1449	7.0576	8.9537	49.1491	11.9033	protein EXORDIUM-like 2
Cg3g014500	4.7683	6.1379	2.6985	7.3679	7.8117	3.6669	4.9889	11.4216	4.2534	RING-H2 finger protein ATL11-like
Cg3g014360	22.9802	17.8579	17.7748	18.3701	18.5362	13.8278	21.1246	42.3898	11.4790	dihydrofolate reductase
Cg3g014300	19.5135	23.4490	10.2548	13.1198	13.2174	9.0054	5.5953	17.7054	6.2144	clustered-asparagine-rich protein-like
Cg3g014180	225.4381	319.7933	40.1344	95.4167	177.7167	58.6968	33.2200	535.2568	39.1656	mitochondrial uncoupling protein 5
Cg3g013180	22.4212	29.5201	11.6984	4.9609	11.3836	7.4168	3.4309	29.9991	3.0454	probable xyloglucan glycosyltransferase 12
Cg3g013160	1.8409	2.4724	1.1316	0.8040	1.3601	0.5887	0.4336	1.4994	0.2586	amino acid transporter AVT6A-like
Cg3g012860	1.8394	2.5409	0.5172	1.6107	2.5408	0.9504	0.7312	8.0491	0.8742	uncharacterized protein LOC102626963
Cg3g012790	56.8538	69.9645	25.7696	38.3282	54.6140	21.9823	27.7956	92.6519	23.9020	U-box domain-containing protein 4-like
Cg3g012710	789.7725	1499.5032	191.8424	425.6033	816.6111	195.0996	166.7162	1492.3397	154.0921	zinc finger protein ZAT10-like
Cg3g011940	1.5786	2.4830	1.0632	1.4899	1.3904	0.8125	1.1383	2.4210	0.9528	probable RNA helicase SDE3
Cg3g004030	5.8518	4.6140	0.8272	0.7810	3.6064	0.5806	0.5944	13.6225	1.6053	RNA demethylase ALKBH5-like
Cg3g002210	2.6098	1.2105	0.4700	0.3463	2.3453	0.5322	0.6911	2.5209	3.0806	cysteine proteinase inhibitor 6-like
Cg3g002080	1.6381	1.9046	0.8642	1.2364	1.1797	0.2517	0.6018	2.1598	0.7631	No match
Cg2g047350	5.8187	2.7305	12.0427	7.9827	3.9032	15.9890	12.0327	4.9978	16.2529	NAC domain-containing protein 35
Cg2g046910	1.2398	1.3885	3.0142	1.6077	1.6558	2.0378	1.9889	1.4102	2.6674	BTB/POZ domain-containing protein At5g47800 isoform X1
Cg2g046370	5.2882	2.6802	7.5559	9.8172	6.2930	10.4293	7.9853	5.3629	8.0194	microtubule-associated protein 70-5 isoform X1
Cg2g046190	51.0323	67.5609	16.6648	42.4081	53.1087	26.9316	25.1219	172.0376	28.4119	scarecrow-like protein 13
Cg2g045420	8.5931	9.0055	3.9028	4.6596	5.8552	4.2141	4.0779	20.3006	4.1527	probable WRKY transcription factor 31
Cg2g044980	41.1675	38.4702	5.0138	5.8055	19.0048	5.1788	2.2430	43.3441	6.4229	protein TIFY 5A-like
Cg2g044950	9.5917	16.2093	4.0559	1.4294	6.8007	3.9827	0.5861	6.2712	1.4079	probable 9-cis-epoxycarotenoid dioxygenase NCED5, chloroplastic
Cg2g044910	12.5007	18.3056	10.4304	11.1649	17.6750	5.6716	6.5882	14.7443	7.6496	VQ motif-containing protein 1-like
Cg2g043750	39.3590	49.2411	17.2267	34.0993	41.9612	22.5930	20.4859	77.9799	18.8915	U-box domain-containing protein 17
Cg2g043630	13.2626	24.4982	7.2739	7.4788	10.5493	4.3036	3.3523	16.7227	3.1871	uncharacterized protein LOC102628675
Cg2g042870	21.8335	18.7440	11.2837	11.6944	13.9557	6.8568	8.3063	31.5132	8.3862	pathogenesis-related genes transcriptional activator PTI5-like
Cg2g042710	43.0511	46.4230	27.1678	21.3281	33.4903	19.0709	13.2950	39.9357	10.2519	glutaredoxin-C9-like
Cg2g042530	86.3065	118.8251	32.3934	72.5400	104.7156	60.1267	56.6422	255.7163	53.1565	ethylene-responsive transcription factor 4
Cg2g042510	10.0740	11.8423	4.2135	3.4774	5.7361	3.4230	2.7178	15.3445	4.4825	ethylene-responsive transcription factor 12
Cg2g041300	17.6437	26.0726	4.0593	5.2931	11.6850	3.0117	2.2978	50.9683	3.4355	F-box protein At1g61340-like
Cg2g040680	1.4423	3.2030	0.9865	1.6179	2.3981	1.1501	0.5258	2.9083	0.6685	cysteine-rich repeat secretory protein 38-like
Cg2g040420	1.5058	0.8141	1.9401	2.1103	0.9921	3.0988	2.3161	1.2025	2.9172	thiamine thiazole synthase 2, chloroplastic
Cg2g040090	14.9061	22.2081	12.8683	13.0378	17.4077	11.0218	11.4883	28.3666	22.9364	15.4 kDa class V heat shock protein
Cg2g039720	18.9590	26.4985	3.9880	3.8207	10.9815	3.7165	2.7536	22.7091	2.5350	phospholipase A1-Igamma1, chloroplastic-like
Cg2g039080	43.7004	69.9900	13.4850	13.5889	38.1778	15.1553	5.9740	112.3661	13.6705	ethylene-responsive nuclear family protein
Cg2g038950	24.9853	21.5362	17.4955	9.0092	12.0808	13.8850	8.4638	30.0904	11.4324	berberine bridge enzyme-like 21
Cg2g038880	9.2985	10.5398	2.2481	5.3918	9.1548	3.5362	3.1019	23.5549	2.2011	berberine bridge enzyme-like 18
Cg2g038100	4.2480	4.9341	1.5722	1.4932	2.2088	1.5556	1.3890	5.5740	1.0947	protein argonaute 2-like
Cg2g037730	0.5994	0.1751	1.0653	0.6060	0.1376	1.5651	0.5851	0.0940	1.0793	pentatricopeptide repeat-containing protein At2g35130
Cg2g035990	1.6862	2.5518	1.0239	1.9468	1.5898	1.2393	1.2368	5.2323	1.0817	glycosyltransferase BC10
Cg2g034540	4.6695	6.6597	3.1296	6.0542	4.9584	3.6303	3.4735	11.5255	3.5067	hypothetical protein CISIN_1g028323mg
Cg2g034130	98.1676	169.2972	32.8634	26.9986	70.0519	24.7790	17.8943	142.9350	22.5151	hypothetical protein CISIN_1g029256mg
Cg2g033540	15.4158	7.0671	18.6145	20.2605	14.6256	28.8912	19.4624	14.6503	25.9915	phosphoglucomutase, putative
Cg2g033320	83.8664	47.2327	90.0234	112.5220	52.2952	81.3891	67.4766	45.5647	61.7453	protein PLANT CADMIUM RESISTANCE 2-like
Cg2g033160	2.3054	3.5697	0.8043	1.4339	1.7854	0.8441	0.3965	2.1427	0.3549	cytochrome B5-like
Cg2g032040	68.1419	98.4591	48.0062	68.7615	80.8576	43.9870	47.0977	96.5809	44.4703	uncharacterized protein LOC102608697
Cg2g031640	22.2835	29.3894	9.6906	8.7371	16.5674	6.2466	5.5692	34.6917	5.8913	hypoxia-responsive family protein
Cg2g030750	121.9735	137.8434	52.3431	85.3062	122.0263	53.9686	56.4086	276.6371	44.2618	E3 ubiquitin-protein ligase ATL6-like
Cg2g029780	1.9367	5.1562	0.8106	2.2852	3.1485	0.9255	1.0338	7.6786	1.5477	protein PLANT CADMIUM RESISTANCE 2-like
Cg2g029070	10.7229	18.6771	0.4101	1.7419	8.1958	1.6454	0.3670	35.3360	0.9155	U-box domain-containing protein 21
Cg2g028980	2.1951	1.0816	2.8885	1.9741	1.0928	5.5848	2.8210	1.4397	3.9740	histidine kinase 4
Cg2g028250	7.3933	10.7946	2.4498	3.6566	3.2076	1.9795	1.2090	5.2175	1.3446	putative CCR4-associated factor 1 homolog 8
Cg2g026830	1.7174	1.0758	1.8248	0.9988	1.5335	2.7902	1.8247	0.5385	0.9937	subtilisin-like protease SBT5.3
Cg2g026810	43.3453	56.0897	16.2699	48.9360	74.2912	27.0961	19.7553	137.6512	17.4367	heavy metal-associated isoprenylated plant protein 16-like
Cg2g024840	636.8084	881.7868	170.4265	356.9494	361.3692	139.1874	117.9607	747.8067	115.1181	late embryogenesis abundant protein Lea14-A
Cg2g023860	1.4083	0.3723	3.1003	6.2783	3.2882	7.6583	7.3797	2.0181	6.1274	receptor-like protein EIX1
Cg2g023550	14.3682	17.2236	7.8326	11.0353	11.9658	5.6245	5.2821	13.3567	4.1899	protein NDR1-like
Cg2g022960	42.2957	66.2491	6.3994	12.4450	36.6541	10.4185	6.8407	183.5955	10.0471	scarecrow-like protein 21
Cg2g022720	3.6963	1.4878	10.6297	6.0237	1.7792	13.0882	10.6437	2.4398	23.7501	transcription factor bHLH123 isoform X1
Cg2g022550	349.5078	575.2794	137.1413	225.4158	290.1349	128.1736	87.4880	567.0552	116.4336	calcium-binding protein PBP1
Cg2g022360	14.7241	15.5157	10.6987	14.0168	14.3593	12.0762	11.8910	25.2167	10.4899	receptor-like serine/threonine-protein kinase SD1-8
Cg2g022110	20.7689	25.5858	18.8349	18.4155	26.7237	18.0087	18.2652	37.7448	23.1599	zinc finger protein CONSTANS-LIKE 1-like
Cg2g021360	10.8011	4.4943	6.1264	5.6293	7.4481	4.6198	3.8528	16.6123	4.7715	NAC domain-containing protein 72-like
Cg2g020060	15.7876	26.7240	4.9052	5.4863	11.8568	6.0309	4.5211	41.3334	3.7204	calcium-transporting ATPase 12, plasma membrane-type-like
Cg2g019790	4.9203	4.8727	3.1476	2.9559	3.4173	2.6250	2.5996	9.9994	2.3474	cytochrome b561 and DOMON domain-containing protein At5g35735-like
Cg2g019210	332.9344	474.7348	213.7828	116.0488	180.2009	86.2507	69.6852	281.1606	64.8277	1-aminocyclopropane-1-carboxylate oxidase
Cg2g018660	1.8690	3.0394	0.9464	0.8240	1.8359	0.7136	0.6748	3.3701	1.1135	probable WRKY transcription factor 31
Cg2g018630	17.6091	12.7192	3.9572	3.1689	8.7408	2.9081	1.1600	7.1324	1.5665	mitogen-activated protein kinase kinase kinase 18-like
Cg2g018600	46.1684	63.5828	24.6009	32.6542	32.5856	18.3462	16.8473	41.4257	14.0493	B-cell receptor-associated protein 31-like
Cg2g017550	13.2155	21.9309	7.5478	7.5150	13.3800	3.4611	2.9986	13.0919	3.5328	putative FASCICLIN-like arabinogalactan protein 21 precursor
Cg2g015910	4.8580	3.1972	5.1242	4.8749	2.0853	6.6954	6.0335	2.3358	11.9683	thioredoxin-like fold domain-containing protein MRL7L, chloroplastic
Cg2g013640	53.5273	39.5090	25.3009	33.0045	62.5137	33.1044	23.8762	68.5727	18.2098	transcription factor bHLH162-like
Cg2g013490	1.3634	1.7210	0.6536	2.6011	2.4453	0.4965	1.1225	4.3429	0.7118	rust resistance kinase Lr10-like
Cg2g013290	268.2700	355.1709	96.5614	177.1214	282.3825	114.7030	102.8669	593.8358	85.5999	ethylene-responsive transcription factor 9
Cg2g012670	3.9965	1.6212	2.1028	2.5987	1.0352	2.1923	3.4736	1.1610	3.0559	small heat shock protein, chloroplastic-like
Cg2g012180	11.2958	13.4976	5.1403	8.8983	7.8180	6.2011	5.6620	14.5477	5.2661	thaumatin-like protein 1
Cg2g010320	516.0830	847.1796	451.9372	543.1572	774.4413	367.4702	538.3936	1019.7401	249.0634	glycerophosphodiester phosphodiesterase GDPD1, chloroplastic-like
Cg2g010310	52.6728	86.9145	14.8334	31.7471	41.5853	13.9063	17.9984	94.4469	15.4689	NAC domain-containing protein 83-like
Cg2g009980	5.8570	9.2860	4.4629	3.7568	5.7049	3.1641	4.0832	11.0950	4.9321	GEM-like protein 5
Cg2g008970	5.3291	6.5131	4.8497	4.5413	5.8096	5.2722	3.1601	7.3248	5.8892	uncharacterized protein LOC107175629
Cg2g008190	27.3128	37.5833	15.2639	5.3533	9.7692	8.7775	1.5791	14.6252	2.3614	glutaredoxin-C9-like
Cg2g007450	1.3889	0.6664	1.7195	2.6794	1.3359	2.5982	2.0464	1.1881	1.5745	SUPPRESSOR OF GAMMA RESPONSE 1-like isoform X1
Cg2g007230	1.7013	1.1855	0.1086	0.1820	5.6171	0.3145	0.3253	3.6871	1.6583	transcription factor MYB15-like
Cg2g006200	18.0655	22.7593	13.4011	11.3676	17.8070	11.0406	7.6668	18.2930	6.5727	NHL domain protein
Cg2g006000	43.1645	68.5724	23.1584	22.2345	30.8926	24.0703	23.6291	46.6246	32.3066	seed maturation-like protein
Cg2g005600	116.3949	222.2182	41.6510	42.6316	76.2656	24.8178	19.1264	168.4099	21.5518	NDR1/HIN1-like protein 13
Cg2g004360	11.7054	17.3121	2.5186	4.5123	11.7931	3.4127	3.3334	47.2261	4.2371	MDIS1-interacting receptor like kinase 2-like

Cg2g004340	10.8396	15.8442	2.1074	4.0774	10.1002	2.3779	2.4918	34.7085	3.1327	MDIS1-interacting receptor like kinase 2-like
Cg2g003790	1.7663	2.9689	1.2483	1.5215	1.8932	1.1716	1.2325	3.0814	1.0815	disease resistance protein RPP13-like
Cg2g002910	1.9578	3.3306	0.8336	1.2013	2.2534	0.8455	1.1127	3.1863	1.3255	putative UPF0481 protein At3g02645
Cg2g002900	4.0225	5.4357	1.8093	1.7012	2.6223	1.7812	0.9029	3.4504	0.7847	UPF0481 protein At3g47200
Cg2g002880	4.8759	7.7959	3.4049	3.2902	4.5263	1.5881	1.2803	5.1127	0.9692	putative UPF0481 protein At3g02645
Cg2g002860	17.4025	34.9136	4.1390	4.8561	11.3470	3.0202	1.0699	12.8815	1.1915	UPF0481 protein At3g47200
Cg2g002300	94.2977	139.2882	17.7599	67.1611	99.1615	20.7978	19.0525	154.8869	24.4030	hypothetical protein D5086_0000323660
Cg2g001440	4.6129	9.1916	3.1772	16.0967	14.6966	7.3111	4.2304	16.4850	4.3768	peroxidase 15-like
Cg1g024870	9.2938	10.1736	6.5126	6.5545	6.5767	5.8718	5.4802	17.3995	6.2871	probable LRR receptor-like serine/threonine-protein kinase At3g47570 isoform X1
Cg1g024280	276.3456	346.2067	57.2815	40.3932	139.7261	32.3252	13.8749	442.6455	17.2047	NAC domain-containing protein 2-like
Cg1g023710	30.2210	35.1111	24.6714	18.0785	22.2422	15.6540	12.3711	25.3270	8.7295	CBL-interacting serine/threonine-protein kinase 11
Cg1g023360	12.2700	24.8727	4.3935	4.0110	9.1912	6.1536	1.6369	20.6226	2.7862	hypothetical protein CISIN_1g020089mg
Cg1g023070	3.7542	4.0074	1.3370	1.5320	1.9878	1.8546	1.8249	6.5845	2.1433	NAC domain-containing protein 7-like
Cg1g022220	1.7123	1.9754	1.1629	4.5781	2.8852	1.4252	2.2649	3.6646	1.8214	copper transport protein ATX1-like
Cg1g021340	4.0565	4.9864	1.0933	1.0583	1.8433	0.9131	0.5073	6.7227	1.1645	probable WRKY transcription factor 40
Cg1g021330	2.8038	1.3977	3.9987	5.1049	4.3486	7.8544	4.9016	3.6047	6.0326	CBL-interacting serine/threonine-protein kinase 5-like
Cg1g019740	12.5147	15.8627	7.0476	6.9199	11.7907	8.1746	7.0550	26.1376	9.6885	bifunctional riboflavin biosynthesis protein RIBA 1, chloroplastic
Cg1g017660	12.6459	10.8077	8.9787	4.6983	4.2159	6.7310	5.0678	10.5683	9.3933	protein LURP-one-related 12-like
Cg1g017510	256.7609	276.2957	112.0195	208.2995	255.4038	103.7310	77.5039	487.8413	86.7758	phospholipase D family protein
Cg1g015020	15.6159	7.8668	22.0389	19.3818	14.7635	23.1115	19.7174	7.3118	20.3920	hypothetical protein CICLE_v10026747mg
Cg1g013930	38.3673	39.7422	23.2881	24.7273	38.1037	22.4771	21.6682	64.5667	17.8233	transcription factor MYBS3
Cg1g013160	3.3225	5.0109	0.1793	0.2273	1.8547	0.3469	0.0518	4.9295	1.1561	Mitochondrial distribution and morphology 12
Cg1g012970	19.3077	13.2261	21.1041	23.9281	10.1202	25.2785	21.4658	8.2831	22.4230	dnaJ homolog subfamily B member 6 isoform X1
Cg1g011620	3.3677	2.5502	4.4085	4.0709	2.9017	6.5995	6.4014	2.6075	6.0521	transcription factor bHLH49 isoform X1
Cg1g010830	59.1834	110.8269	5.7100	4.3236	27.0123	4.1698	1.3002	63.9833	12.4715	protein TIFY 10A-like
Cg1g010820	61.3611	88.3192	7.0827	7.4775	35.7269	7.8920	2.8397	122.4030	25.4419	protein TIFY 10A-like
Cg1g010650	7.1827	6.5965	3.2075	3.0649	3.8299	3.2624	2.2213	9.8125	2.6225	cytochrome P450 71A1-like
Cg1g010370	7.3516	10.6506	5.8680	7.7522	13.0051	6.4960	4.8747	16.9124	4.1454	protein ELF4-LIKE 4
Cg1g009860	56.8955	58.8174	34.9712	58.2295	75.1295	37.3186	39.4105	85.7087	31.5801	NDR1/HIN1-like protein 13
Cg1g008150	16.5572	31.3148	4.8463	10.9132	19.9694	6.2769	5.5018	40.3741	6.4666	No match
Cg1g008020	110.5944	118.0205	60.0189	79.8733	87.3893	51.7660	60.6842	159.5051	59.2746	mitochondrial uncoupling protein 5-like
Cg1g007830	4.1453	4.7567	4.8422	3.9292	4.0135	4.8423	4.6990	5.7230	2.0593	protein NETWORKED 2D
Cg1g007420	5.8038	7.2050	4.2132	4.2119	6.7485	4.0666	4.2359	9.1464	3.8465	transcription factor GTE7-like
Cg1g007160	4.0989	6.6715	1.9784	3.8619	2.7350	1.7799	1.2006	6.3054	0.9318	uncharacterized protein LOC102609906
Cg1g006530	3.7115	4.9831	0.9797	1.5009	3.5448	1.6391	0.6829	5.8962	0.6899	VQ motif-containing protein 9
Cg1g005540	11.7832	13.0128	6.8179	15.7330	12.1436	7.5496	9.7102	21.3081	8.7561	caffeoyl-CoA O-methyltransferase
Cg1g005050	16.4221	25.3016	9.9314	9.1207	13.6759	6.5461	5.5920	23.2604	5.3840	UPF0651 protein YPL107W, mitochondrial
Cg1g004730	8.2036	10.7735	4.8383	6.5261	7.0678	5.6996	5.4357	13.8788	5.5034	disease resistance RPP13-like protein 4
Cg1g003640	9.7066	10.5589	5.9312	6.6344	7.7127	5.8328	5.0931	14.7097	5.9817	UPF0496 protein At2g18630
Cg1g002600	3.8676	4.1006	2.0141	1.3939	2.1654	1.5038	1.2145	6.4858	1.3296	Fungal lipase-like domain containing protein
Cg1g001110	89.0784	144.2254	23.3788	64.1816	93.0131	28.8706	38.4820	236.7608	38.7148	Peptide methionine sulfoxide reductase MrsB
Cg1g000640	1.5457	0.7241	2.7511	1.6545	0.8319	2.7989	1.4219	0.7572	1.0668	Lgl_C domain-containing protein
Cg1g000390	35.4098	46.1768	14.9366	14.9250	24.4176	13.4745	11.3795	70.5019	13.1422	thioredoxin reductase NTRB-like
