# Supplementary material for: Comparative transcriptomic analysis on compatible/incompatible grafts in Citrus
Source: Hortic Res. 2022 Jan 19;9:uhab072. doi: 10.1093/hr/uhab072 (PMC8931943; doi:10.1093/hr/uhab072)
Supplement: Web_Material_uhab072 [file web_material_uhab072.zip › Table S6.pdf]

Table S6 Detail of genes in green module

GeneID	P1			P2			P3			Description
	Hm/Pt	Hm/Cj	Gx/Pt	Hm/Pt	Hm/Cj	Gx/Pt	Hm/Pt	Hm/Cj	Gx/Pt	
Cg6g020720	846.3833	698.8921	1028.9769	200.2936	212.9017	426.8724	68.9057	26.1479	25.0120	vegetative cell wall protein gp1-like isoform X2
Cg9g007970	1709.1158	837.5914	1531.4568	531.1799	493.3296	1129.8567	152.1113	62.0644	62.6751	LPXTG cell wall anchor domain-containing protein
Cg2g033360	666.3497	500.1843	587.5628	71.5515	80.7172	201.3765	24.6458	11.3092	16.1043	21 kDa protein-like
Cg7g021320	331.6587	172.1815	482.1803	160.5188	118.5762	324.5984	101.7661	53.9768	92.0752	expansin-A1
Cg7g003760	768.2217	210.2713	521.5109	284.3331	170.0651	447.4806	327.2136	131.0506	204.4272	uncharacterized protein LOC18043918
Cg5g001530	422.7509	615.6620	393.1253	2530.0542	1456.0394	609.4206	1786.8130	2265.5331	1872.6516	hypothetical protein CUMW_043020
Cg4g009640	528.2377	668.8020	477.6199	2787.9557	1656.7115	1190.8049	1728.8626	3501.5152	3235.0256	metallothionein-like protein type 3
Cg2g009540	242.1702	76.4769	281.7971	55.7052	33.3941	67.0637	45.7125	32.0411	26.1831	chalcone synthase
Cg2g008910	334.4000	378.6488	305.9928	1357.7254	1098.1131	431.8743	1147.4741	1245.6966	1127.0284	glycine-rich protein A3-like
Cg7g003680	287.0999	120.6078	409.4908	70.9347	75.0837	113.1035	31.4204	18.5897	20.7815	expansin-A8
Cg5g001520	418.6677	419.7904	370.6057	2167.6455	1377.7083	614.5122	1436.2248	1707.8961	1291.6942	hypothetical protein CISIN_1g032053mg
Cg8g014260	382.6893	265.7286	411.8468	173.9371	179.2692	278.2285	70.7845	23.2183	52.4161	hypothetical protein CICLE_v10012969mg
Cg4g002100	281.9008	249.4930	282.5636	32.7026	42.5943	79.2011	6.7745	3.2986	2.6799	No match
Cg6g019240	374.3542	158.0826	318.9373	3533.9396	1543.3097	1252.9927	3209.4225	2406.8949	2273.9076	non-specific lipid-transfer protein 2-like
Cg5g043660	473.7495	355.8591	450.4078	47.3223	27.3906	211.4834	8.0605	6.1161	20.3989	14 kDa proline-rich protein DC2.15-like
Cg2g021210	232.5278	125.5961	249.1603	118.2976	74.4347	169.9497	85.2133	35.5496	92.7727	protochlorophyllide reductase, chloroplastic
Cg5g006150	128.9372	57.9798	180.2508	58.0510	53.3619	68.3482	27.8215	17.7481	14.1179	probable pectate lyase 18
Cg9g027520	155.2898	112.8067	122.6245	99.2754	54.8987	81.7139	74.4078	29.6659	60.2274	heat shock protein 83
Cg5g037690	155.6312	146.0339	152.2417	41.0671	50.2945	53.9647	19.7408	13.8959	8.3443	pectinesterase-like
Cg4g018830	71.5664	30.4763	98.3353	10.6752	6.9402	15.7859	10.1338	8.5627	7.3668	transcription factor TT2
Cg2g033130	78.6550	53.6974	93.2877	36.4037	20.1108	37.5780	19.9677	8.7709	15.9746	3-oxo-Delta(4,5)-steroid 5-beta-reductase
Cg1g028670	105.0947	32.8501	114.2483	36.7347	24.6382	63.1927	30.8717	16.7161	26.1426	ribonucleoside-diphosphate reductase small chain
Cg5g005680	72.4819	65.6954	79.5526	288.8805	226.5690	124.9179	222.2614	256.0440	219.5820	hypothetical protein CISIN_1g032713mg
Cg2g026720	76.5710	60.4000	87.0021	10.4962	7.3375	26.8246	4.8676	1.0957	3.3364	subtilisin-like protease SBT5.3
Cg6g002000	38.0387	24.7555	56.1207	10.2800	10.1834	20.2122	10.1554	4.6014	8.7814	aquaporin PIP2-2-like
Cg2g040470	26.1909	23.5547	48.2682	17.8911	12.0462	16.5489	12.5893	6.0076	9.6311	gibberellin 2-beta-dioxygenase 8
Cg5g002280	79.1774	68.8653	73.1412	19.1048	17.9279	21.8684	8.0853	3.6587	5.2930	probable polygalacturonase
Cg1g006460	41.5984	32.7467	47.1321	19.0542	16.0801	28.2441	12.0318	3.4914	9.2963	glucan endo-1,3-beta-glucosidase 12
Cg3g017130	41.2769	22.8407	56.1900	20.9359	26.0242	44.9342	16.5620	11.1196	14.7074	probable receptor-like serine/threonine-protein kinase At5g57670 isoform X1
Cg3g015470	77.8683	57.6551	66.8927	337.8761	181.7286	134.7720	282.4715	288.6741	185.5116	heavy metal-associated isoprenylated plant protein 24
Cg5g003220	27.1023	21.4606	34.3081	14.0112	8.5882	10.7490	3.3244	0.6047	0.6501	transcription factor PRE4-like
Cg5g029170	41.2572	26.4691	41.1150	14.6515	9.7847	17.9386	13.8389	6.5357	7.5664	anthocyanidin 3-O-glucosyltransferase 2-like
Cg7g003300	38.1215	15.6822	54.3246	97.7597	60.5016	63.4900	116.3539	177.4546	119.6210	protein NRT1/ PTR FAMILY 5.1-like
Cg5g002340	31.2421	20.3573	43.8373	15.2581	20.7396	34.0671	18.7022	14.0280	15.1694	uncharacterized protein LOC18048149
Cg8g018270	30.6684	20.6783	38.1039	9.6711	9.7166	18.9471	3.8309	1.7222	2.4247	glucomannan 4-beta-mannosyltransferase 2
Cg9g007480	28.3552	22.7171	38.2202	16.9090	14.9611	32.6954	9.9806	6.3547	13.6835	cellulose synthase-like protein G2
Cg1g026580	30.7327	43.7074	37.7575	13.3137	26.3469	32.1322	14.4304	20.2950	16.0112	cellulose synthase A catalytic subunit 2 [UDP-forming]
Cg5g039320	30.6378	29.9095	33.8041	16.9619	12.8909	24.9032	14.0159	6.7566	16.8959	probable polygalacturonase
Cg9g028710	29.7132	24.7619	32.9577	12.4772	13.2313	11.3772	6.1035	5.2454	2.8926	probable xyloglucan galactosyltransferase GT17
Cg9g002720	42.4048	46.0938	42.0640	120.2917	98.2731	55.9228	100.0229	172.7589	100.6175	late embryogenesis abundant protein Lea5-D
Cg3g024770	38.8539	34.7691	33.1987	148.4080	102.1320	51.5810	108.7657	100.2538	72.4431	probable alpha-amylase 2
Cg6g024880	16.4181	14.1871	18.0662	2.2280	1.3598	1.4473	1.2440	0.4880	0.7186	gibberellin-regulated protein 4
Cg8g021420	12.5939	3.7455	23.7689	2.7466	2.0702	3.0174	0.1423	0.2282	0.3575	chitinase 2-like
Cg2g028300	39.4128	27.4589	40.1634	12.6751	20.3700	21.0596	4.4607	3.0188	2.0323	protein trichome birefringence-like 43
Cg7g010660	26.7741	18.2174	22.6541	104.0303	58.5587	44.9559	88.3863	64.0396	99.1016	bidirectional sugar transporter SWEET12-like
Cg2g037930	24.6423	29.6281	24.0064	79.5238	56.8732	33.8935	68.0111	79.9230	69.9804	protein PHLOEM PROTEIN 2-LIKE A9
Cg8g004580	13.2820	7.4060	17.8532	1.2932	0.9468	2.4906	1.2197	0.5735	0.7550	abscisic acid 8'-hydroxylase 4-like isoform X1
Cg2g003960	17.4573	19.5017	20.6877	7.1189	6.1111	14.1480	6.9759	4.8630	8.2971	zinc finger CCCH domain-containing protein 53 isoform X1
Cg2g037760	23.5850	15.3489	28.9876	7.6899	5.4491	6.2744	3.1688	1.2138	1.5991	protein EXORDIUM-like 7
Cg2g025890	24.1807	20.5900	28.1366	4.4547	5.7682	12.8720	1.4767	1.7606	1.7273	aspartyl protease family protein At5g10770
Cg9g002760	8.7254	2.4389	15.3110	1.4363	0.6653	2.1141	1.9058	0.7901	1.6319	UDP-glucuronic acid decarboxylase 2-like
Cg2g019370	22.1558	11.9419	21.3503	4.5513	3.0132	8.8512	1.6798	0.9200	1.7229	GDSL esterase/lipase At2g42990-like
Cg6g019970	17.5398	11.5269	20.4538	12.2132	12.3080	16.0159	5.5803	2.7101	4.4743	protein trichome birefringence-like 19
Cg7g020180	10.7937	9.6525	14.5009	3.4164	4.8430	6.6518	2.6536	2.5990	2.7171	TORTIFOLIA1-like protein 4 isoform X1
Cg2g031810	17.5188	15.5848	17.8499	7.7576	8.4637	8.3639	6.1135	3.1353	1.9548	bidirectional sugar transporter SWEET16
Cg6g017290	14.9025	10.6277	17.7937	7.5476	7.5886	14.5498	10.5996	8.6954	10.7873	E3 ubiquitin-protein ligase At4g11680-like isoform X1
Cg9g024230	14.4198	17.8613	15.7967	2.6652	2.3828	6.6187	1.5183	0.9571	1.1261	probably inactive leucine-rich repeat receptor-like protein kinase At3g28040
Cg5g042970	11.2584	10.9374	14.8064	6.2058	7.8515	11.9399	6.4484	5.6664	7.0782	polygalacturonate 4-alpha-galacturonosyltransferase
Cg6g017010	10.0217	6.0796	10.5592	2.1775	0.8485	2.1548	2.6427	2.1754	1.9954	Dihydrofolate reductase
Cg5g017310	16.1734	36.9946	15.1673	34.4313	42.7863	26.9718	38.3550	48.9361	45.8610	malonyl-CoA:anthocyanidin 5-O-glucoside-6''-O-malonyltransferase-like
Cg5g016100	4.9046	5.8699	10.4297	2.5747	2.8046	2.3918	1.2760	0.3461	1.0554	Heat stress transcription factor B-4b, putative
Cg5g000080	8.1832	6.4843	10.4132	5.1511	5.0455	6.5959	3.4585	1.5941	1.9064	RING-H2 finger protein ATL79-like
Cg2g036350	9.6922	6.6788	11.0120	3.1876	3.2488	5.9674	5.0968	3.6307	3.9009	CLAVATA3/ESR (CLE)-related protein 27-like
Cg7g022450	7.6201	5.0870	11.1629	41.1099	26.4252	13.7740	26.0121	26.6778	16.9638	uncharacterized protein LOC18045490
Cg7g019530	14.8670	14.7817	18.2731	6.1396	8.1410	15.1587	4.0062	2.4402	2.0280	probable xyloglucan endotransglucosylase/hydrolase protein 33
Cg5g036570	6.7869	3.0020	8.4777	1.0909	0.8033	1.7401	1.7057	1.4429	1.3946	bifunctional 3-dehydroquinate dehydratase/shikimate dehydrogenase, chloroplastic-like
Cg7g014870	10.0665	6.0049	9.3007	2.1586	1.8630	4.4095	2.0932	1.2699	1.9409	leucoanthocyanidin reductase-like
Cg7g015320	10.9232	9.9083	11.9906	3.7340	4.2185	9.4422	2.8395	1.6772	2.4390	protein STRICTOSIDINE SYNTHASE-LIKE 10
Cg5g029700	7.7653	9.7402	7.9695	22.8324	16.8827	9.9675	21.1714	23.8871	21.2371	S-norcoclaurine synthase-like
Cg5g016380	4.7488	1.1227	7.1262	0.9460	0.8109	4.5426	0.8274	0.3098	0.4175	hypothetical protein COLO4_17395
Cg2g046580	9.1308	3.6532	8.8584	18.6916	10.9869	12.9878	23.8752	20.8148	17.2677	U-box domain-containing protein 35
Cg4g001710	11.6012	5.0371	9.3512	15.5622	9.8817	15.3417	20.6683	24.3455	21.8883	coumarin 8-geranyltransferase 1b, chloroplastic
Cg9g011140	8.4118	2.2360	5.1901	2.0843	1.4337	3.3110	1.3006	0.2950	0.2522	hypothetical protein CICLE_v10005919mg
Cg7g002390	5.4331	4.5533	7.3185	3.6661	5.9054	7.4509	4.3114	4.3554	4.0781	probable methyltransferase PMT23
Cg2g035860	7.2982	8.9156	6.9777	14.6984	13.2373	6.9797	13.4899	14.2268	13.0972	thioredoxin-like 3-3
Cg6g003200	5.3270	3.9828	5.6065	2.7557	2.1762	1.5496	1.3378	0.6236	0.8817	suppressor protein SRP40 isoform X1
Cg2g039610	5.1493	5.3744	5.3130	1.8061	1.1470	1.5804	0.9601	0.4177	0.6211	protein kinase PINOID-like
Cg9g008050	5.5489	4.4969	5.1586	1.8857	2.7914	3.1070	0.4958	0.2295	0.0308	hypothetical protein CICLE_v10006057mg
Cg2g026730	6.6146	4.9415	7.4831	1.4282	0.9658	3.2669	0.7023	0.1397	0.4834	subtilisin-like protease SBT5.3
Cg4g017070	4.2239	3.7054	5.2095	1.3454	1.7744	2.6659	0.7234	0.6138	0.8004	probable galactinol--sucrose galactosyltransferase 1
Cg7g003360	3.2479	2.0680	4.5142	0.5144	0.4823	1.3106	0.0822	0.0289	0.0000	putative Structural maintenance of chromosomes protein 1
Cg5g033320	3.2140	2.7353	3.9209	2.6631	2.6261	1.5564	0.9506	0.3048	0.3210	hypothetical protein CISIN_1g047327mg
Cg2g040950	4.5543	2.1918	4.8694	6.5565	5.0131	7.7988	9.4407	12.2625	12.2531	transcription factor bHLH112 isoform X2
Cg8g008680	3.4395	3.5179	3.9079	1.3620	1.4165	0.9559	0.9003	0.4129	0.5747	endoglucanase 24-like
Cg6g003460	2.9558	2.3032	4.4239	1.1615	1.6735	2.7300	1.3470	0.6188	1.1025	No match
Cg4g021600	4.3137	2.7540	5.4626	2.2422	2.0820	4.4898	2.6149	2.0036	2.7655	homogentisate phytyltransferase 1, chloroplastic isoform X1
Cg6g014130	3.6116	2.4207	5.1639	10.0688	4.8815	4.3668	11.2440	10.7639	11.2551	No match
Cg2g005370	5.3642	6.5870	5.3054	3.1337	5.2046	6.3689	2.5277	1.9006	2.8793	blue copper protein-like
Cg3g025240	1.9555	2.3988	2.9333	0.9360	0.8726	0.8060	0.4762	0.2019	0.2565	cyclin-B2-4-like isoform X1
Cg4g002510	3.0898	1.9297	4.6403	0.3259	0.3772	1.8080	0.0664	0.3692	0.4391	lysM domain receptor-like kinase 3
Cg9g007130	3.3852	3.2772	4.3794	2.8450	2.2991	2.3094	1.0275	0.5264	0.2378	protein ACCELERATED CELL DEATH 6-like
CgUng005420	4.1496	2.3491	4.1688	1.5219	1.2564	0.7965	0.1466	0.0000	0.1113	auxin-responsive protein SAUR23-like
Cg2g022900	2.2320	2.8310	5.3800	0.9636	1.4503	1.9691	2.0375	1.6731	2.4921	No match
Cg1g007280	3.0445	2.7728	3.9846	1.4464	1.4441	1.8299	0.8324	0.4209	0.3592	kinesin-like protein KIN-8B isoform X1
Cg4g014240	2.0026	2.7215	3.1598	0.7728	0.8337	1.5496	0.9569	0.4731	1.4018	LRR receptor-like serine/threonine-protein kinase
Cg5g042690	3.7963	3.4320	3.3198	1.3739	1.4769	1.4888	1.0723	0.4921	0.7291	plant UBX domain-containing protein 8
Cg5g038140	3.2202	3.1048	3.3140	1.0078	0.9978	2.4254	0.8252	1.0881	1.0095	Fungal lipase-like domain containing protein
Cg5g043940	3.1281	1.2276	4.0101	1.1309	2.6629	3.9264	0.5242	0.1717	0.1958	metalloendoproteinase 4-MMP-like
Cg7g016460	3.1500	2.0936	3.3667	28.2896	12.5203	9.7906	34.3863	28.7194	17.5639	cytochrome P450 78A5-like
Cg2g040820	6.1395	2.5936	5.8395	2.8862	1.3651	3.2586	1.6614	0.5421	2.0203	alpha-farnesene synthase
Cg5g034730	4.7878	3.4006	4.2928	1.1523	1.0312	2.6443	1.6163	1.0760	2.3001	pyruvate kinase isozyme A, chloroplastic

Cg2g041650	3.3019	1.0123	3.4016	6.5567	5.9845	4.7039	8.2073	8.7196	6.3305	NAC transcription factor 25
Cg3g000380	5.0221	3.9084	4.3985	9.1347	7.2560	4.2565	8.8863	10.0692	9.3446	LIM domain-containing protein WLIM2b
Cg1g004720	2.1970	2.8562	2.5075	7.1171	5.6871	3.0430	5.3549	7.3478	6.6849	AAA-ATPase At2g18193-like
Cg8g006200	5.3531	4.7453	4.5061	2.6781	3.0177	3.1896	1.6828	0.8700	0.6583	probable 1-acyl-sn-glycerol-3-phosphate acyltransferase 5
Cg9g019740	3.1210	1.8052	3.7646	1.1712	0.8970	3.1906	0.9030	0.6140	1.1066	receptor-like protein EIX2
Cg5g039220	3.0920	1.5095	2.7810	1.3451	1.5069	2.0174	0.4771	0.2595	0.2188	acyl transferase 4
Cg9g020330	2.9197	1.4130	3.0047	23.2104	14.8753	12.9301	30.1350	31.2394	23.4421	ABC transporter G family member 15-like isoform X1
Cg5g000340	2.4815	3.1498	3.9138	1.4285	2.2939	3.6988	1.1598	0.9360	0.9597	NAC domain-containing protein 43-like
Cg8g024160	2.0894	1.5738	2.1348	16.9428	9.2912	7.2515	14.8208	13.2581	12.1447	phosphoinositide phospholipase C 4-like
Cg3g008080	0.7679	1.2190	1.4964	1.8818	1.2848	0.8332	2.3207	2.2123	2.3844	cytochrome P450 CYP82D47-like
CgUng000240	2.1230	2.0506	1.6492	6.0766	6.7222	2.4914	6.2821	6.9663	3.6524	beta-amyrin 11-oxidase-like
Cg5g011680	1.6228	1.3645	2.0554	0.8846	1.1981	2.4400	1.0028	0.7499	0.8360	protein COBRA-like
Cg8g010780	1.4532	1.6135	1.2853	7.6399	4.8794	3.0978	4.0160	8.5228	6.9231	HRAS-like suppressor 3 isoform X2
Cg3g025970	1.7986	1.7288	1.6758	4.3725	4.2142	2.0000	4.2939	6.6045	4.3844	PLATZ transcription factor family protein isoform 3
Cg4g022370	1.8096	1.6331	1.9900	0.5540	0.4203	0.7448	0.2324	0.0883	0.0878	probable 1-deoxy-D-xylulose-5-phosphate synthase 2, chloroplastic
Cg7g014650	1.9681	0.7426	1.5740	0.5071	0.9657	2.3261	0.4179	0.4512	0.5938	probable carboxylesterase 6
Cg6g004880	2.6121	1.6695	3.4858	0.9632	2.0471	2.5985	0.2988	0.1955	0.0522	Multicopper oxidase, type 1
Cg2g034080	1.8110	1.6467	2.2667	0.7812	1.3956	0.8457	0.2282	0.1878	0.0124	putative fasciclin-like arabinogalactan protein 20
Cg1g011580	1.0884	0.9631	1.0101	5.0278	3.1280	2.2761	4.0231	4.6762	3.3500	transcription factor bHLH111
Cg2g036370	1.7607	3.6440	1.4995	3.9236	4.9478	2.0301	3.0103	4.4412	3.4570	zinc finger protein CONSTANS-LIKE 16
Cg7g022000	0.4939	0.6876	0.9822	11.8902	5.0436	4.7137	14.2036	15.5919	20.3902	auxin-responsive protein SAUR32-like
CgUng003260	0.6845	1.0058	1.4112	0.3079	0.3967	0.6668	0.0868	0.0000	0.0350	SNW/SKI-interacting protein-like
Cg1g013950	3.9226	1.0167	2.3166	0.5181	0.2153	0.9360	0.0560	0.0528	0.0316	No match
Cg2g030210	0.9235	0.2509	1.0580	4.6568	2.5081	1.5618	1.9721	3.4184	3.5809	protein DMP10
Cg5g027500	1.9946	1.1996	1.2861	9.7397	6.0489	3.7191	12.0754	11.3400	7.8403	heparan-alpha-glucosaminide N-acetyltransferase-like
Cg6g012900	0.4061	1.3224	0.6128	2.7065	1.9990	1.3323	2.5371	2.4018	3.5093	putative Adipose-regulatory protein
Cg9g012440	0.5684	2.0532	0.6600	2.7511	2.7944	1.2164	3.3437	2.6869	2.6194	1-aminocyclopropane-1-carboxylate oxidase homolog 1-like isoform X1
Cg2g030070	0.7112	0.5705	0.7758	2.3405	1.7537	0.9259	1.4821	1.7436	1.5150	probable disease resistance protein At4g27220
Cg2g015270	0.6287	0.5708	0.6072	2.0247	1.9098	0.7268	1.6802	2.8627	1.4637	E3 ubiquitin-protein like
Cg1g009400	0.3583	0.6465	0.5001	1.7094	0.9816	0.6474	1.0395	1.1064	1.5687	peroxidase 21-like
Cg3g013430	0.5867	0.4781	0.4987	8.3674	7.3029	2.9967	9.4735	10.3163	10.6869	protein DETOXIFICATION 48
CgUng015510	0.9696	0.1406	0.6511	11.4887	2.8038	3.4753	10.9739	9.0416	7.5465	disease resistance protein SUMM2-like
Cg6g005030	0.4901	0.1433	0.4875	1.7541	1.3307	0.7667	2.1534	2.2479	2.0201	subtilisin inhibitor-like
Cg9g026350	0.2399	0.2641	0.3263	1.3408	1.2073	0.4701	0.7408	1.1916	1.0371	protein DETOXIFICATION 49
Cg3g018290	0.1496	0.0000	0.1974	1.7214	1.1568	0.5714	1.7578	1.7935	1.5994	tRNA-dihydrouridine(20) synthase isoform X1
Cg3g009570	1.2154	1.3846	0.9177	3.9265	3.2113	1.4606	3.4855	4.6872	4.2631	copper transport protein ATX1-like
Cg7g012330	0.4262	0.3254	0.2973	1.8745	0.7480	0.9459	3.0815	2.6230	2.7354	TLC domain-containing protein 4-like
Cg1g002190	0.2334	0.3839	0.1983	1.5305	1.0926	0.7100	1.1940	1.2525	1.0259	hypothetical protein CUMW_182150
Cg1g001340	0.1253	0.0270	0.1236	9.4381	4.0672	3.8648	14.3739	7.8230	11.9176	GDSL esterase/lipase EXL3-like
Cg8g005210	0.1758	0.0812	0.0974	4.5104	1.9981	1.3858	3.5541	2.6758	2.2778	uncharacterized protein LOC107177262
Cg1g024710	0.5492	0.2755	0.1837	3.6844	0.7387	1.0325	4.8184	4.5187	5.5093	probable LRR receptor-like serine/threonine-protein kinase At3g47570 isoform X2
Cg9g016930	0.0959	0.1907	0.0882	1.5986	0.8378	0.7471	2.2329	1.7644	1.5199	L-idonate 5-dehydrogenase-like
