# Supplementary material for: Comparative transcriptomic analysis on compatible/incompatible grafts in Citrus
Source: Hortic Res. 2022 Jan 19;9:uhab072. doi: 10.1093/hr/uhab072 (PMC8931943; doi:10.1093/hr/uhab072)
Supplement: Web_Material_uhab072 [file web_material_uhab072.zip › Table S7.pdf]

Table S7. Detail of differentially expressed genes between different rootstocks

GeneID	P1			P2			P3			Description
	Hm/Pt	Hm/Cj	Gx/Pt	Hm/Pt	Hm/Cj	Gx/Pt	Hm/Pt	Hm/Cj	Gx/Pt	
Cg8g022750	2.2172	1.0735	1.6864	3.2360	2.6767	1.8280	2.1891	2.9405	1.6250	zinc transporter 2
Cg9g029160	0.5552	1.2354	0.8628	0.9793	0.4120	0.7684	0.5506	1.1226	0.9320	zinc transporter 1-like
Cg6g017450	0.4683	0.6323	0.3394	0.5181	0.5141	1.2693	1.8889	0.8305	1.5368	zinc finger protein GIS-like
Cg2g022110	20.7689	25.5858	18.8349	18.4155	26.7237	18.0087	18.2652	37.7448	23.1599	zinc finger protein CONSTANS-LIKE 1-like
Cg3g025670	4.6249	2.1275	3.7115	2.4860	4.4309	6.2598	1.1432	0.5093	0.3671	xylem cysteine proteinase 2
Cg2g015280	2.0527	0.7562	1.1217	1.8328	3.0841	2.1071	1.8069	3.7925	2.5590	Wound-responsive family protein
Cg3g001750	1.7304	0.6536	1.2659	1.1224	1.0763	1.2139	0.2729	0.0819	0.1108	WEB family protein At3g51220
Cg9g024970	0.2402	0.4840	0.0993	0.3087	0.2806	0.0856	0.2124	0.7155	0.3755	wall-associated receptor kinase-like 2
Cg2g024270	0.3101	0.2592	0.2468	0.7281	0.6153	0.4487	0.3772	0.8230	0.4363	wall-associated receptor kinase-like 2
Cg2g044910	12.5007	18.3056	10.4304	11.1649	17.6750	5.6716	6.5882	14.7443	7.6496	VQ motif-containing protein 1-like
Cg7g005850	0.6295	0.5647	0.9131	0.6159	1.2207	0.9429	1.0575	1.4405	1.1217	uncharacterized protein YKR070W
Cg4g017460	2.6949	2.4664	3.1770	6.1878	2.9971	2.8905	5.3318	3.8697	4.7267	uncharacterized protein LOC18054812 isoform X1
Cg2g021510	2.5892	5.5212	3.3899	1.0736	0.8926	1.4015	0.7744	1.2814	0.5657	uncharacterized protein LOC18054182
Cg4g009460	0.4178	0.3469	0.3170	0.2762	0.6148	0.5147	0.5370	2.5793	0.8385	uncharacterized protein LOC18053836
Cg7g003760	768.2217	210.2713	521.5109	284.3331	170.0651	447.4806	327.2136	131.0506	204.4272	uncharacterized protein LOC18043918
Cg6g019170	0.6996	0.7279	0.4593	1.2157	0.8884	0.4427	0.3262	2.1983	0.6489	uncharacterized protein LOC107177559
Cg2g008970	5.3291	6.5131	4.8497	4.5413	5.8096	5.2722	3.1601	7.3248	5.8892	uncharacterized protein LOC107175629
Cg1g014100	30.3712	22.8206	33.1572	32.5070	15.4164	28.6696	26.4787	18.0259	25.8621	uncharacterized protein LOC102630687 isoform X2
Cg5g032690	6.8249	2.9410	8.0179	28.1700	11.7230	22.6117	58.0293	39.5242	51.6803	uncharacterized protein LOC102628841
Cg8g003740	5.2309	4.0562	4.5343	12.6332	5.2636	6.4707	6.2730	8.5468	6.9444	uncharacterized protein LOC102627881
Cg8g003340	1.1571	2.6262	1.2093	0.1825	0.2063	0.3447	0.3964	6.0239	1.2299	uncharacterized protein LOC102616231
Cg1g011760	1.2753	1.4565	1.5679	2.2626	1.0626	1.2768	1.8938	2.7740	2.4150	uncharacterized protein LOC102614795
Cg6g003540	5.6449	5.6524	1.3259	1.0749	2.7615	0.7259	0.6220	4.6648	1.5898	uncharacterized acetyltransferase At3g50280-like
Cg6g003520	0.9692	1.2312	0.2326	0.1383	0.5056	0.0439	0.0816	0.6100	0.1550	uncharacterized acetyltransferase At3g50280-like
Cg8g023280	0.6534	0.1848	0.3582	0.4597	0.2295	0.3020	0.0915	0.0496	0.1000	UDP-Glycosyltransferase superfamily protein
CgUng004780	3.6863	2.3450	2.5162	8.9132	9.2691	6.7000	7.4860	18.7725	10.2350	UDP-glycosyltransferase 73C6
Cg2g012130	4.5920	5.1515	4.2483	5.3988	7.4831	4.2237	4.3493	8.7515	6.1549	UDP-glycosyltransferase 73C3
Cg3g007550	0.2069	0.1220	0.2648	0.3971	0.3779	0.0969	0.5639	0.1304	0.2563	ubiquitin carboxyl-terminal hydrolase 12-like
Cg5g005400	4.9018	2.3365	3.5373	4.5131	2.3701	3.7857	6.6699	4.8264	5.3916	tryptophan aminotransferase-related protein 2 isoform X1
Cg1g023660	3.4833	1.7762	3.4520	2.5496	2.7723	2.9321	1.2400	0.6478	0.4873	transcription repressor OFP17
Cg3g010310	1.5017	3.1803	2.6538	1.5100	1.4422	2.2416	1.3237	1.4024	1.5592	transcription factor UNE12 isoform X2
Cg8g013710	0.8435	1.0062	0.5687	1.2353	1.0346	0.6606	1.1724	3.8495	2.0571	transcription factor SRM1
Cg2g007230	1.7013	1.1855	0.1086	0.1820	5.6171	0.3145	0.3253	3.6871	1.6583	transcription factor MYB15-like
Cg5g034000	2.9967	1.6760	1.0757	1.1655	1.8751	0.7506	1.0009	8.9220	6.0149	transcription factor MYB14-like
Cg9g000740	0.4913	0.8321	0.0847	0.0514	0.2003	0.0212	0.0180	0.7331	0.0874	transcription factor MYB108-like
Cg5g034360	0.8607	0.8362	0.2602	0.5384	1.0518	0.3167	0.1565	1.0974	0.3597	transcription factor bHLH3-like
Cg4g016660	0.6409	0.3064	0.4965	0.1876	0.0870	0.6682	0.0807	0.3426	0.1480	TMV resistance protein N-like
Cg7g013870	4.3097	2.1899	4.1813	5.6583	2.9218	6.4836	7.9772	4.8200	6.0987	thioredoxin-like protein YLS8 isoform X2
Cg6g004960	3.6584	7.4616	4.7092	3.7206	4.7449	1.9509	2.2007	1.1045	1.0406	thaumatin-like protein
Cg6g003200	5.3270	3.9828	5.6065	2.7557	2.1762	1.5496	1.3378	0.6236	0.8817	suppressor protein SRP40 isoform X1
Cg3g015430	10.1172	22.8017	14.0729	12.7229	20.8645	17.0700	10.0289	8.7454	17.5340	sulfate transporter 1.3
Cg2g026830	1.7174	1.0758	1.8248	0.9988	1.5335	2.7902	1.8247	0.5385	0.9937	subtilisin-like protease SBT5.3
Cg2g041950	1.0252	1.5121	0.7684	0.6064	0.6281	0.2653	0.3732	0.1035	0.2288	stem-specific protein TSJT1-like
Cg4g013660	41.4522	61.3457	47.0476	17.2418	13.1640	7.6475	4.0099	1.6682	1.6153	sodium/potassium/calcium exchanger 1
Cg8g013560	0.0593	0.2614	0.2852	0.3011	0.5700	0.1346	0.1603	1.0154	0.3800	small RNA-binding protein 11, chloroplastic-like
Cg2g012670	3.9965	1.6212	2.1028	2.5987	1.0352	2.1923	3.4736	1.1610	3.0559	small heat shock protein, chloroplastic-like
Cg5g019450	37.2588	23.8793	20.4875	48.1247	32.1268	22.3919	85.6884	34.2380	60.5239	small heat shock protein, chloroplastic
Cg4g023550	0.5504	0.5320	0.6684	0.6778	0.8846	1.3834	1.0343	2.1223	1.4410	shikimate O-hydroxycinnamoyltransferase-like
Cg6g023660	0.5135	0.2549	0.3503	0.3752	0.2445	0.1792	0.2989	0.2158	0.4801	serine/threonine-protein kinase D6PK
Cg7g006290	26.8807	35.5253	21.8184	28.3163	29.4697	21.4346	20.1906	40.2937	24.0834	Serine/threonine-protein kinase bur1
Cg4g006370	0.7442	1.3179	0.4057	0.7071	0.6455	0.1723	0.2314	1.2366	0.5648	serine hydroxymethyltransferase 3, chloroplastic-like
Cg7g022850	0.4521	0.9357	0.5785	1.5137	1.8684	1.5280	1.5215	0.8479	1.5638	serine carboxypeptidase-like 40
Cg2g006000	43.1645	68.5724	23.1584	22.2345	30.8926	24.0703	23.6291	46.6246	32.3066	seed maturation-like protein
Cg9g000390	61.0406	268.8760	78.6476	46.0002	175.8337	95.6705	29.3054	42.4810	68.6303	S-adenosylmethionine synthase 2
Cg5g013920	142.9545	185.9098	109.6188	88.1102	114.2802	87.5893	69.9013	140.4391	75.2884	S-adenosylmethionine synthase 1
Cg5g041400	0.1705	0.4238	0.3529	0.4107	0.2124	0.3610	0.5835	0.4816	0.2511	S-adenosyl-L-methionine-dependent methyltransferases superfamily protein
Cg5g033100	16.3174	7.3187	9.7882	7.8386	6.9782	7.5535	2.6848	7.9126	2.4904	RmlC-like jelly roll fold
Cg5g000080	8.1832	6.4843	10.4132	5.1511	5.0455	6.5959	3.4585	1.5941	1.9064	RING-H2 finger protein ATL79-like
Cg5g037610	14.2229	8.7675	11.2110	13.4780	8.1946	13.6015	7.3003	19.2111	13.7061	RING-H2 finger protein ATL64-like
Cg1g019680	0.3926	0.4898	0.3791	1.1877	1.1822	0.5899	1.7856	4.4231	3.2145	RING-H2 finger protein ATL54-like
Cg4g022690	8.5645	7.0961	9.8718	8.7768	5.0255	9.3141	7.8441	3.7847	7.3098	RING-H2 finger protein ATL13
Cg8g024000	7.7149	4.6356	9.9315	41.1102	14.4220	21.5269	49.1690	22.4098	36.6828	RING/U-box superfamily protein isoform 2
Cg9g004590	5.5862	3.9957	7.7322	4.1598	3.6733	4.3705	3.4487	1.4993	2.5100	RING zinc finger protein
CgUng016870	0.3299	0.9015	0.4442	0.8987	0.6473	0.4202	0.4700	0.6842	0.8118	ribosomal protein L14
Cg9g019550	0.2270	0.3225	0.2824	0.8963	0.2951	0.1768	0.4914	0.2132	0.3522	rho guanine nucleotide exchange factor 8-like
Cg1g008100	0.9535	0.4393	0.8901	0.4783	0.4968	0.4847	0.1330	0.2153	0.0680	retrovirus-related Pol polyprotein from transposon TNT 1-94
Cg7g011630	3.1901	7.3657	2.9437	3.8621	4.3549	2.8613	3.6506	1.6220	3.1475	respiratory burst oxidase homolog protein C
Cg9g018690	1.9030	0.6846	1.3667	3.5187	3.5859	7.0041	6.8825	21.1800	9.2051	receptor-like protein EIX2
Cg9g018760	0.0640	0.0580	0.1039	0.2229	0.2515	0.4794	0.4582	0.9210	0.6031	receptor-like protein EIX2
Cg2g023850	0.1406	0.0166	0.6407	0.7327	0.4384	1.2615	1.0492	0.3802	0.7958	receptor-like protein EIX2
Cg5g016150	1.0428	2.2344	0.4963	0.8118	1.1594	0.1082	0.3592	1.8891	0.7187	receptor-like protein 9DC3
Cg5g026260	7.0535	10.1689	5.2862	7.7760	7.8730	5.6944	6.1169	12.3785	6.3461	putative UPF0481 protein At3g02645
Cg7g002810	14.1545	44.2230	24.4765	22.3022	68.4488	37.7665	18.7269	19.1255	88.1326	putative sodium-coupled neutral amino acid transporter 7
Cg7g021930	1.7173	1.2363	1.4346	0.3878	0.8970	0.4618	0.1064	0.4379	0.1667	putative SNAP25 homologous protein SNAP30
Cg4g010810	11.4041	14.7288	9.5757	11.7524	13.0869	10.3282	10.5067	24.9582	13.0144	putative serine/threonine-protein kinase
Cg6g005510	0.3550	0.7272	0.1663	0.2847	0.2696	0.1674	0.2700	0.7739	0.4755	putative receptor protein kinase ZmPK1
Cg9g022920	0.6872	0.5097	0.5699	1.2362	0.5990	0.5328	1.3546	0.3528	0.7739	putative lipase ROG1
Cg1g006240	22.9853	19.7696	30.6237	22.1025	14.8590	20.3945	15.0505	7.1155	11.5099	putative Late embryoproteinsis abundant protein group 8 protein
Cg6g019270	0.3551	0.1031	0.2667	0.6780	0.3594	1.0391	0.7230	1.4987	0.8515	putative glutamine amidotransferase GAT1_2.1
Cg8g017510	17.1211	10.5612	24.0301	34.8978	15.2330	28.5996	33.2190	22.4983	34.2692	putative alpha/Beta hydrolase fold protein
Cg6g012900	0.4061	1.3224	0.6128	2.7065	1.9990	1.3323	2.5371	2.4018	3.5093	putative Adipose-regulatory protein
Cg5g027400	3.8836	1.5593	7.6626	0.4190	0.2454	0.3882	0.3007	0.1218	0.1335	protodermal factor 1
Cg4g023800	0.3297	0.5135	0.5432	0.2731	0.1098	0.2339	0.2138	0.0294	0.0494	proteoglycan 4-like isoform X2
Cg6g025670	2.2701	2.5865	2.8433	0.8352	0.7126	0.6887	0.3541	0.1141	0.1646	protein WVD2-like 4 isoform X1
Cg6g009760	1.5987	3.4018	2.2161	1.6396	2.4574	2.0607	1.0062	0.9361	1.1236	protein trichome birefringence-like 34
Cg6g019970	17.5398	11.5269	20.4538	12.2132	12.3080	16.0159	5.5803	2.7101	4.4743	protein trichome birefringence-like 19
Cg9g025660	0.3405	1.0323	0.4078	0.3414	0.6125	0.4048	0.4591	1.0018	0.5234	protein TPX2 isoform X1
Cg6g008960	0.9081	1.0535	1.0449	0.6182	1.5591	1.0549	0.4821	0.5702	0.5413	protein TORNADO 2
Cg4g017780	284.0103	274.9813	91.8158	62.1159	151.3058	56.7639	41.6621	356.2121	157.4081	protein TIFY 10A
Cg9g011680	16.0504	40.9434	23.7605	25.5714	43.2437	21.5314	14.6836	7.5150	28.3442	protein SULFUR DEFICIENCY-INDUCED 2
Cg5g032660	7.9846	46.7222	23.0284	9.9508	58.6902	20.1532	4.6947	5.5959	19.4269	protein SULFUR DEFICIENCY-INDUCED 1-like
Cg9g003190	12.4341	7.5895	13.2885	55.4002	18.7345	28.0177	40.0448	18.2115	37.6650	protein SRG1
Cg5g010720	5.4313	2.5816	4.9071	4.9792	4.9469	5.0911	8.9030	6.9517	35.7886	protein SIEVE ELEMENT OCCLUSION B-like
Cg3g020290	29.5982	24.3802	27.4641	25.3876	29.5964	30.5876	26.1582	55.6273	28.0748	protein SENSITIVE TO PROTON RHIZOTOXICITY 1
Cg5g037520	484.5107	402.1270	249.9885	621.5496	634.0377	533.7966	449.2123	1161.2719	592.2675	protein SENESCENCE-ASSOCIATED GENE 21, mitochondrial-like
Cg4g001690	2.4747	5.3216	2.6268	1.4326	1.9524	1.4014	0.4205	1.5203	0.7220	protein PPLZ02-like
Cg2g033320	83.8664	47.2327	90.0234	112.5220	52.2952	81.3891	67.4766	45.5647	61.7453	protein PLANT CADMIUM RESISTANCE 2-like
Cg1g007940	0.1989	1.0659	0.6063	0.0442	0.0000	0.1731	0.1524	0.0906	0.0000	protein PELPK1
Cg2g012510	0.6619	0.2142	0.4019	1.4376	0.9214	0.7257	1.3434	4.4270	1.1963	protein NUCLEAR FUSION DEFECTIVE 4-like
Cg8g019360	8.0603	11.5145	5.3662	4.5983	5.8810	5.9743	4.3368	11.0366	6.8495	protein NUCLEAR FUSION DEFECTIVE 4
Cg6g006390	0.6060	0.7489	0.6768	0.4632	0.9664	0.7494	0.3747	0.5239	0.3972	protein NRT1/ PTR FAMILY 8.1-like
Cg9g009120	0.2237	0.2256	0.1415	0.5140	0.1865	0.2165	0.3587	0.8532	0.5139	protein NRT1/ PTR FAMILY 5.2
Cg7g014500	45.9964	21.9336	62.4679	72.8973	32.8964	63.5682	70.0284	45.0066	44.5036	protein NRT1/ PTR FAMILY 3.1
Cg7g014510	186.3601	61.3668	245.6500	216.8044	86.5611	220.5215	186.1514	69.0048	95.8884	protein NRT1/ PTR FAMILY 3.1
Cg2g040530	0.4825	1.3301	0.9581	1.7626	0.9306	1.5274	0.7062	0.3805	0.5539	protein NDR1-like
Cg7g002770	1.2338	1.9861	0.9035	1.1576	1.2338	0.5263	0.6737	0.2990	0.3526	protein LURP-one-related 14
Cg1g017660	12.6459	10.8077	8.9787	4.6983	4.2159	6.7310	5.0678	10.5683	9.3933	protein LURP-one-related 12-like
Cg6g010080	15.2632	7.5056	19.2859	38.2951	18.2576	32.0139	38.8822	30.6263	67.5505	protein LURP-one-related 10-like
Cg2g039610	5.1493	5.3744	5.3130	1.8061	1.1470	1.5804	0.9601	0.4177	0.6211	protein kinase PINOID-like
Cg6g000120	3.7326	1.4793	2.8586	9.5839	8.5705	4.3687	4.6107	7.9290	4.6513	protein HEADING DATE 3A

Cg6g020180	3.9304	4.6372	4.7894	10.0215	6.8395	5.6218	6.1499	2.8162	4.4132	protein GLUTAMINE DUMPER 6
Cg4g022420	5.6485	9.3293	9.9962	24.1010	16.1548	10.6088	19.0091	9.1067	16.7221	protein GLUTAMINE DUMPER 3
Cg9g024480	0.7174	0.7081	0.4780	0.5977	0.7268	0.4334	0.5772	1.2790	0.7008	protein FLOURY 1-like
Cg9g004990	48.7927	61.1450	32.3848	17.7410	36.4766	18.8916	11.4737	20.6555	10.8008	protein FANTASTIC FOUR 3-like
Cg2g037760	23.5850	15.3489	28.9876	7.6899	5.4491	6.2744	3.1688	1.2138	1.5991	protein EXORDIUM-like 7
Cg8g005550	11.8478	50.9779	37.3924	7.1195	41.9685	25.4819	3.7108	7.7466	18.2259	protein DMR6-LIKE OXYGENASE 2
Cg9g003920	3.3315	1.4150	2.8023	2.3067	2.5096	5.4909	2.2712	1.6423	6.1111	protein DMP2
Cg5g009140	0.9228	0.8371	1.2521	2.4921	1.1382	2.3150	1.7880	1.0824	1.4358	protein DETOXIFICATION 56
Cg9g019350	9.4243	12.2056	8.1180	5.0781	7.5833	4.9420	4.4811	9.0328	5.5326	protein CfxQ homolog
Cg5g000380	3.1690	6.9460	3.6562	1.4265	1.5163	1.4112	0.8951	0.8410	0.6518	protein BRANCHLESS TRICHOME
Cg3g017930	1.4972	0.6012	1.3503	0.3611	0.6109	4.5695	0.7865	0.2080	0.3497	proline-rich receptor-like protein kinase PERK2
Cg5g036360	15.0882	31.0110	17.7084	17.0755	23.4506	14.0258	13.3225	7.0405	7.2048	probably inactive leucine-rich repeat receptor-like protein kinase IMK2
Cg4g022130	0.3691	2.3364	0.2296	0.0984	0.0429	0.0573	0.0000	0.0777	0.0104	probable xyloglucan endotransglucosylase/hydrolase protein 23
Cg6g001050	1.6402	2.3611	1.8462	1.5071	2.2176	1.4802	1.2168	2.5911	1.2925	probable tyrosine-protein phosphatase At1g05000
Cg7g020850	8.5584	13.6152	7.6495	4.0628	8.2501	4.8119	3.2098	4.6364	3.0613	probable serine/threonine-protein kinase PBL18
Cg9g023190	0.0708	0.1835	0.1470	0.1392	0.2157	0.2110	0.1914	0.6225	0.3262	probable serine/threonine-protein kinase Cx32, chloroplastic
Cg2g034050	47.8859	42.4361	36.8626	34.6831	54.7714	52.7261	42.8540	104.4746	56.2779	probable ribose-5-phosphate isomerase 2
Cg3g024350	0.8745	0.8295	0.8836	0.8797	0.8215	1.2574	0.9756	3.2433	1.7787	probable receptor-like serine/threonine-protein kinase At5g57670
Cg2g044370	1.8653	3.1244	2.0172	4.6936	5.6336	2.7451	6.5358	15.5124	10.0314	probable purine permease 10
Cg4g025000	14.0568	16.2085	12.2319	16.0961	19.7411	15.6765	17.6203	36.5791	18.8492	probable protein phosphatase 2C 44
Cg5g002280	79.1774	68.8653	73.1412	19.1048	17.9279	21.8684	8.0853	3.6587	5.2930	probable polygalacturonase
Cg6g003640	1.8886	1.7033	2.0159	1.7728	1.1656	1.7027	0.9834	0.2865	0.5316	probable membrane-associated kinase regulator 4
Cg2g013510	0.2748	0.3293	0.0302	0.5485	0.7479	0.1029	0.0967	0.6825	0.2441	probable L-type lectin-domain containing receptor kinase VI.1
Cg1g024700	0.6293	0.4641	0.3691	2.6083	0.8169	1.4603	5.1473	3.4657	6.6305	probable LRR receptor-like serine/threonine-protein kinase At3g47570 isoform X1
Cg6g006680	2.9402	3.0341	1.5017	1.0352	1.4911	1.8276	0.6333	1.4512	1.0511	probable LRR receptor-like serine/threonine-protein kinase At3g47570 isoform X1
Cg6g006650	0.2822	0.7383	0.4095	0.2752	0.3651	0.3045	0.3073	0.2095	0.2601	probable LRR receptor-like serine/threonine-protein kinase At3g47570
Cg5g010750	0.5323	0.4606	0.4410	0.3314	0.3886	0.5372	0.1265	0.4629	0.2145	probable LRR receptor-like serine/threonine-protein kinase At1g74360
Cg2g028290	0.4073	0.6772	0.4883	0.4336	0.8597	0.4742	0.4571	0.9343	0.3690	probable LRR receptor-like serine/threonine-protein kinase At1g07650
Cg7g020350	2.2620	5.4891	2.8835	1.0888	2.1241	1.1366	0.3852	0.5899	0.4856	probable leucine-rich repeat receptor-like protein kinase At1g35710
Cg8g012480	0.2347	0.3450	0.2619	1.0965	0.6187	0.6805	1.0363	2.4075	1.4695	probable leucine-rich repeat receptor-like protein kinase At1g35710
Cg6g016580	0.0575	0.0216	0.0325	0.0709	0.1131	0.0638	0.1270	0.6319	0.3643	probable glycosyltransferase At5g03795
Cg3g015590	1.3742	1.2727	2.0020	3.0844	1.1626	1.9639	3.9649	2.6218	2.1856	probable glycerol-3-phosphate acyltransferase 3
Cg1g024200	9.6954	14.5480	8.5527	15.4115	15.6727	9.5453	12.4508	26.0828	14.1923	probable glutathione S-transferase
Cg9g012450	0.6651	0.3123	0.2220	0.1411	0.5843	0.3389	0.2736	0.5142	0.9151	probable galactinol--sucrose galactosyltransferase 2
Cg6g015900	0.2140	0.5958	0.2113	1.0490	0.9541	0.6964	1.2882	5.0164	2.4022	probable F-box protein At2g36090
Cg3g002150	0.2560	0.2610	0.3348	0.4202	0.3211	0.1576	0.0000	0.5184	0.1633	probable disease resistance protein RF9
Cg1g017080	0.2740	0.2829	0.2535	0.3515	0.4006	0.3386	0.2230	0.6594	0.3591	probable disease resistance protein At5g63020
Cg1g021240	0.1362	0.2208	0.2638	0.3289	0.3027	0.2760	0.2634	0.6523	0.3181	probable disease resistance protein At4g27220
Cg7g014650	1.9681	0.7426	1.5740	0.5071	0.9657	2.3261	0.4179	0.4512	0.5938	probable carboxylesterase 6
Cg3g021630	3.5052	4.2499	2.9674	4.0762	4.2842	3.0216	2.9979	6.0787	3.6245	probable carboxylesterase 2
Cg2g020360	10.1778	8.1531	6.5111	23.3438	19.1310	16.3080	24.4303	48.9026	27.8608	probable caffeine synthase 4
Cg2g020340	4.0022	3.8535	3.0729	10.5096	8.2525	5.8841	7.6688	17.0104	8.6120	probable caffeine synthase 4
Cg2g013140	0.0713	0.0195	0.0631	0.2051	0.0796	0.2044	0.8443	0.3729	0.5094	probable aminotransferase TAT2
Cg7g019610	1.5473	2.5227	1.7028	1.3477	2.2372	1.4341	1.7438	3.8374	2.0203	probable alpha,alpha-trehalose-phosphate synthase [UDP-forming] 9
Cg7g002040	9.0664	5.3185	0.5760	0.6944	4.7996	0.8729	0.2538	9.7966	3.1511	probable 2-oxoglutarate-dependent dioxygenase At5g05600
Cg4g022370	1.8096	1.6331	1.9900	0.5540	0.4203	0.7448	0.2324	0.0883	0.0878	probable 1-deoxy-D-xylulose-5-phosphate synthase 2, chloroplastic
Cg2g043690	0.3346	0.7419	0.1154	0.1186	0.0631	0.1722	0.0300	0.4650	0.0897	precursor of CEP14
Cg7g019370	14.1982	17.3556	15.4150	6.6631	9.6882	4.7678	2.2216	0.9990	0.9871	polygalacturonase 1 beta-like protein 3
Cg9g014680	0.3216	0.9366	0.5599	0.6787	0.7207	0.5765	1.0498	1.0633	0.7297	Polyadenylate-binding protein RBP47
Cg8g005470	0.3164	0.9614	0.3279	0.2400	0.3183	0.2751	0.4069	0.1890	0.2852	pollen-specific leucine-rich repeat extensin-like protein 3
Cg6g018680	7.1280	13.0920	6.3125	15.2821	17.5711	7.6901	10.4744	21.1512	11.5651	Plastid movement impaired 2
Cg5g042690	3.7963	3.4320	3.3198	1.3739	1.4769	1.4888	1.0723	0.4921	0.7291	plant UBX domain-containing protein 8
Cg2g029710	2.5738	2.1420	2.1279	2.0149	2.3271	2.3810	2.0941	4.3372	2.7829	plant UBX domain-containing protein 2
Cg5g031660	1.3513	2.7688	1.5040	1.6199	1.8216	1.0016	1.9650	2.6430	1.6861	plant cysteine oxidase 2-like
Cg3g013550	8.9269	22.0590	8.3114	20.4326	22.0086	11.3653	17.4957	7.5700	12.8732	phosphoglycerate mutase-like protein 1 isoform X2
CgUng003540	2.2187	5.0314	2.7257	4.0402	3.8024	1.8642	2.6145	1.9078	2.2528	pheophytinase, chloroplastic
Cg9g026080	0.8577	0.2279	0.3788	0.3753	0.5126	0.4844	0.0700	0.0088	0.0000	peroxidase 66
Cg2g001470	0.4203	0.8279	0.4509	0.7534	1.0854	1.1047	0.5199	1.1552	0.5826	peroxidase 15-like
Cg7g008640	0.5901	0.8937	0.6521	1.0702	1.1519	1.3104	0.7897	0.3850	0.5047	peptide-N4-(N-acetyl-beta-glucosaminyl)asparagine amidase A
Cg4g018580	0.4138	0.2889	0.2355	0.2123	0.2441	0.1599	0.1930	0.5474	0.2856	pentatricopeptide repeat-containing protein At4g33170
Cg2g009010	0.7185	0.7429	0.6845	0.4914	0.5990	0.4281	0.3312	0.7110	0.4120	pentatricopeptide repeat-containing protein At2g41080
Cg3g017500	1.6383	2.2895	1.3430	1.5424	1.4642	1.1606	0.9583	1.9177	1.2009	pentatricopeptide repeat-containing protein At1g43980, mitochondrial
Cg5g030620	0.5668	0.2694	0.4889	0.4397	0.4701	0.3649	0.4335	0.4110	0.4747	pentatricopeptide repeat-containing protein At1g09220, mitochondrial
Cg5g043520	0.3708	0.8968	0.5283	0.3428	0.4855	0.4584	0.4311	0.5217	0.4403	pectinesterase inhibitor 9-like
Cg2g038690	15.3049	19.6853	20.5790	5.3441	5.1389	5.0999	1.9071	0.6556	1.1051	patellin-4
Cg1g017700	9.8098	37.9092	11.4532	1.8313	7.8450	4.2636	0.3573	0.3025	2.6937	palmitoyl-monogalactosyldiacylglycerol delta-7 desaturase, chloroplastic
Cg3g023180	0.1665	0.4123	0.3699	1.5744	1.1366	0.8659	1.4961	1.1463	0.6260	omega-hydroxypalmitate O-feruloyl transferase
Cg1g001620	4.8211	12.8013	7.4115	3.1521	8.3682	5.4253	2.8821	4.5618	5.8736	oligopeptide transporter 7-like
Cg5g007900	9.4740	10.7213	8.8543	6.7659	7.7937	7.8877	6.2061	12.2449	6.4227	O-fucosyltransferase 30
Cg5g024620	0.2069	0.2083	0.1549	0.4194	0.3208	0.2368	0.3314	0.9050	0.4476	nudix hydrolase 13, mitochondrial-like
Cg6g019240	374.3542	158.0826	318.9373	3533.9396	1543.3097	1252.9927	3209.4225	2406.8949	2273.9076	non-specific lipid-transfer protein 2-like
Cg7g002330	0.1679	0.7928	0.3615	0.2667	0.0910	0.0000	0.0242	0.0716	0.0000	non-classical arabinogalactan protein 30
Cg8g000580	0.2529	0.0447	0.1944	0.1184	0.1198	0.5062	0.1908	0.0710	0.2498	NDR1/HIN1-like protein 13
Cg1g023040	2.3694	2.8185	2.7178	1.7480	2.4603	3.7662	2.8367	7.0661	3.8064	NAC domain-containing protein 7-like
Cg9g025080	0.4144	0.1698	0.3534	0.3251	0.2267	0.5794	0.1300	0.1266	0.0277	NAC domain-containing protein 7
Cg5g008680	0.1885	0.1874	0.3373	0.3637	0.2025	0.8864	0.3000	0.0209	0.0483	NAC domain-containing protein 7
CgUng001200	7.9346	3.7363	6.7327	13.4785	7.4113	6.5890	9.7960	4.3091	6.7135	---NA---
Cg4g006530	0.0000	0.5664	0.1549	0.4398	0.5722	1.2183	0.4603	0.3022	0.6825	---NA---
Cg2g022310	1.5842	3.3129	1.9208	1.9066	1.8597	1.3648	1.2593	0.5964	0.8211	---NA---
Cg4g007710	0.2585	0.7325	0.5518	0.4711	0.4823	0.4992	0.3052	0.3237	0.3372	---NA---
Cg6g019640	0.2331	0.9479	0.4726	0.3441	0.4521	0.0560	0.2276	0.1119	0.0591	---NA---
Cg2g045650	0.4118	0.0000	0.0000	0.2549	0.2305	0.0000	0.7150	0.2772	0.3066	---NA---
Cg7g016570	52.5473	22.6327	25.3540	42.1505	19.6326	20.7216	38.0615	16.4120	21.8056	---NA---
Cg5g031250	104.0062	247.5198	74.7953	26.5835	61.4544	33.7292	9.8349	16.3030	10.2360	---NA---
Cg1g004160	0.3808	0.6306	0.2834	0.4527	0.4116	0.1415	0.1132	0.4356	0.1754	---NA---
Cg4g017280	0.7254	1.5301	0.9686	2.8396	4.4795	1.7652	3.0211	1.2351	2.0693	---NA---
Cg7g018520	0.5680	0.2363	0.2829	0.2113	0.1336	0.8432	0.1326	0.7858	0.2466	---NA---
Cg4g021450	1.2693	2.6578	1.5553	2.0152	1.1006	0.1286	0.9161	0.2447	0.6565	---NA---
Cg3g025780	0.0832	0.5745	0.0358	0.0113	0.0000	0.0133	0.0657	0.0000	0.0000	---NA---
Cg5g014190	0.9427	1.8702	0.6912	0.9624	0.4509	0.1571	0.0482	0.6396	0.3277	---NA---
Cg5g028830	2.4995	2.6783	2.7514	3.8103	3.3387	2.1033	2.8475	1.0186	2.3364	---NA---
Cg6g003810	0.2457	0.1383	0.3086	0.3908	0.2351	0.2337	0.1085	0.5559	0.2807	---NA---
Cg8g021370	2.6733	1.5286	1.9038	1.3152	2.5548	1.8078	0.5022	0.1817	0.0385	Multicopper oxidase, type 1
Cg1g005440	27.3877	18.1651	3.0225	3.4530	23.2966	9.5155	3.9150	95.0408	44.4836	mitogen-activated protein kinase kinase kinase 18-like
Cg1g013160	3.3225	5.0109	0.1793	0.2273	1.8547	0.3469	0.0518	4.9295	1.1561	Mitochondrial distribution and morphology 12
Cg2g047240	0.3002	0.6872	0.3697	0.0499	0.0957	0.0847	0.0369	0.1197	0.0723	Methyltransferase type
CgUng006760	6.7927	10.3029	2.3988	0.5255	4.8147	0.4463	0.2714	6.3865	3.9929	methanol O-anthraniloyltransferase-like
Cg2g004680	1.0387	1.4459	0.2008	0.0664	0.2293	0.0064	0.0055	0.6431	0.2383	methanol O-anthraniloyltransferase-like
Cg4g009640	528.2377	668.8020	477.6199	2787.9557	1656.7115	1190.8049	1728.8626	3501.5152	3235.0256	metallothionein-like protein type 3
Cg1g021320	0.9779	2.0449	1.2891	0.6774	0.4026	0.3692	0.6388	2.1525	0.8014	metal transporter Nramp1
Cg4g017430	23.4772	39.3684	27.3359	18.9930	20.2331	8.0577	5.2216	1.6356	2.6243	Membrane lipoprotein
Cg2g034030	1.0093	1.1168	0.2330	0.1768	0.5884	0.2487	0.0796	0.5260	0.1281	Mediator of RNA polymerase II transcription subunit 18, putative
Cg6g008180	0.1481	0.4055	0.4052	0.5198	0.5652	0.4949	0.3025	0.3520	0.4931	mechanosensitive ion channel protein 10-like
Cg6g008190	3.0195	6.4954	4.0213	2.7373	6.0387	3.4769	2.3063	1.4305	3.2151	mechanosensitive ion channel protein 10-like
Cg2g003110	0.0381	0.0321	0.0524	0.0795	0.1442	0.0455	0.1493	0.5151	0.2474	MDIS1-interacting receptor like kinase 2
Cg5g003980	0.4955	0.4724	0.3509	0.5577	0.5476	0.6281	0.7044	1.6402	0.8500	MDIS1-interacting receptor like kinase 2
Cg2g016650	0.8106	0.7851	0.6301	0.3977	0.4853	0.3604	0.2377	0.5931	0.3127	MDIS1-interacting receptor like kinase 2
Cg8g000630	5.3038	10.9425	5.5740	2.7662	6.5403	3.8968	3.2312	5.2945	2.5854	lysM domain-containing GPI-anchored protein 2 isoform X1
Cg9g007970	1709.1158	837.5914	1531.4568	531.1799	493.3296	1129.8567	152.1113	62.0644	62.6751	LPXTG cell wall anchor domain-containing protein
Cg9g006780	0.4686	0.8047	0.9325	0.8391	0.3944	0.6147	0.6092	0.3705	0.4577	Lipase, class 3
Cg6g019310	0.3859	0.6128	0.3486	1.3008	1.0344	0.9129	1.4943	3.7789	2.0597	LEAF RUST 10 DISEASE-RESISTANCE LOCUS RECEPTOR-LIKE PROTEIN KINASE-like 2.4
Cg1g004350	2.1698	0.8806	1.5149	12.0973	4.9330	2.3049	4.8291	3.0095	3.5004	late embryogenesis abundant protein D-29
Cg1g016920	1.3754	0.6839	1.0838	1.5645	1.5644	1.3803	1.5800	0.9440	0.8942	Late embryogenesis abundant protein

Cg7g009390	3.6629	2.8771	3.4583	2.7644	2.9680	3.6238	3.2344	6.3924	3.4050	lst1 domain-containing protein
Cg1g013560	54.2954	162.6630	42.5883	34.1725	82.9555	41.6407	58.9202	54.1948	65.6747	inositol oxygenase 1-like
Cg7g019990	17.5134	18.4249	11.1356	8.8760	13.7566	7.4323	7.3713	25.9345	13.0812	IAA-amino acid hydrolase ILR1-like 1
Cg8g013870	0.0374	0.1757	0.0389	0.1635	0.1226	0.0000	0.0000	0.5317	0.1358	hypothetical protein EZV62_002173
Cg1g007380	2.6684	6.2805	3.7160	8.8834	6.0391	3.6953	3.9665	2.7523	3.4904	hypothetical protein CUMW_039830
Cg5g027070	0.5951	0.6123	0.6208	0.8153	0.4393	0.3095	0.1999	0.0406	0.0720	hypothetical protein CISIN_1g045582mg
Cg8g002690	1.3626	3.2221	1.8148	1.5859	1.3643	0.8125	0.6082	0.4206	0.3081	hypothetical protein CISIN_1g036613mg
Cg8g023110	1.3997	3.2387	2.2844	3.3507	2.9782	1.8111	0.7555	0.5829	0.6113	hypothetical protein CISIN_1g034155mg
Cg7g000020	16.2527	31.8112	11.7602	9.3133	23.4887	11.8085	7.7142	33.6866	14.6170	hypothetical protein CISIN_1g021888mg
Cg7g019830	0.3404	0.9971	0.5651	0.1566	0.2592	0.3393	0.1156	0.0432	0.2155	hypothetical protein CICLE_v10033710mg
Cg7g014370	5.0735	8.6222	8.6206	7.1751	3.1304	3.8467	2.9598	1.6146	2.3038	hypothetical protein CICLE_v10033499mg, partial
Cg2g002390	1.8440	0.7610	2.0840	5.6167	2.4318	3.4725	5.5413	2.9945	2.7355	hypothetical protein CICLE_v10015465mg
Cg6g006330	77.3446	56.8410	43.6993	44.2475	136.2566	69.8082	103.8381	77.5447	274.2486	hypothetical protein CICLE_v10012964mg
Cg9g002320	0.4994	0.6398	0.3386	0.1459	0.0745	0.1314	0.0167	0.2242	0.1013	hypothetical protein CICLE_v10007206mg
Cg9g011140	8.4118	2.2360	5.1901	2.0843	1.4337	3.3110	1.3006	0.2950	0.2522	hypothetical protein CICLE_v10005919mg
Cg9g010120	0.9614	0.8248	0.8694	0.2271	0.4937	0.3132	0.1731	0.2372	0.1359	hypothetical protein CICLE_v10004866mg
Cg3g017330	0.8010	0.7825	0.5702	0.6997	0.5722	0.6812	0.7035	2.2250	1.0545	hypothetical protein CICLE_v10003071mg
Cg3g013520	0.2067	0.2495	0.5107	0.4445	0.0991	0.2498	0.2123	0.0000	0.0597	Hydroxyproline-rich glycoprotein family protein
Cg7g022180	8.1934	11.6820	5.2362	7.6157	8.2965	5.5746	4.2211	9.9990	5.2126	Humj1 family protein
Cg9g005990	2.0506	4.8004	2.5691	3.1228	6.0966	3.4936	2.3482	2.8265	4.6827	homocysteine S-methyltransferase 3
Cg5g000500	0.4461	0.0712	0.2555	0.5581	0.1906	0.2751	0.6918	0.5116	0.5151	homeobox-leucine zipper protein ATHB-12-like
Cg9g022580	2.9326	2.9881	2.3128	2.2563	2.0293	3.0027	2.8377	5.7112	3.8376	histone-lysine N-methyltransferase, H3 lysine-9 specific SUVH5-like
Cg9g023350	0.2648	0.1312	0.2091	0.6025	0.4611	0.3626	0.7145	0.8054	0.6688	hexose carrier protein HEX6
Cg5g005270	0.9325	0.3668	0.7397	1.6896	1.4992	2.0682	2.7002	2.3472	2.7356	heme-binding protein 2-like
Cg5g019800	5.8707	12.1479	7.2720	0.9265	1.3434	1.4007	0.1988	0.1415	0.1191	heavy metal-associated isoprenylated plant protein 9
Cg1g004620	0.5051	0.1103	0.2979	0.3350	0.4106	0.1807	0.3904	0.1939	0.3989	heavy metal-associated isoprenylated plant protein 43-like
Cg5g002200	0.5176	0.5917	0.6601	1.5791	1.2927	1.0114	1.0987	2.4765	1.5865	heavy metal-associated isoprenylated plant protein 39
Cg2g028320	0.8098	1.1836	0.6895	1.5422	1.0352	0.6707	1.2711	2.4989	1.3129	heat stress transcription factor A-4b-like
CgUng021600	0.5778	0.4617	0.0624	0.6670	0.1282	0.2039	0.5405	0.2459	0.1129	heat shock protein 90-5, chloroplastic
Cg5g002260	3.8672	1.8466	3.5026	5.8267	3.0407	5.5548	8.7429	3.8227	7.0265	heat shock protein 83
Cg9g023970	236.2048	108.6616	159.3942	234.9481	67.8467	140.3099	214.5774	61.9345	195.8128	heat shock 22 kDa protein, mitochondrial isoform X2
Cg9g005340	1.8619	2.3834	1.3669	0.8220	1.3011	0.9358	0.6668	1.4906	0.8973	G-type lectin S-receptor-like serine/threonine-protein kinase LECRK3
Cg4g002220	2.3279	2.7931	1.9406	0.8152	0.6625	0.9312	0.4867	0.1368	0.2471	glycosyltransferase BC10-like
Cg4g008460	0.5337	0.5587	4.4377	0.0878	0.1026	0.0379	0.0000	0.0896	0.0136	glycine-rich cell wall structural protein-like
Cg9g018300	39.1442	18.6403	27.1656	88.8169	35.7028	56.3910	49.8294	22.1924	36.5577	glucan endo-1,3-beta-glucosidase, basic isoform-like
Cg2g039600	0.7312	2.2051	1.1969	1.1155	1.3517	0.6420	0.3296	0.2229	0.2357	glucan endo-1,3-beta-glucosidase 13
Cg5g004120	7.3349	5.9536	3.7440	2.7164	2.5424	2.7406	2.6423	6.4652	3.7556	glucan endo-1,3-beta-glucosidase
Cg2g044460	1.4694	0.6787	0.6463	2.9694	1.8912	2.6312	4.0336	9.7121	5.9479	glucan endo-1,3-beta-D-glucosidase-like
Cg5g015720	12.9934	20.8915	18.8938	4.3110	3.5968	2.0375	1.3142	0.3397	0.7634	glucan endo-1,3-beta-D-glucosidase-like
Cg1g029520	0.0824	0.7644	0.3677	0.0419	0.0479	0.0397	0.0373	0.0000	0.0000	glucan 1,3-beta-glucosidase A
Cg6g024880	16.4181	14.1871	18.0662	2.2280	1.3598	1.4473	1.2440	0.4880	0.7186	gibberellin-regulated protein 4
Cg2g040470	26.1909	23.5547	48.2682	17.8911	12.0462	16.5489	12.5893	6.0076	9.6311	gibberellin 2-beta-dioxygenase 8
Cg7g020130	2.4935	1.0431	1.8702	1.7168	2.2203	4.4737	0.8114	0.2272	0.1024	germin-like protein subfamily 1 member 1
Cg3g002480	0.1235	0.0345	0.1124	0.1644	0.1599	0.5198	0.1173	0.7917	0.2493	geranylgeranyl pyrophosphate synthase, chloroplastic-like
CgUng004340	7.9082	3.9270	7.9806	12.2131	7.1645	11.1274	8.8988	6.8465	8.1973	geraniol 8-hydroxylase-like
Cg2g045160	1.0133	2.1761	1.3889	1.0751	1.1378	0.8622	0.6909	0.5986	0.3603	Galactosyltransferase family protein isoform 1
Cg5g017000	6.2482	13.3458	6.5892	2.8088	5.5938	2.6808	1.8888	2.5562	1.6128	Formin-like protein 18
Cg5g043350	4.9613	3.2178	4.2645	6.6790	3.9491	4.4509	8.5469	3.3239	4.9747	Flower, cultured cell, putative
Cg6g013380	2.0856	1.9049	1.7385	1.6715	2.1051	1.1527	1.9018	4.5925	2.4796	F-box/kelch-repeat protein At2g44130-like
Cg1g015810	4.3913	2.1659	3.2522	5.6109	5.7038	5.4931	9.7311	9.4045	12.8728	F-box/kelch-repeat protein At1g80440
Cg1g005840	10.8085	12.0210	8.3486	10.0649	11.6428	10.5388	9.3011	19.0697	10.3850	F-box protein SKIP2
Cg6g005560	0.2199	0.9747	0.3658	0.4941	0.0541	0.2812	0.2857	0.1997	0.3646	F-box protein At5g49610-like
Cg6g011450	3.0303	1.4322	1.8710	4.1749	3.7532	3.3451	6.8497	2.7897	5.6957	F21M11.16 protein isoform 3
Cg8g006090	1.4644	1.0813	1.3807	1.6006	1.7363	1.9510	2.0737	4.2272	2.1410	extra-large guanine nucleotide-binding protein 1-like
Cg4g005590	4.7631	2.1251	3.9537	1.6688	2.7594	2.7413	2.5922	2.5307	1.7718	expansin-like B1
Cg8g000830	13.9297	28.1952	8.6314	7.1922	12.4149	5.3202	3.0934	6.6941	3.3751	expansin-like A2
Cg5g037790	1.6695	2.7945	0.9621	2.6804	2.5826	1.2681	1.6744	3.6328	1.8977	ethylene-responsive transcription factor ERF071
Cg5g041110	0.1561	0.6068	0.3613	0.1997	0.1170	0.1960	0.0684	0.1726	0.1008	ethylene-responsive transcription factor CRF5-like
Cg5g034050	17.1902	7.5481	1.7533	1.5017	7.3333	1.8014	0.8737	16.5753	3.9041	ethylene-responsive transcription factor 1B-like
Cg4g007180	5.7855	5.9416	4.7763	15.3334	6.8526	7.7362	9.0663	5.9545	6.3427	equilibrative nucleotide transporter 8
Cg3g000770	0.9394	0.9737	1.1927	3.2114	2.9678	2.0120	3.3946	8.2063	4.0624	ent-kaurene oxidase, chloroplastic-like
Cg2g036410	1.8729	2.4483	2.2923	1.2372	1.4022	1.1086	0.4498	0.1903	0.2695	Endosomal targeting BRO1-like domain-containing protein isoform 2
Cg8g008680	3.4395	3.5179	3.9079	1.3620	1.4165	0.9559	0.9003	0.4129	0.5747	endoglucanase 24-like
Cg9g004430	0.5168	0.6804	0.3694	0.8128	0.6748	0.4152	0.5131	0.9970	0.5442	endo-1,3;1,4-beta-D-glucanase-like
Cg5g036050	4.1750	6.3271	5.3834	5.6067	2.3647	2.4546	2.8987	2.5239	0.7166	E3 ubiquitin-protein ligase SP1-like
Cg2g020870	0.2975	0.2783	0.0191	0.0532	0.0942	0.2718	0.0000	0.7862	0.0410	dynein light chain, cytoplasmic-like
Cg5g008530	2.3822	1.6397	3.1591	2.0103	1.3387	2.5911	1.5912	0.7606	1.4717	DUF863 domain-containing protein
Cg1g010020	0.8001	0.9741	0.5846	0.5224	0.8975	0.5146	0.4554	1.0088	0.5094	DUF3511 domain-containing protein
Cg6g007930	0.3701	0.1817	0.3542	0.2277	0.1311	0.8846	0.6334	0.2003	1.3566	DNA-directed RNA polymerase subunit beta'
Cg9g023290	0.7320	1.0080	0.6067	1.1255	1.3249	0.4668	0.8970	1.8836	1.0651	derlin-1.2-like isoform X1
Cg5g041720	0.3250	0.7696	0.4781	1.2918	1.6758	1.2254	0.5336	0.2209	0.3550	delta-like protein A
Cg7g012810	0.9996	2.2072	0.7322	0.8152	1.3033	1.0363	0.5109	1.4771	0.7935	dCTP pyrophosphatase 1-like
Cg6g007900	0.2402	0.1848	0.2555	0.4457	0.2626	0.0390	0.1319	0.5644	0.2697	cytochrome P450 89A2-like
Cg5g027670	6.2149	5.6344	4.1476	9.8087	5.6105	5.8802	7.6726	17.7357	8.9568	cytochrome P450 87A3-like
Cg4g006870	0.9327	0.4661	0.9091	1.3714	1.5187	1.6337	2.0294	2.4391	1.6016	cytochrome P450 711A1
Cg2g040380	0.2248	0.5252	0.3397	0.5457	0.4437	0.1477	0.3499	0.2620	0.2680	cysteine-rich receptor-like protein kinase 10
Cg6g005410	4.4823	4.7258	1.7363	3.0737	5.1504	1.4656	2.3420	8.2850	3.4561	cysteine proteinase inhibitor
Cg9g009190	0.5595	0.2007	0.3540	0.7100	0.7896	0.1569	0.2068	0.1424	0.1746	Cyclopropane-fatty-acyl-phospholipid synthase
Cg3g025240	1.9555	2.3988	2.9333	0.9360	0.8726	0.8060	0.4762	0.2019	0.2565	cyclin-B2-4-like isoform X1
Cg4g001710	11.6012	5.0371	9.3512	15.5622	9.8817	15.3417	20.6683	24.3455	21.8883	coumarin 8-geranyltransferase 1b, chloroplastic
Cg8g023340	7.2756	5.6911	6.0826	6.0715	5.9282	7.9777	5.4792	12.6746	7.4591	copper transporter 1-like
Cg8g023380	2.3223	0.5805	2.6542	4.3373	2.3767	3.0691	0.4772	2.1788	1.6922	copper transporter 1
Cg1g013850	19.3397	58.4989	31.2117	16.2358	45.6661	26.5099	12.2344	10.0701	38.2389	clavaminate synthase-like protein At3g21360
Cg5g034970	0.5587	1.4220	0.4399	0.9009	1.1422	0.8737	1.1670	4.0480	3.0914	chlorophyllase-1, chloroplastic
Cg5g034980	75.6714	80.5336	54.4792	46.1601	87.2525	61.0090	45.9417	100.5161	62.6675	chlorophyllase-1, chloroplastic
Cg2g044430	971.7386	1152.5282	745.6397	206.3367	164.8411	257.5598	83.2535	38.1517	68.5351	chlorophyll a-b binding protein of LHCII type 1
Cg2g009840	1.7700	3.3249	2.4165	2.2935	2.0933	2.0188	2.6671	1.2857	2.3731	chaperone protein ClpC1, chloroplastic-like
Cg5g011170	35.0258	18.8666	24.6722	31.9437	14.9984	27.9017	63.6746	19.5714	45.1231	chaperone protein ClpB1
Cg3g016190	4.3567	12.5322	3.5873	0.4131	0.6774	0.2432	0.1582	0.0272	0.0910	chalcone synthase
CgUng003760	0.4457	0.0818	0.4538	0.4990	0.5061	0.8359	0.8889	0.2304	0.4692	chalcone synthase
Cg8g004230	5.1706	6.0652	4.4326	3.8489	4.6480	3.7389	3.4778	7.5383	3.7945	CBS domain-containing protein CBSX1, chloroplastic-like
Cg5g031750	4.2760	4.2624	5.0481	4.9329	6.6631	6.9953	4.8191	10.0787	7.5377	cationic amino acid transporter 2, vacuolar
Cg9g025950	8.2908	14.3008	6.3448	13.9910	13.8756	8.0429	11.1982	24.9944	12.7714	carboxylesterase 1-like
Cg4g016210	3.7117	4.1358	2.7064	3.6877	3.7037	3.0600	2.6820	5.5882	3.1557	calmodulin-binding transcription activator 4-like
Cg4g012170	10.1415	9.0981	9.5810	9.3149	10.8473	12.7507	9.9057	20.4925	13.0551	calcium-dependent protein kinase 2
Cg5g036790	7.2316	9.0149	8.9627	7.1899	6.9711	5.1080	5.5639	2.4841	3.9345	BTB/POZ domain-containing protein At1g03010 isoform X1
Cg5g007130	0.5527	0.4887	0.5058	0.4910	1.4807	1.1929	1.4485	3.3674	2.1522	beta-amylase 3, chloroplastic
Cg5g023510	1.6715	3.6526	3.4789	2.3249	2.3468	2.1719	2.0887	1.6874	1.2352	B-box zinc finger protein 23
Cg1g005220	0.3656	0.6363	0.3603	0.1338	0.2544	0.1655	0.1996	0.0538	0.1306	B-box zinc finger protein 19-like
Cg9g004560	8.4548	14.6219	8.3326	3.1009	6.2802	3.4945	2.9064	10.3596	1.5684	basic 7S globulin
CgUng000510	1.9363	3.3490	2.0234	0.9908	2.3775	1.2040	0.7966	0.7132	0.4674	auxin-responsive protein SAUR77
CgUng005220	23.0790	11.0900	22.2849	19.9510	23.4046	15.3686	3.7242	1.6843	3.1061	Auxin-responsive protein SAUR20
Cg3g023800	0.3460	0.8524	0.5604	0.2002	0.0912	0.0789	0.0202	0.0887	0.0118	auxin-induced protein 6B-like
Cg5g036140	2.7764	2.1123	2.6714	2.5854	1.7457	2.0227	2.6538	1.2558	2.1754	auxin-binding protein T85
Cg6g024950	3.0071	0.9113	1.8285	9.8430	2.2599	4.4199	29.8928	11.7046	15.1768	ATP-dependent zinc metalloprotease FTSH 6, chloroplastic
Cg4g001020	29.1174	19.9741	32.6758	35.2227	25.6235	32.7857	23.7862	11.3983	15.9441	aspartyl protease family protein At5g10770-like
Cg4g001000	57.8695	33.6842	62.7425	91.8406	74.5795	68.6126	85.3689	38.8656	56.6749	aspartyl protease family protein At5g10770-like
Cg6g019810	0.3797	0.8015	0.6548	0.1145	0.2123	0.1945	0.0373	0.1442	0.0521	AP2/ERF and B3 domain-containing transcription factor RAV1-like
Cg5g029170	41.2572	26.4691	41.1150	14.6515	9.7847	17.9386	13.8389	6.5357	7.5664	anthocyanidin 3-O-glucosyltransferase 2-like
Cg6g014140	2.9200	1.5524	3.9729	1.8355	1.6491	1.9488	2.1485	0.8739	1.2223	anthocyanidin 3-O-glucosyltransferase
Cg9g014580	15.0125	7.9832	19.8112	14.2781	9.1365	16.0719	18.0393	7.2035	13.1783	anthocyanidin 3-O-glucosyltransferase
Cg7g008510	0.3595	0.4778	0.5840	0.1513	0.1553	0.0881	0.1279	0.5140	0.2073	ankyrin repeat-containing protein BDA1
Cg6g003650	11.8817	16.0503	11.2955	8.8259	12.8139	12.2746	12.3031	24.8568	18.4344	ankyrin repeat-containing protein At5g02620-like

Cg6g013770	1.6308	0.7944	1.2557	2.9321	2.6394	1.5079	1.6681	1.7345	1.4137	Ankyrin repeat protein
Cg7g007040	2.5947	14.9794	3.4925	1.4260	3.7613	2.5615	1.0945	1.0458	1.1639	amino acid transporter AVT1C
Cg3g022060	56.6568	69.8690	39.9921	33.3611	38.2841	27.3654	25.4719	68.5035	38.1748	allene oxide synthase 1, chloroplastic
Cg2g041930	5.5775	2.4964	3.7309	10.4353	4.0332	3.4561	3.0169	6.5393	3.4495	allantoinase isoform X2
Cg2g003390	0.8293	0.2884	0.6474	0.6430	0.4679	2.2429	1.3157	1.2875	1.4044	aldehyde dehydrogenase family 3 member F1
Cg5g025360	1.7076	3.7700	2.1305	2.2140	1.9766	1.0929	0.8210	0.9684	0.7910	agamous-like MADS-box protein AGL11 isoform X4
Cg5g039220	3.0920	1.5095	2.7810	1.3451	1.5069	2.0174	0.4771	0.2595	0.2188	acyl transferase 4
Cg5g016110	0.2349	0.8554	0.5019	0.0000	0.1346	0.0330	0.0000	0.0000	0.0000	Activity-dependent neuroprotector homeobox protein
Cg9g023940	23.0632	28.3507	13.7778	17.5205	14.2628	14.5413	11.2487	22.8821	11.6137	acidic endochitinase-like
Cg6g016070	2.4485	2.4488	3.6173	1.8653	4.1801	6.9041	6.3961	14.6422	8.3389	ABC transporter G family member 34
Cg6g023500	0.9025	0.7511	0.8751	4.9497	1.8339	2.4487	6.6971	5.5249	8.5199	ABC transporter B family member 15-like
Cg8g007600	0.9700	1.5569	1.0022	0.7776	0.8158	0.6573	0.3292	0.9499	0.5719	AAA-ATPase At3g50940-like
Cg1g008570	2.8513	5.8466	3.1560	2.6857	2.5773	2.0814	1.4989	1.9489	0.8233	7-deoxyloganetin glucosyltransferase-like
Cg9g014240	4.1455	3.3769	4.4273	4.8425	3.2276	4.5261	5.5639	2.7445	4.8569	70 kDa peptidyl-prolyl isomerase
Cg2g033130	78.6550	53.6974	93.2877	36.4037	20.1108	37.5780	19.9677	8.7709	15.9746	3-oxo-Delta(4,5)-steroid 5-beta-reductase
Cg2g016350	0.6666	1.7362	0.7926	1.3919	1.1017	0.3074	0.8841	0.2611	0.4830	2-alkenal reductase (NADP(+)-dependent)-like
Cg5g023520	14.5912	4.4834	9.1155	10.0727	10.5527	2.7066	12.3101	2.7270	3.8625	22.0 kDa class IV heat shock protein-like
Cg8g023450	2.6389	1.3157	1.8199	2.8067	1.6866	1.5792	2.9201	1.1494	1.8026	18.2 kDa class I heat shock protein-like
Cg8g023420	360.9754	234.4735	207.2158	182.5366	138.7660	118.3194	150.8649	62.0511	90.6542	18.2 kDa class I heat shock protein-like
Cg7g000300	8.5317	1.7643	11.7859	14.9253	6.4008	10.4996	11.5695	6.1895	7.0167	18.1 kDa class I heat shock protein-like
Cg8g022260	59.3375	33.5162	33.8155	36.3694	33.6269	15.4797	52.5181	12.2573	19.6582	17.8 kDa class I heat shock protein-like
Cg6g005790	49.2109	29.6364	28.7753	47.7088	34.5536	16.3404	49.0621	18.8393	18.1868	17.6 kDa class I heat shock protein-like
Cg8g023430	452.9055	293.7711	383.5682	343.9619	196.6847	241.8688	298.7939	129.8491	253.4020	17.3 kDa class I heat shock protein-like
Cg2g033660	14.8299	11.8392	10.0425	13.7190	6.3418	7.8923	8.1727	2.9012	10.8863	10 kDa chaperonin, mitochondrial-like
Cg2g022810	2.3218	1.1615	1.9955	1.9676	1.2381	1.5115	0.8762	0.4270	0.9774	(-)-germacrene D synthase-like isoform X2
