# Supplementary material for: Comparative transcriptomic analysis on compatible/incompatible grafts in Citrus
Source: Hortic Res. 2022 Jan 19;9:uhab072. doi: 10.1093/hr/uhab072 (PMC8931943; doi:10.1093/hr/uhab072)
Supplement: Web_Material_uhab072 [file web_material_uhab072.zip › Table S8.pdf]

Table S8 Expression of genes in carotenoid biosynthesis

Name	GeneID	P1			P2			P3		
		Hm/Pt	Hm/Cj	Gx/Pt	Hm/Pt	Hm/Cj	Gx/Pt	Hm/Pt	Hm/Cj	Gx/Pt
GGPP (geranylgeranyl pyrophosphate synthase)	Cg1g003650	142.5017	149.0848	121.0550	126.6606	157.5194	136.9533	121.1912	181.6608	128.5053
	Cg8g001120	34.1284	33.7612	35.2163	29.4714	28.4383	34.4271	30.8373	22.4889	35.7605
	Cg6g018380	23.6420	24.8804	18.2390	22.5431	18.2388	9.5221	8.6138	6.1772	8.8212
	Cg6g018370	1.5786	1.5991	1.3824	2.0836	2.3387	2.0373	3.2507	3.6491	2.6106
	Cg6g018160	4.8817	6.6043	5.7279	4.3211	3.5113	1.8588	2.9595	2.4664	1.6041
	Cg6g018390	2.7245	3.0294	2.7051	3.8724	2.5367	1.9716	2.2498	2.3751	2.3774
	Cg6g018320	0.6501	0.5919	0.7034	0.5002	0.3063	0.3674	0.3472	0.5297	0.5350
	Cg8g009770	0.2856	0.4758	0.1582	0.4842	0.5410	0.1309	0.3188	1.0739	0.3493
	Cg3g002530	0.0000	0.1144	0.1723	0.2253	0.4487	0.1559	0.1426	1.0648	0.2378
	Cg3g002480	0.1235	0.0345	0.1124	0.1644	0.1599	0.5198	0.1173	0.7917	0.2493
PSY (phytoene synthase)	Cg6g016720	64.9583	74.5951	55.0064	65.3869	72.6672	53.4631	61.9532	61.4447	57.3331
	Cg2g000880	1.6876	1.3319	1.3512	1.9618	2.0137	0.7326	0.7156	1.2682	0.6010
PDS (phytoene desaturase)	Cg9g014150	6.6876	6.4756	7.0639	7.9913	7.7952	8.7929	8.8920	9.2885	10.1686
ZDS (ζ-carotene desaturase)	CgUng021180	5.9965	9.8734	6.8813	16.7172	12.6636	6.4570	7.6381	9.0517	5.9793
	Cg3g011400	1.8500	1.4540	2.8340	2.7251	2.4579	3.5636	3.8360	4.2388	3.2531
	Cg3g011340	1.3968	1.0758	2.2677	1.6569	1.7595	3.7226	3.3738	3.7680	4.3110
	Cg3g011600	1.7460	1.9272	1.6916	3.0598	2.6607	2.1740	2.2139	2.3793	2.3268
	Cg3g011420	0.7090	0.6997	0.8573	0.8118	0.7900	1.0200	1.0785	1.0969	1.2330
	CgUng021300	22.4033	32.7475	21.1287	31.7068	28.5967	16.8244	17.3632	18.3406	13.7617
β-LCY (lycopene β-cyclase)	Cg9g017190	21.5760	18.5736	23.6414	23.4087	27.8629	35.2187	31.7021	36.8181	43.1340
ε-LCY (lycopene ε-cyclase)	CgUng001590	8.9028	5.4603	11.0352	6.2744	7.6988	17.4204	12.7634	11.3826	17.2184
β-CHX (β hydroxylase)	Cg9g027460	128.7468	143.0289	96.0535	84.6678	71.8973	75.9123	65.8438	46.7553	72.0343
CCS (capsanthin-capsorubin synthase)	Cg8g013670	0.7377	1.3567	1.3032	0.9952	0.9417	1.3329	0.9964	0.6485	1.2399
NCED (9-cis-epoxycarotenoid dioxygenases)	Cg5g016320	73.0760	32.4938	23.1011	16.0330	29.3302	27.6383	6.8621	48.0417	4.9647
	Cg2g044950	9.5917	16.2093	4.0559	1.4294	6.8007	3.9827	0.5861	6.2712	1.4079
