# Supplementary material for: Comparative transcriptomic analysis on compatible/incompatible grafts in Citrus
Source: Hortic Res. 2022 Jan 19;9:uhab072. doi: 10.1093/hr/uhab072 (PMC8931943; doi:10.1093/hr/uhab072)
Supplement: Web_Material_uhab072 [file web_material_uhab072.zip › Supplementary Figure marker-up version.doc]

**Figure S1. Compatible / incompatible grafts growth and sampling strategy. a** Morphological difference between ‘Hongmian miyou’ (*Citrus maxima* (Burm.) Merrill, Hm) grafted onto trifoliate orange (*Poncirus* *trifoliate* (L.) Raf, Pt) (abbreviated Hm/Pt), Hm grafted onto Xiangcheng (*C. junos* (Sieb.) Tanaka, Cj) (abbreviated Hm/Cj) and ‘Guanxi miyou’ (Gx) grafted onto Pt (abbreviated Gx/Pt) at 112 DAG (days after grafting), 140 DAG, 161 DAG, 182 DAG and 203 DAG. Scale bars = 10 cm. **b** SPAD value, water potential and water content of leaves from summer shoots, and diameter, internode length and length of summer shoots. The box indicates the 25th and 75th percentiles. A line across the box represents the median. Whiskers are depicted as the maximum and minimum values and black circles represent outliers. Columns with bars represent means ± SE (*n* > 10). Asterisks represent remarkable differences compared to control (**p* < 0.05, ***p* < 0.01), analysed using Student’s t-test.

**Figure S2.** **The trends of mineral elements levels in leaves.** Columns with bars represent means ± SE (*n* > 10). Asterisks represent remarkable differences compared to the control (**p* < 0.05, ***p* < 0.01), analysed using Student’s t-test.

**Note:** Cu: copper, Na: natrium, Mn: manganese, Mg: magnesium, Fe: iron, P: phosphorus, Ca: calcium, N: nitrogen, Zn: zinc, B: boron.

**Figure S3. The trends of phytohormone levels in leaves.** A single asterisk (*) represents statistically significant differences (*p*< 0.05), double asterisks (**) represent highly statistically significant differences (*p*<0.01), analysed using Student’s t-test.

**Note:** ABA: abscisic acid, IAA: indole-3-acetic acid, GA_3_: gibberellin A3, JA: jasmonic acid, SA: salicylic acid.

**Figure S4.** **Transverse section of leaves.** The bar in photomicrographs is 100 µm and the bar in photo of leaves is 1 cm.

**Figure S5. Analysis of DEGs from pairwise comparison between the different development stages at the same graft combination. a** Venn diagrams of DEGs. **b** KEGG enrichment analysis of the DEGs. **c** Expression of DEGs in carotenoid biosynthesis and plant hormone signal transduction pathways. Heatmap color indicates FPKM value.

**Figure S6. KEGG functional categories enriched by different co-expression modules.** Only significant categories (*p* < 0.05) are displayed.

**Figure S7. Relationships of modules and different traits.** Each row in the table corresponds to a module, and each column corresponds to a trait.

**Figure S8. GO functional categories enriched by different co-expression genes in green module.**

**Figure S9. Analysis of DEGs from pairwise comparison between the scions from different rootstocks. a** KEGG enrichment analysis of the DEGs. **b** Expression of transcription factors. Heatmap color indicates log_10_(FPKM+0.01) value.

**Figure S10. Hub genes selected from green module.**
